# The accumulation of methylglyoxal and acrolein impairs arginine homeostasis causing hyperglycemia and renal abnormalities in male zebrafish

**DOI:** 10.1038/s41467-026-76082-6

**Published:** 2026-07-28

**Authors:** Shu Li, Hao Li, Xin Zhang, Rui Ge, Katrin Bennewitz, Gernot Poschet, Michael Buettner, Thomas Fleming, Ingrid Hausser, Julia Szendroedi, Peter Paul Nawroth, Jens Kroll

**Affiliations:** 1 Department of Vascular Biology and Tumor Angiogenesis, European Center for Angioscience (ECAS), Medical Faculty Mannheim, Heidelberg University, Mannheim, Germany; 2 German Cancer Research Center (DKFZ) Unit D400, Heidelberg, Germany; 3 Metabolomics Core Technology Platform, Centre for Organismal Studies, Heidelberg University, Heidelberg, Germany; 4 Cluster of Excellence GreenRobust, Heidelberg University, Heidelberg, Germany; 5 Department of Internal Medicine I and Clinical Chemistry, Heidelberg University Hospital, Heidelberg, Germany; 6 Institute of Pathology IPH, EM Lab, Heidelberg University Hospital, Heidelberg, Germany; 7 Medical Clinic and Polyclinic II, University Hospital Dresden, Dresden, Germany

**Keywords:** Kinases, Diabetes complications

## Abstract

Reactive carbonyl species contribute to diabetes and its complications, but how the interconnections of carbonyl-detoxifying enzymes regulate metabolic homeostasis through arginine metabolism remains unclear. Here we generate zebrafish lacking both glyoxalase 1 and aldo-keto reductase 1A1A, two major enzymes that detoxify methylglyoxal and acrolein. Double deficiency causes carbonyl accumulation, suppresses arginine metabolism, and impairs insulin signaling, resulting in elevated glucose levels in larvae and in postprandial hyperglycemia in adult male zebrafish. These metabolic alterations are accompanied by glomerular basement membrane thickening and podocyte effacement, whereas retinal vasculature remains unaffected. Arginine supplementation restores Akt phosphorylation, improves insulin signaling, and attenuates renal pathology, indicating that disrupted arginine metabolism mediates the metabolic consequences of carbonyl stress. Our findings identify glyoxalase 1 and aldo-keto reductase 1A1A as cooperative regulators of carbonyl detoxification and reveal a carbonyl–arginine axis linking reactive carbonyl accumulation to impaired insulin signaling, hyperglycemia, and tissue-specific diabetic injury.

## Abstract (web summary)

Reactive carbonyl species contribute to diabetes and its complications. Here the authors used zebrafish lacking both glyoxalase 1 and aldo-keto reductase 1A1A to show carbonyl accumulation and elevated glucose level.

## Introduction

Growing evidence supports a strong causative role between the accumulation of reactive carbonyl species (RCS), the development of ageing, cardiovascular diseases, diabetes, diabetic complications, and insulin resistance^1–4^. Among RCS, methylglyoxal (MG) is a highly reactive dicarbonyl compound generated from glycolysis, gluconeogenesis, lipid peroxidation, and the degradation of glycated proteins^5^. MG is recognized as a major precursor of advanced glycation end products (AGEs) and a potent inducer of dicarbonyl stress^6^. It reacts with arginine residues to form methylglyoxal-derived hydroimidazolones (MG-Hs), argpyrimidine (AP), and tetrahydropyrimidine (THP), thereby altering protein structure and function^7^. Under physiological conditions, MG is mainly detoxified through the glyoxalase system, consisting of glyoxalase I (GLO1) and glyoxalase II (GLO2), which convert MG into S-D-lactoylglutathione and D-lactate. However, additional enzymatic systems, including aldo-keto reductases (AKRs) and aldehyde dehydrogenases (ALDHs), also contribute to MG detoxification. AKRs can reduce MG to either acetol or lactaldehyde, and in particular aldo-keto reductase 1A1A (AKR1A1A) participates in the detoxification of the reactive aldehyde acrolein (ACR)^8,9^, which can form propano adducts with the guanidine group of arginine. This subsequently impairs protein function and reduces the availability of arginine^10^. Recent studies have identified distinct and shared metabolite profiles associated with diabetic microvascular complications, indicating that each complication is characterized by specific metabolic alterations as well as overlapping pathogenic pathways^11^. Similarly, a simultaneous accumulation of MG and ACR may reflect the interdependent network of enzymatic activities responsible for RCS detoxification and the multiple pathways leading to RCS generation, which together may reveal the underlying pathophysiological mechanisms of microvascular complications in diabetes.

Arginine is a conditionally essential amino acid that plays a crucial role in maintaining metabolic health^12^. It can be synthesized from glutamine, glutamate, and proline, and serves as an indispensable substrate for the urea cycle, protein synthesis, creatine and polyamine production, and nitric oxide (NO) generation^13^. Beyond these fundamental metabolic functions, arginine exerts multiple beneficial effects on cardiovascular health, immune function and glucose homeostasis^14–16^. Arginine has been shown to promote insulin secretion from pancreatic β-cells via membrane depolarization and calcium influx^17–19^. In addition, it serves as a precursor for NO, which enhances insulin sensitivity and improves endothelial function^20,21^. Moreover, it contributes to polyamine biosynthesis, a pathway implicated in cell proliferation, differentiation and growth^22^. Arginine can also enhance the release of incretin hormones such as glucagon-like peptide-1 (GLP-1) and growth hormone, leading to amplify insulinotropic effects, growth and development^23^. Clinical and experimental studies have further shown that arginine supplementation lowers fasting glucose levels and improves glycemic control, highlighting its critical role in maintaining glucose balance and vascular health^14,24–26^. Conversely, disturbances in arginine metabolism have been associated with endothelial dysfunction, impaired immune system, delayed wound healing, insulin resistance, and an increased risk of type 2 diabetes mellitus (T2DM)^27–30^.

Previous research has linked decreased GLO1 activity to diabetes, diabetic complications, aging, obesity, cardiovascular diseases, and neurodegenerative disorders^31,32^. In zebrafish, GLO1 knockout was found to moderately increase MG levels without causing any vascular defects under normal feeding conditions. This suggests that alternative enzymatic systems, including AKRs and ALDHs, can partially compensate for GLO1 loss^33,34^. Similarly, in GLO1-deficient Schwann cells, AKR-mediated MG detoxification was upregulated, further supporting this compensatory role^35^. A single knockout study in zebrafish has revealed that AKR1A1A preferentially metabolizes ACR^9^, and that elevated ACR levels in *akr1a1a* mutants led to insulin resistance and the development of characteristics of diabetic nephropathy and retinopathy^9^. However, it remained unknown whether AKR1A1A sufficiently compensates for GLO1 loss, or vice versa, or whether the combined loss of GLO1 and AKR1A1A alters arginine metabolism and aggravates hyperglycemia and renal abnormalities.

This study aimed to address these questions by generating GLO1/AKR1A1A double knockout (DKO) zebrafish and examining the simultaneous accumulation of MG and ACR, and their subsequent impact on arginine metabolism, glucose homeostasis and kidney morphology. The results highlight the compensatory role of AKR1A1A in the absence of GLO1 and provide insight into how distinct RCS profiles drive organ-specific complications, offering a metabolic basis for personalized therapeutic strategies in diabetes and metabolic diseases.

## Results

### GLO1/AKR1A1A DKO larvae exhibited increased MG and ACR levels simultaneously

To investigate the RCS detoxification network based on the various detoxifying enzymes, and to evaluate the net effect when both are knocked out, we generated zebrafish deficient for both GLO1 and AKR1A1A, using strategies previously reported^9,33,36^. Initially, GLO1KO zebrafish were crossed with AKR1A1AKO zebrafish to obtain double heterozygous (DHET) offspring that displayed normal gross morphology. These DHET zebrafish were then intercrossed to produce DKO and the corresponding wildtype lines for subsequent maintenance and experimental use (Fig. S1A). The DKO line was verified by genotyping, with sequencing chromatograms of PCR products encompassing the target regions of *glo1* and *akr1a1a* confirming the expected mutations (Fig. S1B, C). The resulting DKO zebrafish were viable and displayed normal gross morphology (Fig. S1D). Furthermore, their length and weight were comparable to those of the control groups (Fig. S1E, F). Collectively, these data confirmed the successful establishment of the GLO1/AKR1A1A DKO zebrafish model.

**Fig. 1:**
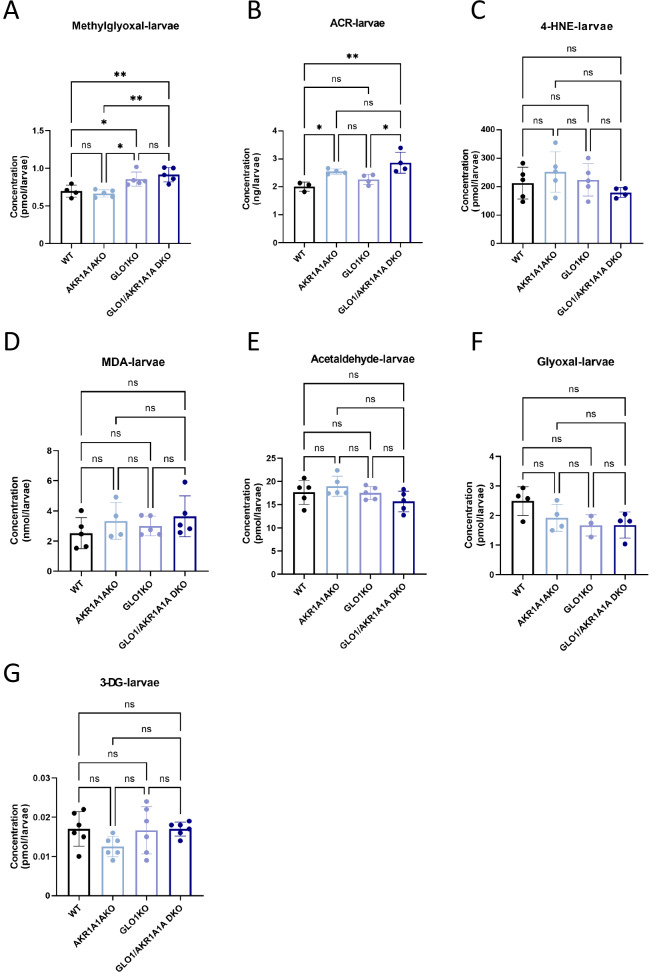
GLO1/AKR1A1A DKO larvae exhibited increased MG and ACR levels simultaneously. **A** MG levels were increased in GLO1/AKR1A1A DKO larvae compared to WT and AKR1A1AKO larvae. *n* = 4/5 biological replicates per group. Exact *p* values for each comparation are: WT vs. GLO1KO, *p* = 0.048; WT vs. DKO, *p* = 0.006; AKR1A1AKO vs. GLO1KO, *p* = 0.0108; AKR1A1AKO vs. DKO, *p* = 0.0012. MG, methylglyoxal. **B** ACR levels were increased in GLO1/AKR1A1A DKO compared to WT and GLO1KO larvae. *n* = 3/4 biological replicates per group. Exact *p* values for each comparation are: WT vs. AKR1A1AKO, *p* = 0.0491; WT vs. DKO, *p* = 0.0027; GLO1KO vs. DKO, *p* = 0.0181. ACR, acrolein. **C–E** 4-HNE, MDA and acetaldehyde concentrations were unchanged in DKO larvae compared to WT, GLO1KO and AKR1A1AKO larvae. *n* = 4/5 biological replicates per group. 4-HNE, 4-hydroxynonenal; MDA, malondialdehyde. **F**,**G** Glyoxal and 3-DG levels were unchanged in DKO larvae compared to WT, GLO1KO and AKR1A1AKO larvae. *n* = 6 biological replicates per group. 3-DG, 3-deoxyglocosone. The bars indicate mean ± SD values. Statistical analysis was performed by one-way ANOVA. ns, not significant, *p < 0.05, **p < 0.01. Source data are provided as a Source Data file.

To explore the carbonyls stress in GLO1/AKR1A1A DKO zebrafish, we quantified several classical RCS that are detoxified by GLO1 and AKR enzymes^5,9,37–39^. The results showed that levels of both MG and ACR were significantly higher in DKO larvae compared to WT (Fig. 1A, B). Consistent with our previous reports, MG levels were moderately increased in GLO1KO zebrafish^33^, and ACR levels were significantly elevated in AKR1A1AKO zebrafish (Fig. 1A, B)^9^. However, the levels of other metabolites, including 4-hydroxynonenal (4-HNE), malondialdehyde (MDA), acetaldehyde (AA), glyoxal and 3-deoxyglocosone (3-DG) (Fig. 1C–G) were unchanged among the four groups. Therefore, the combined loss of GLO1 and AKR1A1A led to the accumulation of MG and ACR simultaneously.

### Deceased arginine levels in GLO1/AKR1A1A DKO zebrafish and larvae co-treated with MG and ACR through the formation of MG-H1

**Fig. 2:**
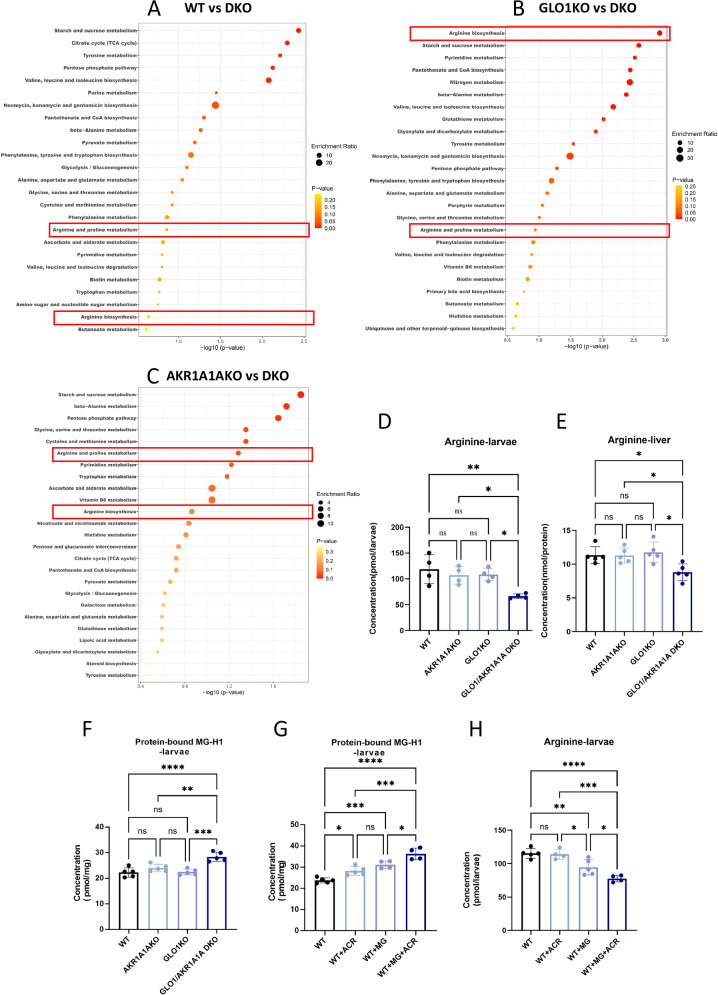
Deceased arginine levels in GLO1/AKR1A1A DKO zebrafish and larvae co-treated with MG and ACR through the formation of MG-H1. **A** Enrichment analysis of the significantly altered metabolites displayed top metabolic pathways in DKO larvae compared to WT group. *n* = 4 biological replicates per group. **B** Enrichment analysis of the significantly altered metabolites displayed top metabolic pathways in DKO larvae compared to GLO1KO group. *n* = 4 biological replicates per group. **C** Enrichment analysis of the significantly altered metabolites displayed top metabolic pathways in DKO larvae compared to AKR1A1A group. *n* = 4 biological replicates per group. The red frame represents arginine-related pathways. Enrichment analysis was performed by MetaboAnalyst 5.0 based on KEGG database. *P* values were calculated using a one-sided hypergeometric over-representation test (ORA). **D**,**E** Arginine levels were the lowest in DKO larvae and liver tissues compared to WT, GLO1KO and AKR1A1AKO groups. *n* = 4/5 biological replicates per group. Exact *p* values for each comparation are: **D** WT vs. DKO, *p* = 0.0076; AKR1A1AKO vs. DKO, *p* = 0.0436; GLO1KO vs. DKO, *p* = 0.0344. **E** WT vs. DKO, *p* = 0.0318; AKR1A1AKO vs. DKO, *p* = 0.0345; GLO1KO vs. DKO, *p* = 0.0105. **F** Protein-bound MG-H1 levels in GLO1/AKR1A1A DKO larvae were the highest compared to WT, GLO1KO and AKR1A1AKO groups. *n* = 5 biological replicates per group. Exact *p* values for each comparation are: WT vs. DKO, *p* < 0.0001; AKR1A1AKO vs. DKO, *p* = 0.0023; GLO1KO vs. DKO, *p* = 0.0001. **G** Protein-bound MG-H1 levels were increased in WT larvae treated with ACR, MG, or both, with the highest levels observed in the co-treatment group. *n* = 4/5 biological replicates per group. Exact *p* values for each comparation are: WT vs. WT + ACR, *p* = 0.0151; WT vs. WT + MG, *p* = 0.0004; WT vs. WT + MG + ACR, *p* < 0.0001; WT + ACR vs. WT + MG + ACR, *p* = 0.0002; WT + MG vs. WT + MG + ACR, *p* = 0.0119. MG-H1, methylglyoxal-derived hydroimidazolone 1; ACR, acrolein; MG, methylglyoxal. **H** Arginine levels were significantly reduced in larvae co-treated with MG and ACR compared to those treated with either compound alone. *n* = 4/5 biological replicates per group. Exact *p* values for each comparation are: WT vs. WT + MG, *p* = 0.0079; WT vs. WT + MG + ACR, *p* < 0.0001; WT + ACR vs. WT + MG, *p* = 0.0186; WT + ACR vs. WT + MG + ACR, *p* = 0.0001; WT + MG vs. WT + MG + ACR, *p* = 0.0421. The bars indicate mean ± SD values. Statistical analysis was performed by one-way ANOVA. ns, not significant, *p < 0.05, **p < 0.01, ***p < 0.001, ****p < 0.0001. Source data are provided as a Source Data file.

Given the observed accumulation of reactive carbonyl species and indications of disturbed arginine metabolism from literature, targeted metabolites were analyzed by GC/MS in WT, GLO1KO, AKR1A1AKO and GLO1/AKR1A1A DKO larvae. Partial least squares-discriminant analysis (PLS-DA) revealed distinct clustering of the groups based on their metabolic profiles, reflecting significant group-specific variations (Fig. S2A). Subsequently, fold change analysis was performed to identify differential metabolites between each pair of groups (Fig. S2B–D). Enrichment analysis of notably altered metabolites consistently highlighted changes in arginine metabolism in comparisons between DKO and WT (Fig. 2A), DKO and GLO1KO (Fig. 2B), and DKO and AKR1A1AKO (Fig. 2C). Further quantification of arginine levels via UPLC/MS in larvae and organs revealed that DKO larvae and liver tissues had the lowest arginine levels compared to WT, GLO1KO, and AKR1A1AKO zebrafish (Fig. 2D, E), while arginine levels in muscle remained unchanged (Fig. S3A). Additionally, combined nitrite and nitrate concentrations were shown to be decreased in DKO larvae compared to WT (Fig. S4A).

To determine the interaction between arginine metabolism and the combined loss of GLO1and AKR1A1A, as well as the accumulation of MG and ACR, the free arginine, protein-bound methylglyoxal-derived hydroimidazolone 1 (MG-H1) and protein-free MG-H1 levels were measured in WT, GLO1KO, AKR1A1AKO and GLO1/AKR1A1A DKO zebrafish, as well as in larvae co-treated with MG and ACR. The analysis revealed that protein-bound MG-H1 and protein-free MG-H1 levels were markedly elevated in both GLO1/AKR1A1A DKO larvae and in those co-treated with MG and ACR (Fig. 2F, G and Fig. S4B, C). This was accompanied by a significant reduction in free arginine levels (Fig. 2H). Additionally, L-carnosine treatment, which acts as a scavenger of methylglyoxal and acrolein, reversed the decrease in arginine levels in DKO larvae (Fig. S4D), indicating that carbonyl stress is the main cause of arginine modification. However, the arginine levels in carnosine-treated DKO mutants remained lower than in the wild-type controls, suggesting the involvement of other potentially regulated pathways. We therefore measured the transcript levels of genes involved in stress-activated and arginine metabolism pathways. DKO larvae showed increased expression of activating transcription factor 4a *(atf4a)*, activating transcription factor 4b (*atf4b)* and argininosuccinate lyase (*asl)*, decreased expression of argininosuccinate synthase 1 (*ass1)* and ornithine decarboxylase 1 (*odc1)*, and unchanged expression of arginase 1 (*arg1)* (Fig. S4E–J). Moreover, arginine-related metabolites exhibited differential changes (Fig. S3B). Ornithine tended to decrease, while spermidine and putrescine were significantly reduced (Fig. S4K–M). Collectively, these findings indicated that the combined loss of GLO1 and AKR1A1A, along with the concurrent accumulation of MG and ACR, leads to arginine depletion via increased protein glycation and altered arginine metabolism.

### Downregulated insulin signaling pathway in GLO1/AKR1A1A DKO zebrafish compared to WT

**Fig. 3:**
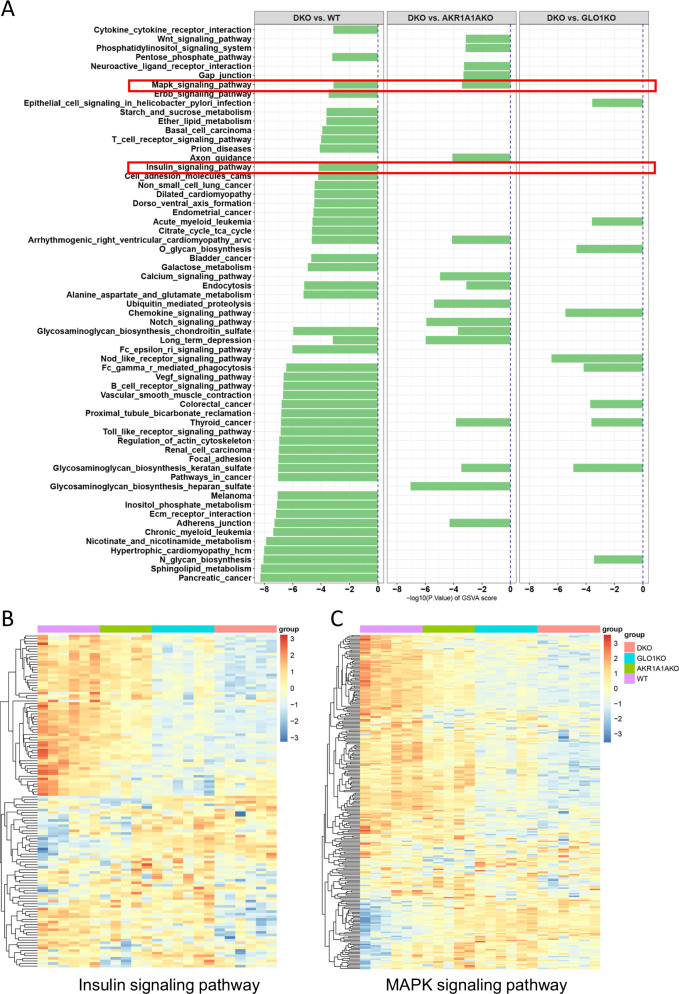
Downregulated insulin signaling pathway in GLO1/AKR1A1A DKO zebrafish compared to WT. **A** GSVA of RNA-sequencing data revealed the significantly downregulated pathways in pairwise comparisons (DKO vs WT, DKO vs AKR1A1AKO, DKO vs GLO1KO) at 96hpf. *n* = 5/6 biological replicates per group. The red frame represents insulin signaling and its downstream pathway. GSVA enrichment scores were compared between groups using a two-sided empirical Bayes moderated *t*-test implemented in the limma package. Plotted values represent -log10 of the unadjusted two-sided *P* values. GSVA, Gene Set Variation Analysis; hpf, hours of post fertilization. **B** Heat map of relative mRNA expression in the insulin signaling pathway gene set. Orange indicates higher expression and blue indicates lower expression. **C** Heat map of relative mRNA expression in MAPK signaling pathway gene set. Orange indicates higher expression and blue indicates lower expression.

RNA sequencing was also conducted in order to determine the role of GLO1/AKR1A1A in the altered arginine metabolism. Principal component analysis (PCA) revealed distinct separation among the groups (Fig. S5A). Correlation analysis showed strong similarity within each group and comparatively lower correlations between groups (Fig. S5B). Based on these observations, differential gene expression (DGE) analysis was performed for each pairwise group comparison (Fig. S5C–E). Next, gene set variation analysis (GSVA) was conducted using the Kyoto Encyclopedia of Genes and Genomes (KEGG) database to identify significantly altered pathways across the groups (Fig. 3A and Fig. S5F). Among the significantly altered pathways, the insulin signaling pathway and its downstream MAPK signaling pathway (Fig. 3B, C), which are closely correlated with arginine metabolism^14^, were significantly downregulated in DKO larvae compared to WT. This suggested that the altered arginine metabolism in GLO1/AKR1A1A DKO impairs insulin signaling.

### Tissue-specific arginine metabolism, impaired insulin signaling and glucose metabolism in GLO1/AKR1A1A DKO zebrafish

**Fig. 4:**
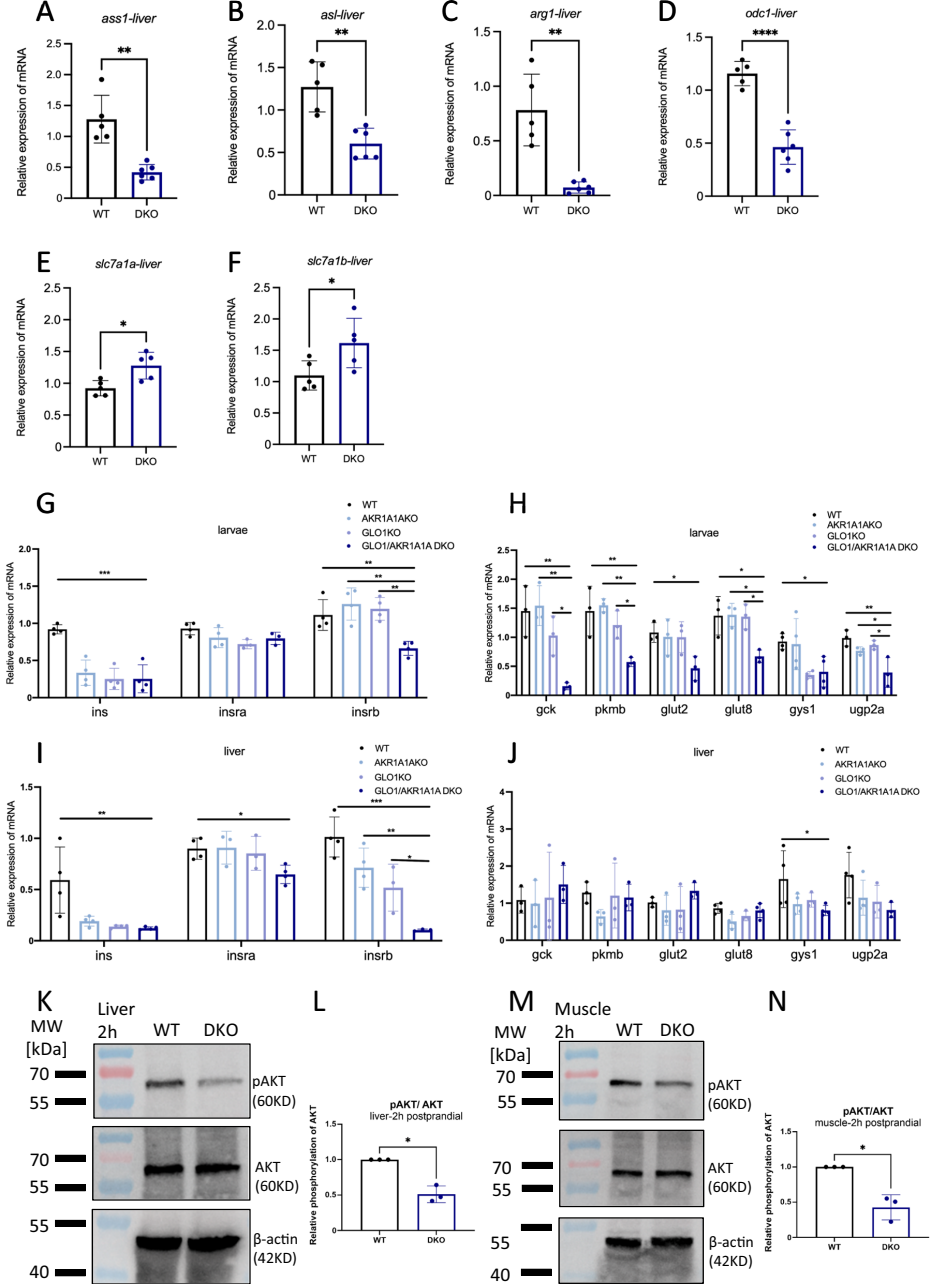
Impaired arginine and glucose metabolism in GLO1/AKR1A1A DKO zebrafish tissues. **A–F** Relative mRNA expression levels of genes involved in arginine synthesis (**A**, **B**), and arginine catabolism were downregulated (**C**, **D**) and arginine transport (**E**, **F**) were upregulated in DKO liver tissues compared to WT. *n* = 5/6 biological replicates per group. ass1, argininosuccinate synthase 1; asl, argininosuccinate lyase; arg1, arginase 1; odc1, ornithine decarboxylase 1; slc7a1a, solute carrier family 7 member 1a; slc7a1b, solute carrier family 7 member 1b. Exact *p* values for each comparation are: ass1, *p* = 0.0059; asl, *p* = 0.0038; arg1, *p* = 0.0079; odc1, *p* < 0.0001; slc7a1a, *p* = 0.0155; slc7a1b, *p* = 0.0419. **G, H** Relative mRNA expression levels of insulin-related genes (**G**), and genes involved in glucose transport, glycolysis and glycogenesis (**H**) were significantly altered in DKO larvae. *n* = 3/4 biological replicates per group. ins, insulin; insra, insulin receptor a; insrb, insulin receptor b; gck, glucokinase; pkmb, pyruvate kinase M1/2b; glut2, glucose transporter 2; glut8, glucose transporter 8; gys1, glycogen synthase 1; ugp2a, UDP-glucose pyrophosphorylase 2a. **I**, **J** Relative mRNA expression levels of insulin-related genes (**I**), and genes involved in glucose transport, glycolysis and glycogenesis (**J**) were significantly altered in DKO liver tissues. *n* = 3/4 biological replicates per group. mRNA expression levels were quantified by RT-qPCR and normalized to *arnt 2*. arnt2, aryl hydrocarbon receptor nuclear translocator 2. **K–N** Reduced postprandial AKT phosphorylation in liver and muscle of DKO zebrafish. *n* = 3 biological replicates per group. Exact *p* values for each comparation are: L, *p* = 0.0189. N, *p* = 0.031. The bars indicate mean ± SD values. Statistical analysis were applied by a two-tailed Student’s t-test and one way ANOVA. *p < 0.05, **p < 0.01, ***p < 0.001, ****p < 0.0001. Source data are provided as a Source Data file.

To explore the different alterations of arginine levels in liver and muscle tissues, the genes involved in arginine synthesis - *ass1*, *asl*, arginine catabolism - *arg1*, *odc1* and arginine transport - solute carrier family 7 member 1a (*slc7a1a*), solute carrier family 7 member 1b (*slc7a1b*) were examined by performing RT-qPCR. In the liver tissues, *ass1*, *asl*, *arg1* and *odc1* were significantly downregulated, accompanied by a compensatory upregulation of *slc7a1a* and *slc7a1b* (Fig. 4A–F). In contrast, no significant changes were observed in muscle tissues (Fig. S3C–F).

To validate the RNA sequencing results, the transcript levels of insulin signaling molecules including insulin (*ins*), insulin receptor a (*insra*), insulin receptor b (*insrb*), as well as genes involved in glucose transport—glucose transporter 2 (*glut2*), glucose transporter 8 (*glut8*), glycolysis—glucokinase (*gck)*, pyruvate kinase M1/2b (*pkmb*) and glycogenesis—glycogen synthase 1 (*gys1)*, UDP-glucose pyrophosphorylase 2a (*ugp2a*) were also quantified by RT-qPCR in larvae and liver. In DKO larvae, *ins, glut2* and *gys1* expression were decreased compared to WT larvae, while *insrb, gck*, *pkmb*, *glut8*, and *ugp2a* expression were significantly decreased compared to WT, GLO1KO, and AKR1A1AKO larvae (Fig. 4G, H). In DKO liver tissues, *ins*, *insra* and *gys1* expression were downregulated compared to the WT group, while *insrb* expression was downregulated compared to the WT, GLO1KO, and AKR1A1AKO groups (Fig. 4I, J).

To investigate the insulin signaling in liver and muscle tissues under fasting and 2 h postprandial conditions at the protein levels, the p-AKT/AKT was analyzed by Western blotting. The results showed that the p-AKT/AKT was reduced in the livers and muscles of DKO zebrafish under postprandial conditions (Fig. 4K–N), but remained unchanged under fasting conditions (Fig. S3G–J). Overall, these results suggested that the combined loss of GLO1 and AKR1A1A disrupts glucose metabolic pathways.

### Aggravated hyperglycemia, thickened glomerular basement membrane (GBM) and increased podocytes effacement in Tg(fli1:EGFP) GLO1/AKR1A1A DKO zebrafish

**Fig. 5:**
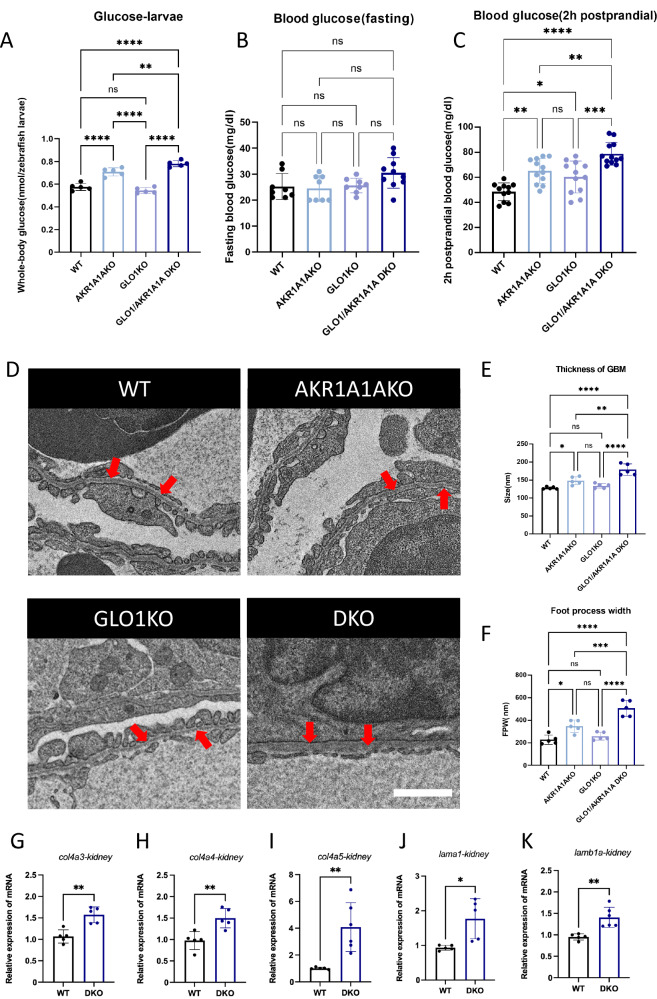
Aggravated hyperglycemia, thickened GBM and podocyte effacement in *Tg(fli1:EGFP)* GLO1/AKR1A1A DKO zebrafish. **A** The whole-body glucose levels in GLO1/AKR1A1A DKO larvae at 96hpf were the highest compared to WT, GLO1KO and AKR1A1AKO larvae. *n* = 5 biological replicates per group. Exact *p* values for each comparation are: WT vs. AKR1A1AKO, WT vs. DKO, AKR1A1AKO vs. GLO1KO, GLO1KO vs. DKO, *p* < 0.0001; AKR1A1AKO vs. DKO, *p* = 0.008. hpf, hours of post fertilization. **B** Fasting blood glucose levels in DKO adult zebrafish at 12mpf were unaltered. *n* = 8/10 biological replicates per group. mpf, months of post fertilization. **C** 2-hour postprandial blood glucose levels in DKO adult zebrafish at 12mpf were further elevated compared to WT, GLO1KO and AKR1A1AKO adult zebrafish. *n* = 11/12 biological replicates per group. Exact *p* values for each comparation are: WT vs. AKR1A1AKO, *p* = 0.0012; WT vs. GLO1KO, *p* = 0.0333; WT vs. DKO, *p* < 0.0001; AKR1A1AKO vs. DKO, *p* = 0.0089; GLO1KO vs. DKO, *p* = 0.0002. **D** Representative electron micrographs of glomeruli from WT, GLO1KO, AKR1A1AKO and DKO adult zebrafish. Red arrows indicated GBM. White scale bar = 1 µm. GBM, glomerular basement membrane. **E** Quantification of GBM thickness in adult zebrafish, showing a significant increase in the DKO group compared to WT, GLO1KO, and AKR1A1AKO groups. *n* = 5 biological replicates per group. Exact *p* values for each comparation are: WT vs. AKR1A1AKO, *p* = 0.0293; WT vs. DKO, GLO1KO vs. DKO, *p* < 0.0001; AKR1A1AKO vs. DKO, *p* = 0.0012. **F** Quantification of FPW in adult zebrafish, showing a significant increase in the DKO group compared to WT, GLO1KO, and AKR1A1AKO groups. *n* = 5 biological replicates per group. Exact *p* values for each comparation are: WT vs. AKR1A1AKO, *p* = 0.0107; WT vs. DKO, *p* < 0.0001; GLO1KO vs. DKO, *p* < 0.0001; AKR1A1AKO vs. DKO, *p* = 0.0009. FPW, foot process width. **G–K** Relative mRNA expression levels of *col4a3*, *col4a4*, *col4a5*, *lama1*, *lamb1a* were upregulated in DKO kidney compared to WT. *n* = 5/6 biological replicates per group. Exact *p* values for each comparation are: col4a3, *p* = 0.0017; col4a4, *p* = 0.0055; col4a5, *p* = 0.0089; lama1, *p* = 0.0310; lamb1a, *p* = 0.0040. col4a3, collagen-type IV, alpha 3; col4a4, collagen-type IV, alpha 4; col4a5, collagen-type IV, alpha 5; lama1, laminin-alpha 1; lamb1a, laminin-beta 1a. mRNA expression levels were quantified by RT-qPCR and normalized to *arnt 2*. arnt2, aryl hydrocarbon receptor nuclear translocator 2. The bars indicate mean ± SD values. Statistical analysis were performed by a two-tailed Student’s *t*-test and one-way ANOVA. ns, not significant, *p < 0.05, **p < 0.01, ***p < 0.001, ****p < 0.0001. Source data are provided as a Source Data file.

To investigate whether the combined loss of GLO1 and AKR1A1A has an influence on glucose dysregulation in zebrafish, we measured whole-body glucose levels in *Tg(fli1:EGFP)* larvae at 96hpf and blood glucose levels in adult zebrafish at 12mpf. Compared to the WT, GLO1KO, and AKR1A1AKO groups, the DKO zebrafish exhibited significant elevations in whole-body glucose and 2h-postprandial blood glucose levels, while fasting blood glucose remained unchanged (Fig. 5A–C). These findings indicated exacerbated impairment of glucose homeostasis in GLO1/AKR1A1A DKO zebrafish.

In addition to assessing glucose homeostasis, we performed histological, ultrastructural and molecular analyses of zebrafish kidneys. Histological analysis using hematoxylin and eosin (HE) staining, periodic acid-Schiff (PAS) staining, and Masson trichrome staining revealed altered glomeruli, including increased cell number and diffuse matrix expansion, as well as increased collagen deposition, in GLO1/AKR1A1A DKO adult kidneys compared to wildtype kidneys (Fig. S6A–C). In addition, we measured the width of the GBM and podocyte foot process in *Tg(fli1:EGFP)* adult zebrafish by electron microscopy (Fig. S1A), as GBM thickening and podocyte effacement are two hallmark pathological features of diabetic nephropathy^40,41^. A significant increase in GBM thickness and podocyte effacement was observed in DKO kidney compared to WT, GLO1KO, and AKR1A1AKO groups (Fig. 5D–F). Afterwards, we performed RT-qPCR to assess genes involved in extracellular matrix (ECM) remodeling - collagen-type IV, alpha 3 (*col4a3*), collagen-type IV, alpha 4 (*col4a4*), collagen-type IV, alpha 5 (*col4a5*), laminin-alpha 1 (*lama1*), laminin-beta 1a (*lamb1a*), stress responses - mitogen-activated protein kinase 8b (*mapk8b*), *atf4a*, *atf4b*, inflammation - colony stimulating factor 1b (*csf1b*), interferon gamma 1 (*ifng1*), and arginine metabolism - *ass1*, *asl*, *arg1*, *odc1* in adult DKO and WT kidney. The results showed that the expression levels of *col4a3*, *col4a4*, *col4a5*, *lama1*, and *lamb1a* were significantly upregulated (Fig. 5G–K). The data align with observations of human kidneys with diabetic nephropathy, in which glomerular basement membrane thickening is primarily driven by increased collagen IV and laminin deposition^42,43^. In addition, the expression levels of *mapk8b*, *atf4a*, and *atf4b*, *csf1b*, *ifng1* and *ass1* were increased, whereas *asl, arg1* and *odc1* showed no significant changes (Fig. S7A–I).

We also evaluated the trunk and hyaloid vasculature in *Tg(fli1:EGFP)* larvae and the adult zebrafish retina (Fig.e S1A). The trunk vasculature showed no differences among the four groups (Fig. S8A, B). With the exception of the previously reported increase in branch points and sprout formation in the hyaloid and retina vasculature of AKR1A1AKO zebrafish^9^, no additional vascular alterations were detected in DKO zebrafish compared to the WT, GLO1KO, and AKR1A1AKO groups (Fig. S8C–F and Fig. S9A–C). To identify the potential antioxidant enzymes in DKO retina, the expression of various *aldhs* and *akrs* was quantified by RT-qPCR. The results showed that aldehyde dehydrogenase 3 family, member A1 (*aldh3a1*), aldehyde dehydrogenase 3 family, member A2a (*aldh3a2a*), aldehyde dehydrogenase 9 family, member A1b (*aldh9a1b*) and aldo-keto reductase family 7, member A3 (*akr7a3*) were upregulated. Aldo-keto reductase family 1, member B1 (aldose reductase), tandem duplicate 1 (*akr1b1.1*) was downregulated, and aldehyde dehydrogenase 3 family, member A2b (*aldh3a2b*), aldehyde dehydrogenase 3 family, member B1 (*aldh3b1*) and aldo-keto reductase family 1, member A1b (*akr1a1b*) were unchanged in DKO eyes compared to WT (Fig. S10A–H). The data helps to explain why the retina is largely unaffected in the DKO model. Together, these findings demonstrated that GLO1/AKR1A1A DKO zebrafish exhibit an organ-specific hallmark of diabetic nephropathy.

### Arginine supplementation attenuated glucose levels in GLO1/AKR1A1A DKO larvae and in larvae co-treated with MG and ACR

**Fig. 6:**
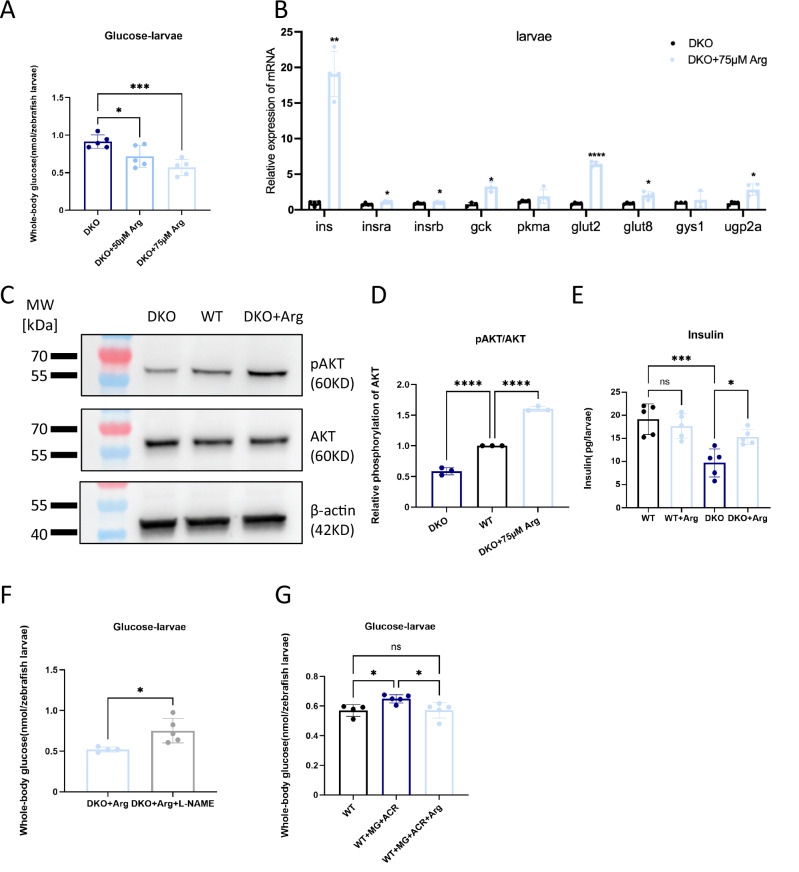
Arginine supplementation attenuated glucose levels in GLO1/AKR1A1A DKO larvae and larvae co-treated with MG and ACR. **A** Arginine supplementation reversed the glucose elevation in GLO1/AKR1A1A DKO larvae. *n* = 5 biological replicates per group. Exact *p* values for each comparation are: DKO vs. DKO + 50 µM Arg, *p* = 0.037; DKO vs. DKO + 75 µM Arg, *p* = 0.001. Arg, L-arginine. **B** Arginine supplementation significantly upregulated the mRNA expression levels of *ins*, *insra*, *insrb*, and genes related to glucose transport, glycolysis and glycogenesis. *n* = 3/4 biological replicates per group. mRNA expression levels were quantified by RT-qPCR and normalized to *arnt 2*. Exact *p* values for each comparation are: ins, *p* = 0.0013; insra, *p* = 0.0172; insrb, *p* = 0.0269; gck, *p* = 0.0200; pkma, *p* = 0.2491; glut2, *p* < 0.0001; glut8, *p* = 0.0189; gys1, *p* = 0.6129; ugp2a, *p* = 0.0164. ins, insulin; insra, insulin receptor a; insrb, insulin receptor b; gck, glucokinase; pkmb, pyruvate kinase M1/2b; glut2, glucose transporter 2; glut8, glucose transporter 8; gys1, glycogen synthase 1; ugp2a, UDP-glucose pyrophosphorylase 2a; arnt2, aryl hydrocarbon receptor nuclear translocator 2. **C**,**D** Representative Western blotting images (**C**) and quantification (**D**) demonstrated decreased phosphorylated AKT level in DKO larvae, with arginine supplementation restoring AKT phosphorylation at 96hpf. *n* = 3 biological replicates per group. Exact *p* values for each comparation are: WT vs. DKO, WT vs. DKO + 75 µM Arg, *p* < 0.0001. hpf, hours of post fertilization. **E** Reduced insulin in DKO larvae at 96hpf which could be rescued after arginine supplementation. The samples were analyzed by an ELISA. *n* = 5 biological replicates per group. Exact *p* values for each comparation are: WT vs. DKO, *p* = 0.0003; DKO vs. DKO+Arg, *p* = 0.0248. **F** L-NAME increased glucose levels in GLO1/AKR1A1A DKO larvae treated with arginine through inhibition of NOS. *n* = 4/5 biological replicates per group. Exact *p* values for each comparation are: *p* = 0.0159. L-NAME, N(ω)-nitro-L-arginine methyl ester; NOS, nitric oxide synthase. **G** Arginine supplementation lowered the hyperglycemia induced by the co-treatment with MG and ACR. *n* = 4/5 biological replicates per group. Exact *p* values for each comparation are: WT vs. WT + MG + ACR, p = 0.0407; WT + MG + ACR vs. WT + MG + ACR+Arg, *p* = 0.0336. The bars indicate mean ± SD values. Statistical analysis were performed by one-way ANOVA and a two-tailed Student’s *t*-test. ns, not significant, *p < 0.05, **p < 0.01, ***p < 0.001, ****p < 0.0001. Source data are provided as a Source Data file.

To further clarify the role of arginine in glucose metabolism, the whole-body glucose levels, transcript expression, and activation and expression of protein kinase AKT (pAKT/AKT) in DKO larvae with and without L-arginine (Arg) supplementation were quantified using ELISA, RT-qPCR and Western blotting. First, the safe dose of Arg was determined in larvae exposed to 0–5 mM Arg from 48 to 96 hpf (Fig. S11A). Concentrations below 100 µM were found to be well tolerated (Fig. S11B)^44^. Next, DKO larvae treated with 50 or 75 µM Arg showed significantly reduced glucose levels compared to untreated controls (Fig. 6A), and arginine supplementation in DKO larvae normalized arginine levels to the physiological concentrations comparable to those in WT larvae (Fig. S11C). Sodium nitroprusside (SNP), a NO donor, has the same effect to lower the glucose levels in DKO larvae (Fig. S12A–C). To investigate the mechanism underlying the glucose-lowering effect of Arg, RT-qPCR analysis showed that Arg supplementation markedly upregulated the expression of *ins*, *insra*, *insrb*, *gck*, *glut2*, *glut8*, and *ugp2a* (Fig. 6B). Western blotting further demonstrated a decrease in phosphorylated AKT levels in DKO larvae, which was restored by Arg treatment (Fig. 6C, D), and after arginine supplementation, the decreased insulin protein levels in DKO larvae were also restored (Fig. 6E). To further assess whether NO contributes to the glucose-lowering effect, we treated DKO larvae supplemented with Arg using N(ω)-nitro-L-arginine methyl ester (L-NAME), a non-selective nitric oxide synthase (NOS) inhibitor that suppresses NO production^45^. The safe dose of L-NAME was determined to be below 1 mM (Fig. S13A–C)^46^. The result showed that co-treatment with L-NAME eliminated the glucose-lowering effect of Arg (Fig. 6F), but the L-NAME treatment alone didn’t change glucose concentrations in WT and DKO groups (Fig. S13D, E). In addition, Arg supplementation could lower the hyperglycemia induced by the co-treatment with MG and ACR (Fig. 6G). Thus, these findings showed that Arg supplementation attenuates glucose levels in GLO1/AKR1A1A DKO larvae and larvae co-treated with MG and ACR by enhancing insulin signaling, glycolysis and glycogenesis.

### Arginine supplementation rescued pdx1 morpholino-induced hyperglycemic pronephros alterations

**Fig. 7:**
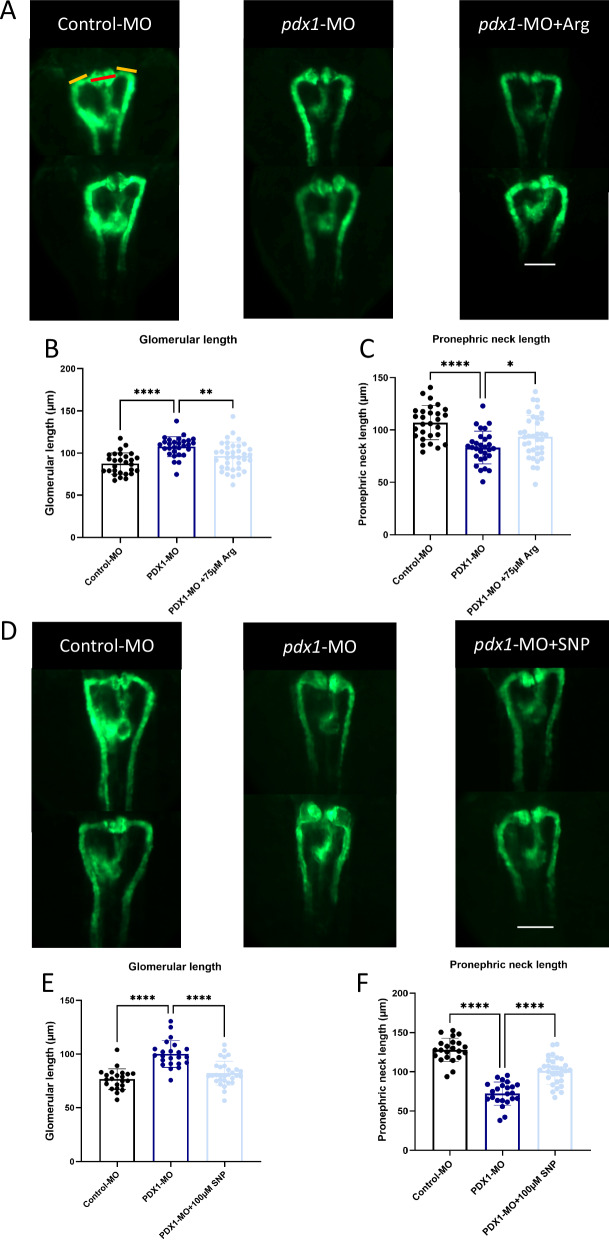
Arginine supplementation rescued *pdx1* morpholino-induced hyperglycemic pronephros alterations. **A** Representative confocal images of the pronephros morphology in control and pdx1 morphants *Tg(wt1b:EGFP)* zebrafish larvae at 48 hpf, with or without arginine supplementation. Red line indicates glomerular length, yellow line indicates pronephric neck length. White scale bar = 100 µm. hpf, hours of post fertilization; MO; morphants; pdx1, pancreatic and duodenal homeobox 1. **B** Quantification of glomerular length in larvae showed a significant increase in pdx1 morphants, which is reduced by arginine supplementation, compared to control group. *n* = 28/36 biological replicates per group. Exact *p* values for each comparation are: PDX1-MO vs. Control-MO, p < 0.0001; PDX1-MO vs. PDX1-MO + 75 µM Arg, *p* = 0.0039. Arg, L-arginine. **C** Quantification of pronephric neck length in larvae showed a significant decrease in pdx1 morphants, which is restored by arginine supplementation, compared to control group. *n* = 28/36 biological replicates per group. Exact *p* values for each comparation are: PDX1-MO vs. Control-MO, *p* < 0.0001; PDX1-MO vs. PDX1-MO + 75 µM Arg, *p* = 0.0439. **D** Representative confocal images of the pronephros morphology in control and pdx1 morphants *Tg(wt1b:EGFP)* zebrafish larvae at 48 hpf, with or without SNP supplementation. White scale bar = 100 µm. SNP, sodium nitroprusside. **E** Quantification of glomerular length in larvae showed a significant increase in pdx1 morphants, which is reduced by SNP supplementation, compared to control group. *n* = 22/30 biological replicates per group. Exact *p* values for each comparation are: PDX1-MO vs. Control-MO, *p* < 0.0001; PDX1-MO vs. PDX1-MO + 100 µM SNP *p* < 0.0001. **F** Quantification of pronephric neck length in larvae showed a significant decrease in pdx1 morphants, which is restored by SNP supplementation, compared to control group. *n* = 22/30 biological replicates per group. Exact *p* values for each comparation are: PDX1-MO vs. Control-MO, *p* < 0.0001; PDX1-MO vs. PDX1-MO + 100 µM SNP, *p* < 0.0001. The bars indicate mean ± SD values. Statistical analysis was performed by one-way ANOVA. *p < 0.05, **p < 0.01, ****p < 0.0001. Source data are provided as a Source Data file.

To further clarify the contribution of Arg in hyperglycemia-associated kidney alterations in DKO mutants, we analyzed pronephros morphology with and without Arg supplementation, and with and without SNP in hyperglycemic *pdx1* morphants using the *Tg*(*wt1b*:*EGFP*) line that specifically labels the early pronephros with EGFP^47,48^. A morphological assessment revealed that *pdx1* morphants exhibited a pronounced enlargement of the glomeruli, reflected by a significant increase in glomerular length, along with a marked reduction in pronephric neck length. These features are consistent with those previously described for hyperglycemic renal impairment^47,48^. Remarkably, administration of Arg effectively ameliorated these abnormalities, as evidenced by reducing glomerular length and elevating pronephric neck length toward the normal characteristic (Fig. 7A–C). In addition, SNP supplementation exerts the same rescue effect on pronephros alterations in hyperglycemic zebrafish (Fig. 7D–F). These findings underscored the protective role of Arg and SNP in counteracting hyperglycemia-induced kidney alterations.

## Discussion

The present study investigated zebrafish mutants deficient in either GLO1 or AKR1A1A, or both (GLO1/AKR1A1A). Dual loss of GLO1 and AKR1A1A caused a pronounced accumulation of MG and ACR, impaired arginine homeostasis and insulin signaling, hyperglycemia, as well as GBM thickening and podocyte effacement, which are characteristic features of diabetic kidney disease (DKD). Notably, supplementation with exogenous arginine normalized glucose levels and restored renal morphology, highlighting the coordinated role of GLO1 and AKR1A1A in detoxifying RCS, maintaining arginine metabolism, glucose and renal homeostasis.

Clinical studies have also shown that circulating levels of MG and glyoxal are significantly elevated in patients with types 1 and 2 diabetes compared to healthy individuals^49^. Similarly, markedly increased plasma concentrations of both free and protein-conjugated ACR have been documented in patients with renal failure^50^. Consistently, in a dietary context, high-fat diet–fed mice displayed increased levels of both MG and ACR^51^. Our findings extend these clinical and experimental observations by providing direct genetic evidence that GLO1 and AKR1A1A act cooperatively to maintain carbonyl stress and arginine homeostasis. In GLO1/AKR1A1A DKO zebrafish, the concurrent loss of both enzymes led to accumulation of MG and ACR, accompanied by activation of stress-responsive pathway, impaired arginine homeostasis and insulin signaling, and renal abnormalities. These results suggest that MG and ACR cooperate to amplify carbonyl stress by modifying arginine residues, thereby disrupting arginine-dependent signaling and metabolic pathways. Together, our results define a synergistic regulatory axis between GLO1 and AKR1A1A that links RCS detoxification to arginine metabolism, providing a molecular explanation for how carbonyl stress contributes to glucose and renal dysregulation.

Our findings demonstrate that arginine deficiency impairs insulin signaling and promotes glucose dysregulation, thereby contributing to renal injury. Supplementing with L-arginine in zebrafish larvae was found to significantly increase insulin-related gene expression, insulin levels, and AKT phosphorylation, suggesting improved insulin signaling lowers the glucose levels and complications. Extensive clinical and preclinical studies have been demonstrated beneficial effects of L-arginine supplementation on endothelial function, *β*-cell function, insulin sensitivity and oxidative stress attenuation^24,52^. For example, Dashtabi et al. reported that individuals with obesity receiving 3 or 6 g of L-arginine daily for eight weeks experienced significant reductions in fasting blood glucose (FBG) and glycated hemoglobin (HbA1c) levels^53^. Similarly, another clinical trial in individuals with obesity revealed that long-term administration of 9 g of L-arginine per day for six months markedly lowered both FBG and insulin concentrations^54^. Furthermore, a long-term interventional study and its extension suggested that the administration of L-arginine could delay the onset of T2DM in individuals with impaired glucose tolerance (IGT) and metabolic syndrome (MS)^55,56^. Of note, despite impaired postprandial insulin signaling, as indicated by the reduced AKT phosphorylation in the livers and muscles of DKO zebrafish, our data also revealed a decrease in arginine levels and altered arginine metabolism in the liver but not in the muscle, where the arginine biosynthetic enzymes operate and is therefore of particular importance for arginine metabolism^57^. These findings further support the hypothesis that hepatic arginine metabolism plays a central role in coordinating systemic metabolic responses across peripheral tissues^58^. Future preclinical and clinical studies linking tissue-specific arginine metabolic reprogramming to systemic insulin regulation will be necessary. Moreover, several studies indicate that arginine supplementation reduces microalbuminuria, slows DKD progression, enhances glomerular filtration rate (GFR), and mitigates GBM thickening^59–61^. Additionally, arginine modulates glucose metabolism primarily through the arginine–NO signaling pathway, which improves insulin sensitivity^60^. Consistently, non-selective inhibition of NOS with L-NAME in our study decreased NO bioavailability and aggravated hyperglycemia, highlighting the pivotal role of the L-arginine/NO axis in metabolic regulation^14,62,63^.

An intriguing observation was the absence of retinal vascular alterations in DKO zebrafish, despite elevated carbonyls stress, concurrent presence of hyperglycemia and diabetic nephropathy. This suggests that organ-specific susceptibility to diabetic complications is influenced by distinct RCS profile and corresponding organ-specific antioxidant rather than hyperglycemia alone, in line with prior studies^64,65^. GLO1KO zebrafish with moderate MG elevation displayed no morphological alterations to the retinal vessels or kidneys. In contrast, AKR1A1AKO zebrafish with elevated ACR, but normal MG levels, exhibited retinal vessel and kidney morphological abnormalities. However, GLO1/AKR1A1A DKO zebrafish, with concurrent MG and ACR accumulation, developed pronounced renal pathology without retinal blood vessel involvement. The study therefore emphasizes the need to quantify multiple RCS profiles as noninvasive parameters in diabetic patients, as these may be predictive of the development of organ-specific complications in diabetes such as nephropathy or retinopathy. In addition, our findings demonstrated that combined GLO1/AKR1A1A deficiency activates compensatory or protective mechanisms specifically in the retina, through the upregulation of antioxidant enzyme expression. However, further studies are necessary to identify the full carbonyl detoxification network in the eye.

In summary, our data demonstrates that the dual loss of GLO1 and AKR1A1A cooperatively drives the accumulation of reactive carbonyl species (MG and ACR), impairs arginine homeostasis and insulin signaling, leading to hyperglycemia and hallmarks of diabetic nephropathy. Notably, arginine supplementation effectively reverses these alterations, underscoring the synergistic role of GLO1 and AKR1A1A in keeping arginine homeostasis and the potential as a therapeutic target for diabetes, diabetic nephropathy and other chronic diseases associated with carbonyl stress. Together with our previous research, the present study provides insight into how distinct RCS profiles, shaped by synergistic function of GLO1 and AKR1A1A, contribute to organ-specific complications, offering a mechanistic and metabolic basis for individualized therapeutic strategies across a spectrum of metabolic disorders.

## Methods

### Zebrafish husbandry

All experimental procedures on animals were approved by Medical Faculty Mannheim (license number: I-21/18 and I-21/19), and carried out according to the approved guidelines. The study used the zebrafish strains *Tg(fli1:EGFP)* and *Tg(wt1b:EGFP)* to visualize the vasculature and pronephros, respectively. The fish were maintained under standardized laboratory conditions and the protocols were followed as previously established^66^. Embryos and larvae up to 5dpf were cultured in egg water at 28.5 °C, with the medium renewed daily. Adult zebrafish were housed under a static dark–light cycle and were fed freshly hatched Artemia Salina in the morning and flake food in the afternoon. In this study, sex was not considered at the larval developmental stage because it is not yet differentiated. At the adult stage, only male mice were used in the experiments to minimize hormonal variability.

### Mutant generation

The GLO1KO and AKR1A1AKO zebrafish were individually established using CRISPR/Cas9 genome-editing system, following previously published protocols^9,33^. To obtain the GLO1/AKR1A1A DKO line, GLO1KO zebrafish were outcrossed with AKR1A1AKO zebrafish to generate DHET. These DHET zebrafish were subsequently intercrossed to produce DKO offspring, which were then used for maintenance and experimental analyses. For genotyping, genomic DNA was extracted, and the corresponding PCR amplicons were subjected to Sanger sequencing for verification (Supplementary Table 1).

### Glucose measurements in larvae and adult zebrafish

For larval glucose quantification, 20 larvae were collected at 96hpf and immediately frozen in liquid nitrogen. The samples were homogenized in assay buffer with a 3 mm bead using a tissue lyser. The whole-body glucose concentrations in larvae were determined using a commercial Glucose Assay Kit (MAK263, Sigma-Aldrich) according to the manufacturer’s instructions, and the absorbance at 570 nm was measured with a microplate reader (Tecan Infinite M200).

For adult zebrafish blood glucose measurement, male zebrafish were individually placed in boxes and fasted overnight. On the following day, adult zebrafish were either maintained under fasting conditions or fed 0.5 g of flake food for 1 h, followed by 1 h in fresh water to assess postprandial glucose levels. Zebrafish were then euthanized in ice water for 2 min, and blood samples were collected from the caudal vessels for glucose measurement using a glucometer. Afterward, the body weight and length were recorded. Livers and muscles were dissected, weighed, immediately frozen in liquid nitrogen, and stored at –80 °C until further analysis. Kidneys and eyes were dissected for GBM thickness, FPW and retinal vasculature analysis.

### Electron microscopy (EM)

Kidneys were dissected and immediately immersed in 3% glutaraldehyde prepared in 0.1 M cacodylate buffer (pH 7.4) for fixation at room temperature for a minimum of 2 h. The fixed tissues were then cut into small blocks of approximately 1 mm³. After fixation, the kidney samples were rinsed several times in cacodylate buffer and post-fixed with 1% osmium tetroxide for 1 h at 4 °C. Following post-fixation, samples were washed with distilled water, dehydrated through a graded ethanol series, and subsequently transferred to propylene oxide before embedding in epoxy resin (Glycid Ether 100).

For light microscopy, semithin sections (1 µm) were stained with methylene blue and observed under a light microscope (Olympus). For ultrastructural analysis, ultrathin sections (60–80 nm) were contrasted with uranyl acetate and lead citrate and examined using a JEM-1400 transmission electron microscope equipped with a 2 K TVIPS CCD camera (TemCam F216).

### Injection of pdx1morpholino into zebrafish embryos

The following morpholino oligonucleotides were used: SB-*pdx1*-Mo (5′-GATAGTAATGCTCTTCCCGATTCAT-3′) and control-Mo (5′-CCTCTTACCTCAGTTACAATTTATA-3′)^67^. Both *Pdx1* and control morpholinos were diluted to a working concentration of 6 μg/μl in 0.1 M KCl. 1 nL of the morpholino solution was microinjected into the yolk sac of *Tg*(*wt1b*:*EGFP*) embryos at the one-cell stage^47^.

### Microscopy and analysis of pronephros structure

To evaluate pronephric morphology in zebrafish embryos, *Tg*(*wt1b*:*EGFP*) embryos from 24 hpf were kept in egg water with 0.003% 1-phenyl-2-thiourea (Sigma) to prevent pigmentation. At 48 hpf, embryos were anesthetized with 0.003% tricaine and positioned dorsally in 1% low-melting-point agarose (Promega) prepared in egg water. Fluorescent images were acquired using a Leica MZ10 F stereo microscope equipped with a DFC420 C digital camera. Morphological alterations of the pronephros were quantified by measuring the glomerular length and pronephric neck length with the Leica LAS software (version 4.8)^47^.

### Microscopy and analysis of vascular alterations in larvae and adults

For trunk vasculature imaging, *Tg*(*fli*:*EGFP*) zebrafish larvae were treated with 0.003% 1-phenyl-2-thiourea (Sigma) from 24 hpf to inhibit pigmentation. At 96 hpf, larvae were anesthetized with 0.003% tricaine, positioned laterally in 96-well plates, and imaged using a Leica DM6000B confocal microscope with a TCS SP5 DS scanner (600 Hz, 1024 × 512 pixels, 1 µm z-steps). Intersegmental vessels (ISVs) between the 6th and 22nd pairs were analyzed, excluding the first five and the dorsal longitudinal anastomotic vessel (DLAV). Newly formed blood vessels between ISVs were defined as “hyperbranches”.

For hyaloid vasculature imaging, *Tg*(*fli*:*EGFP*) zebrafish larvae at 120 hpf were anesthetized, fixed in 4% paraformaldehyde (PFA) overnight at 4 °C, washed in PBS, and digested with 0.25% Trypsin/EDTA buffered with 1.5 M Tris (pH 7.8) for 80 min at 37 °C. After washing, samples were stored in PBS until dissection. The hyaloid vasculature was isolated according to Jung’s protocol^68^ and imaged using the same confocal setup (20×0.7 objective, 1.5 µm z-steps).

For adult retinal vasculature, procedures followed Wiggenhauser’s method^69^. PFA-fixed heads were placed on an ice-cooled agarose platform, and eyes were dissected, washed, and mounted in imaging medium. Confocal imaging was performed as above. Vascular branch points and sprouts were quantified within 350 × 350 µm² regions using ImageJ and GIMP software.

### MG, 3-DG and glyoxal measurements

50 larvae per sample at 96hpf were collected and flash frozen in liquid nitrogen. MG, glyoxal and 3-DG were measured as previously described^33,36^.

### Acrolein measurement in larvae

50 larvae per sample at 96hpf were collected and snap frozen. Protein-bound ACR was determined according to manufacturer’s instruction (Acrolein ELISA Kit, MBS7213206, MyBioSource Inc)^9^.

### 4-HNE measurements in larvae

Fifty larvae per sample at 96hpf were collected and snap frozen. 4-HNE amount was determined according to the manufacturer’s instruction (4-Hydroxynonenal ELISA Kit, ab287803, Abcam)^70^.

### Acetaldehyde measurements

50 larvae per sample at 96hpf were collected and flash frozen in liquid nitrogen. AA was measured in a LC-MS/MS experimental setup according to an adjusted protocol of Jeon et al.^71^.

### Lipid peroxidation assay

The levels of thiobarbituric acid reactive substances (TBARS), mainly malondialdehyde (MDA), were determined in 96 hpf larvae using a lipid peroxidation assay kit (ab118970, Abcam)^36^.

### AGEs measurements

50 larvae per sample at 96hpf were collected and flash frozen in liquid nitrogen. AGEs, including protein-bound and protein-free MG-H1 were quantified as previously described^72^.

### Metabolomic analysis

Eighty zebrafish larvae at 96 hpf per measurement were snap frozen in liquid nitrogen. Targeted primary metabolites were measured as previously described in cooperation with the Metabolomics Core Technology Platform (MCTP) of the Center for Organismal Studies (COS) of Heidelberg University via gas chromatographymass spectrometry (GC/MS)^73^. GC/MS data was normalized to Ribitol as internal standard and converted to signal intensity per sample amount. MetaboAnalyst 5.0 (www.metaboanalyst.ca) served as the primary online tool for pinpointing the most notably altered metabolites and conducting enrichment analysis. Differential metabolites were screened by fold change analysis with a threshold of 2.0. Advanced analysis related to function or pathway was done based on the KEGG database according to the protocol^74^. The metabolomic data including the assay parameters are available on https://www.ebi.ac.uk/metabolights/MTBLS14089. Data acquisition and processing were performed using Empower3 software (version 3.7.0, Waters).

### Determination of amino acids

50 zebrafish larvae at 96 hpf per measurement were snap frozen in liquid nitrogen. Free amino acids were extracted from 50 larvae with 0.3 ml of 0.1 M HCl containing 10 μM Norleucine as an internal standard in an ultrasonic ice-bath for 10 min. The lysates were centrifuged at 14,000 x g for 5 min at 4 °C, and the supernatants were used for derivatization. Derivatization and separation of amino acids was performed based on the Waters AccQ-Tag system, followed by UPLC/MS analysis as described by Weger et al.^75^. Lastly, amino acids were quantified based on calibration curves generated from amino acid standards.

### Nitrite plus nitrate measurement

40 zebrafish larvae at 96 hpf per measurement were snap frozen in liquid nitrogen. The samples were homogenized in 1X PBS using a tissue lyser. The nitrite and nitrate concentrations in larvae were determined using a Nitrite/Nitrate Assay Kit (23479, Sigma-Aldrich) according to the manufacturer’s instructions, and the absorbance at 540 nm was measured with a microplate reader (Tecan Infinite M200).

### Insulin measurement

20 zebrafish larvae with and without arginine supplementation at 96 hpf per measurement were snap frozen in liquid nitrogen. The samples were homogenized in NP-40 lysis buffer using a tissue lyser. The insulin concentrations were detected using a Zebrafish INS (Insulin) ELISA Kit (ELK0938, ELK Biotechnology) according to the manufacturer’s instructions. Lastly, the microplate reader was applied at 450 nm immediately.

### Histological analysis of adult kidney

The zebrafish were prepared and euthanized. After overnight fixation in 10 % buffered formalin solution, specimens of the kidney were routinely dehydrated, embedded in paraffin, and cut into 4μm-thick sections. HE staining, PAS reaction and Masson Trichrome were done using standard protocols^73^.

### Western blotting

For Western blotting, larvae, liver and muscle were collected and treated on ice with 2 mM Natrium-Vanadate in 1× PBS for 10 min to inhibit phosphatase activity. Subsequently, the samples were homogenized and lysed in NP-40 lysis buffer (50 mM Tris-HCl, pH 7.4, 150 mM NaCl, 1% NP-40, 10 mM EDTA, 10% glycerol, and protease inhibitors) for 30 min with gentle shaking on ice. The lysates were mixed with 5× Laemmli buffer and boiled at 95 °C for 5 min. Proteins were separated by SDS–PAGE and transferred onto 0.2 μm nitrocellulose membranes (Amersham). After blocking with 5% BSA, the membranes were incubated overnight with primary antibodies (1:1000; anti-β-actin, Santa Cruz Biotechnology, sc-47778; AKT, CST 9272S; phospho-AKT, CST 4060 P), followed by incubation with HRP-conjugated secondary antibodies (1:1000; rabbit anti-mouse, Dako P0260 for β-actin; goat anti-rabbit, Dako P0448 for AKT and p-AKT). Protein bands were visualized using Western Lightning Plus ECL reagents (PerkinElmer) and imaged with a Fusion Solo S system (Vilber).

### RT-qPCR

20 larvae at 96hpf were collected and snap frozen. Total RNA was isolated using the RNeasy Mini Kit (Qiagen) according to the manufacturer’s instructions. First Strand cDNA was synthesized with the Maxima First Strand cDNA Synthesis Kit (Thermo Fisher Scientific). Primers were designed using the NCBI primer blast tool and listed in Supplementary Table 1. RT-qPCR was performed using the Power SYBR™ Green PCR Master Mix (Thermo Fisher Scientific) on the QuantStudio 3 or 5 Real-Time PCR System (Thermo Fisher Scientific).

### RNA sequence analysis

Total RNA was extracted from 96 hpf larvae of WT, GLO1KO, AKR1A1AKO, and GLO1/AKR1A1A DKO zebrafish. Sequencing libraries were prepared and processed on the DNBseq platform (BGI, China). Low-quality and adapter-contaminated reads were filtered using SOAPnuke, and the remaining reads were aligned to the zebrafish reference genome (GRCz11) with HISAT2 (v2.1.1). Gene-level quantification was performed with FeatureCounts (v2.0.3), and normalized to counts per million (CPM) using edgeR (v3.40.2) in R (v4.2.3). Differential expression analysis was carried out with limma (v3.54.2), applying an adjusted p < 0.05 and a fold change > 1.5 as significance thresholds. Pathway activity variations across groups were evaluated by GSVA (v1.46.0) using KEGG gene sets (c2.cp.kegg.v7.1) from MSigDB. Results were visualized with ggplot2 (v3.5.1) as volcano plots, heatmaps, and bar graphs. The RNA-Seq datasets are available on https://www.ncbi.nlm.nih.gov/geo/query/acc.cgi?acc=GSE316100.

### Pharmacological treatment of zebrafish embryos / larvae

Approximately 20 fertilized eggs were incubated in 6-well plates containing 5 mL of treatment solution, which was replaced daily. The solutions consisted of egg water supplemented with 0-5mM L-arginine (A8094, Sigma-Aldrich), 0-5 mM SNP (71778, Sigma-Aldrich), 0-1mM L-NAME (N5751, Sigma-Aldrich), 0-500 µM MG (M0252, Sigma-Aldrich), 10 µM acrolein (CSV-S-11030F1, Biozol) and 15 mM Carnosine (C9625; Sigma-Aldrich). Treatments began at 3 hpf and continued until 96hpf. Chorions were carefully removed at 24 hpf using fine forceps.

### Statistical analysis

Each experiment was repeated at least three independent biological replicates. Data are expressed as mean ± standard deviation (SD). All data were analyzed and displayed using GraphPad Prism software (v10.2.3). Statistical comparisons between two groups were performed using a two-sided Student’s *t*-test, whereas comparisons among multiple groups were conducted using one-way ANOVA. Statistical significance was defined as *p* < 0.05, with the following notation: *p* < 0.05, *p < 0.01, **p < 0.001, ***p < 0.0001.

### Reporting summary

Further information on research design is available in the Nature Portfolio Reporting Summary linked to this article.

## Supplementary information

Supplementary Information

Reporting Summary

Transparent Peer Review file

## Source data

Source Data

## Data Availability

The RNA-Seq data generated in this study have been deposited in the GEO (Gene Expression Omnibus, NIH) database under accession number GSE316100. The metabolomics data generated in this study have been deposited in MetaboLights database under accession number MTBLS14089. Other supporting data are presented within the Supplementary Materials. Source data are provided with this paper.

## Code availability

The RNA-seq analysis code has been deposited in a public GitHub repository and is available at: https://github.com/haolihaolihaoli/zebrafish-transcriptomics-akr1a1a-glo1. The repository includes the R script used for the transcriptomic analysis.
