# Supplementary material for: The accumulation of methylglyoxal and acrolein impairs arginine homeostasis causing hyperglycemia and renal abnormalities in male zebrafish
Source: Nat Commun. 2026 Jul 28;17:7565. doi: 10.1038/s41467-026-76082-6 (PMC13416155; doi:10.1038/s41467-026-76082-6)
Supplement: Supplementary file 1 — Supplementary Information [file 41467_2026_76082_MOESM1_ESM.pdf]

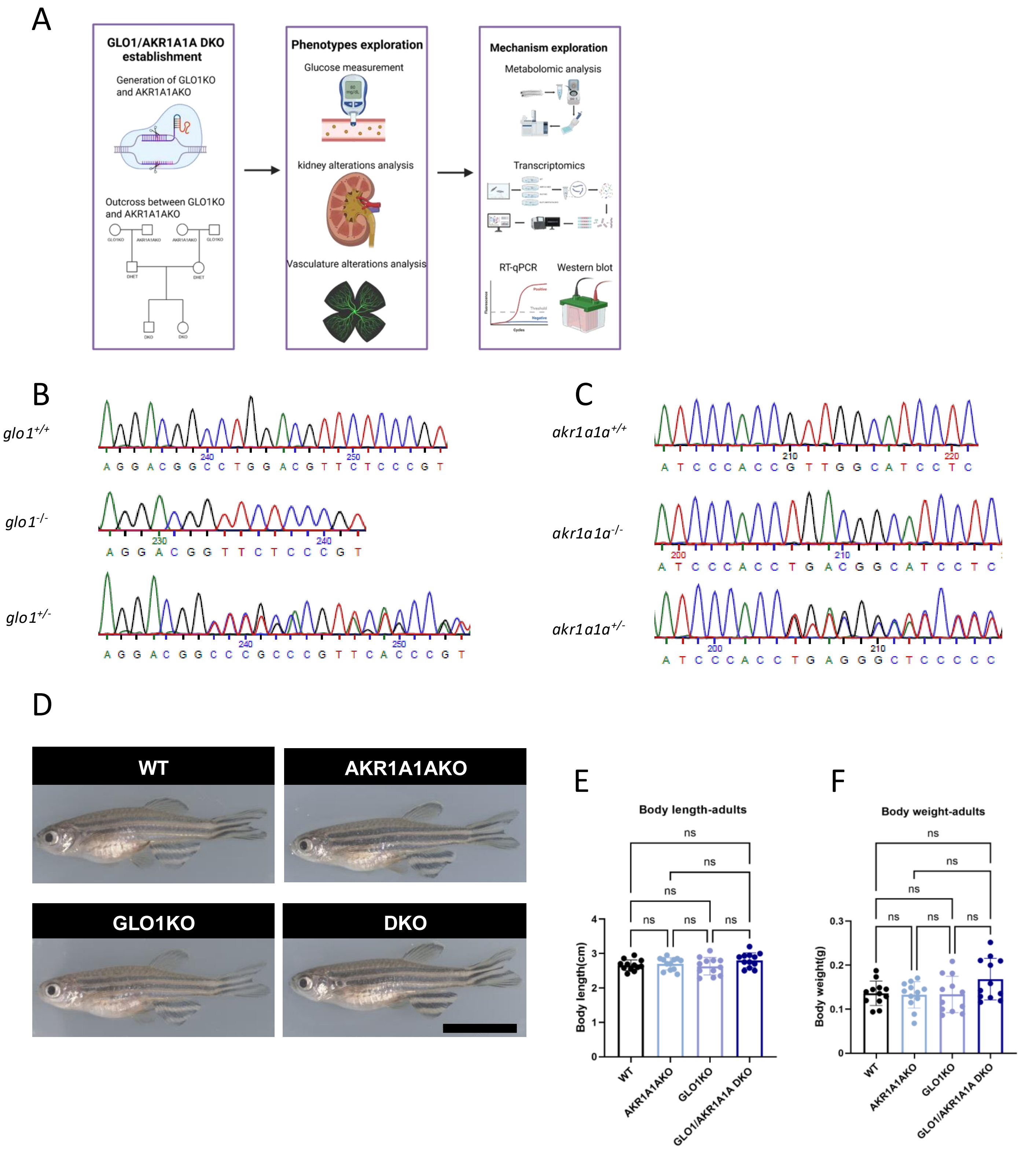


**Suppl. Figure 1. Generation and validation of GLO1/AKR1A1A DKO zebrafish.** (A) Workflow of this study. Created in BioRender. Bennewitz, K. (2026) https://BioRender.com/tnrq6kr. (B-C). Chromatograms of the different mutants. (D) Microscopic images showed unchanged morphology among WT, GLO1KO, AKR1A1AKO and GLO1/AKR1A1A DKO adult zebrafish at 12mpf. Black scale bar = 1cm. mpf, months of post fertilization. (E-F) Body length and body weight of adult zebrafish were unchanged among WT, GLO1KO, AKR1A1AKO and DKO groups. n = 12 biological replicates per group. The bars indicate mean ± SD values. Statistical analysis was performed by one-way ANOVA. ns, not significant.

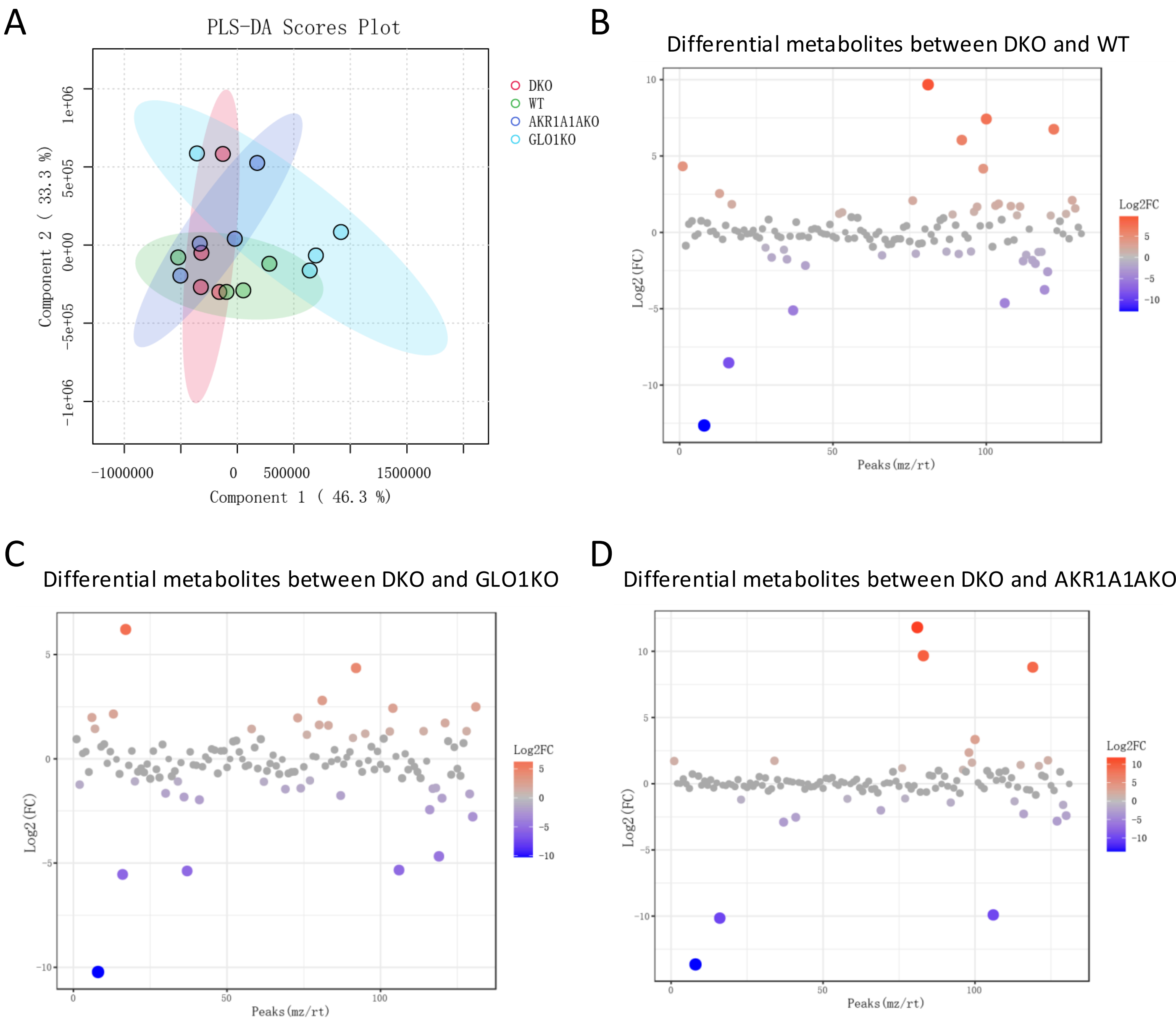


**Suppl. Figure 2. Altered metabolites in GLO1/AKR1A1A DKO zebrafish larvae compared to WT, AKR1A1AKO and GLO1KO larvae.** (A) PLS-DA scores plot revealed distinct clustering of the larvae into different groups. n = 4 biological replicates per group. (B-D) Fold change analysis identified pairwise differential metabolites in the comparisons of DKO vs. WT (B), DKO vs. GLO1KO (C), and DKO vs. AKR1A1AKO (D).

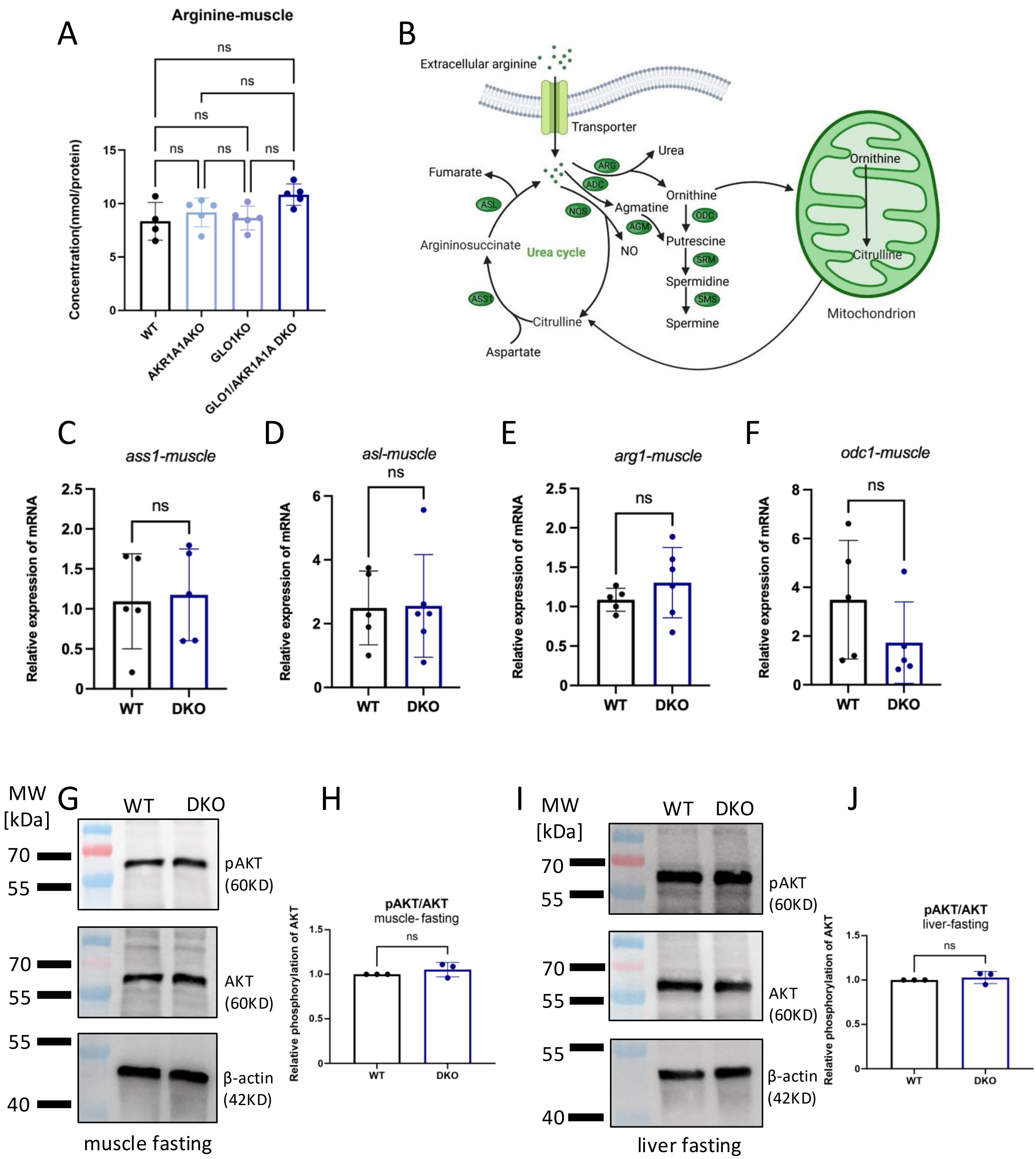


**Suppl. Figure 3. Unchanged arginine metabolism and unchanged fasting AKT phosphorylation in GLO1/AKR1A1A DKO adult muscle.** (A) Arginine levels were unchanged in DKO muscle tissues compared to WT, GLO1KO and AKR1A1AKO groups. n = 4/5 biological replicates per group. (B) The diagram of biochemical pathways in arginine metabolism. Created in BioRender. Bennewitz, K. (2026) *https://BioRender.com/sib8ggw.* (C-F) Relative mRNA expression levels of genes involved in arginine synthesis and catabolism in DKO muscle remain unchanged. n = 5/6 biological replicates per group. mRNA expression levels were quantified by RT-qPCR and normalized to *arnt* 2. ass1, argininosuccinate synthase 1; asl, argininosuccinate lyase; arg1, arginase 1; odc1, ornithine decarboxylase 1; arnt2, aryl hydrocarbon receptor nuclear translocator 2. (G-J) Unchanged AKT phosphorylation in muscle and liver of DKO zebrafish under fasting conditions. n = 3 biological replicates per group. The bars indicate mean ± SD values. Statistical analysis were performed by one-way ANOVA and Student's t-test. ns, not significant.

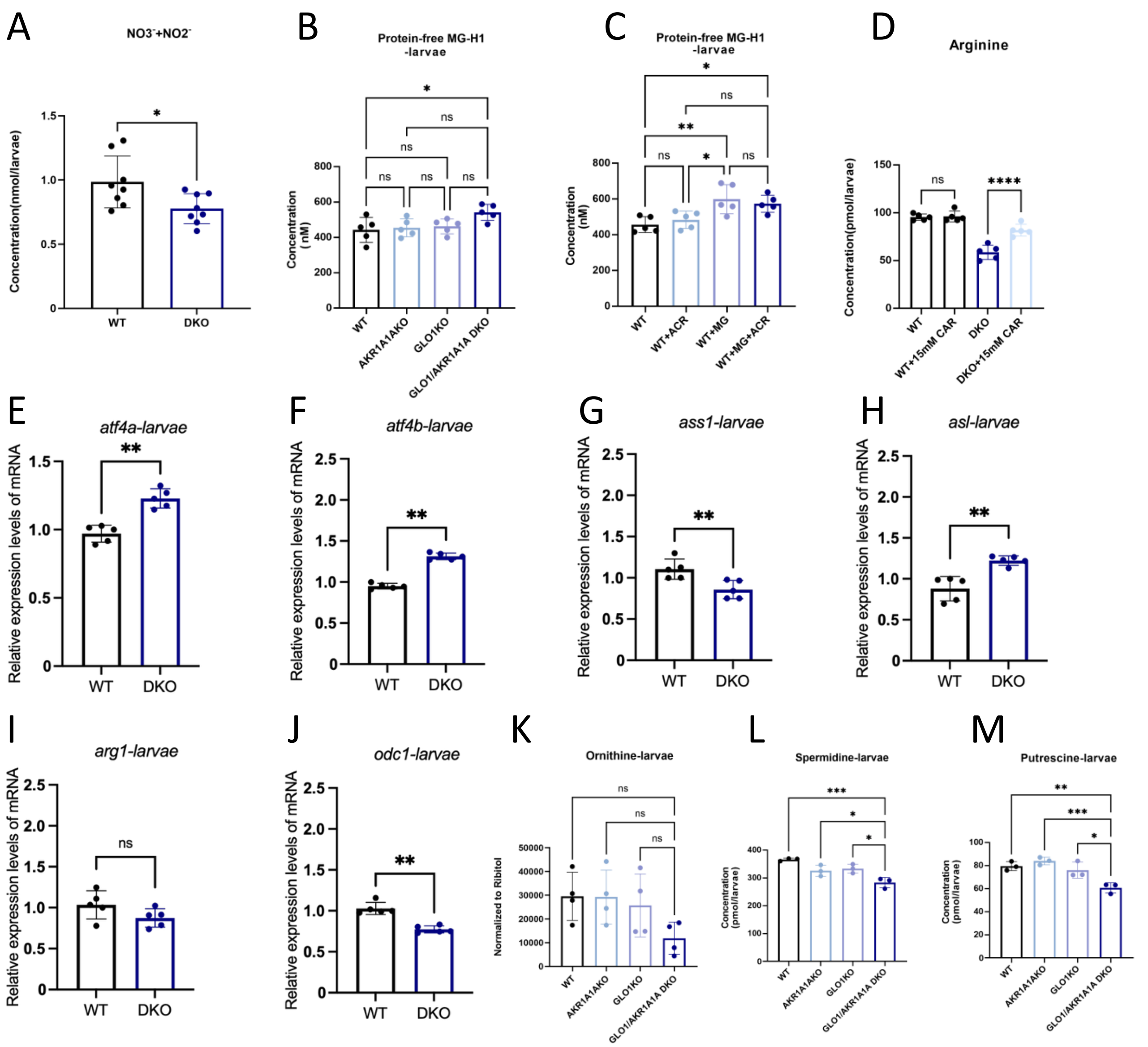


**Suppl. Figure 4. Protein-free MG-H1 induced by GLO1/AKR1A1A deficiency and MG+ACR co-treatment.** (A) Combined nitrite and nitrate concentrations were decreased in DKO larvae at 96 hpf compared to WT. n = 8 biological replicates per group. hpf, hours of post fertilization. (B) Protein-free MG-H1 levels in GLO1/AKR1A1A DKO larvae were elevated compared to WT group. n = 5 biological replicates per group. (C) Protein-free MG-H1 levels were significantly increased in WT larvae treated with 500μM MG alone or with 500μM MG plus 10μM ACR compared to WT larvae, whereas no change was observed in larvae treated with 10μM ACR alone. n = 5 biological replicates per group. MG, methylglyoxal; ACR, acrolein; MG-H1, methylglyoxal-derived hydroimidazolone 1. (D) Carnosine treatment prevented the decrease in arginine levels in DKO mutants. n = 5 biological replicates per group. CAR, L-carnosine. (E-J) Relative mRNA expression levels of genes involved in stress-activated pathway and arginine metabolism in larvae. n = 5 biological replicates per group. mRNA expression levels were quantified by RT-qPCR and normalized to *arnt* 2. atf4a, activating transcription factor 4a; atf4b, activating transcription factor 4b; ass1, argininosuccinate synthase 1; asl, argininosuccinate lyase; arg1, arginase 1; odc1, ornithine decarboxylase 1; arnt2, aryl hydrocarbon receptor nuclear translocator 2. (K) Ornithine concentrations have a decreased tendency in DKO larvae compared to WT, GLO1KO and AKR1A1AKO groups. n = 4 biological replicates per group. (L) Spermidine concentrations were decreased in DKO larvae compared to WT, GLO1KO and AKR1A1AKO groups. n = 3 biological replicates per group. (M) Putrescine concentrations were decreased in DKO larvae compared to WT, GLO1KO and AKR1A1AKO groups. n = 3 biological replicates per group. The bars indicate mean ± SD values. Statistical analysis were performed by Student's t-test and one-way ANOVA. ns, not significant, *p < 0.05, **p < 0.01, ***p < 0.001, ****p < 0.0001.

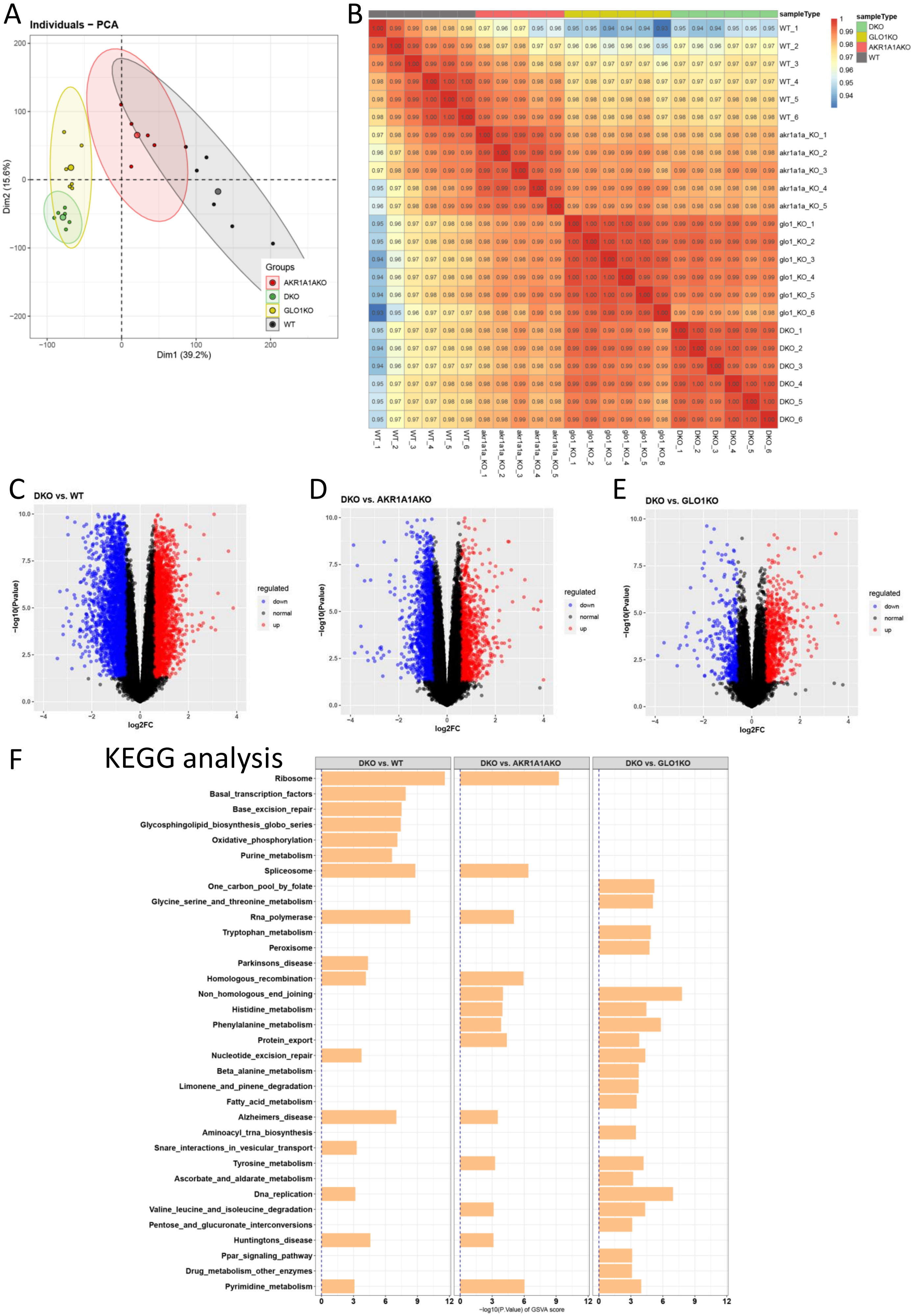
A
Individuals – PCA
Dim1 (39.2%)
Dim2 (15.6%)
Groups
AKR1A1AKO
DKO
GLO1KO
WT
B
sampleType
DKO
GLO1KO
AKR1A1AKO
WT
C
DKO vs. WT
D
DKO vs. AKR1A1AKO
E
DKO vs. GLO1KO
log2FC
−log10(Pvalue)
regulated
down
normal
up
F
KEGG analysis
DKO vs. WT
DKO vs. AKR1A1AKO
DKO vs. GLO1KO
Ribosome
Basal_transcription_factors
Base_excision_repair
Glycosphingolipid_biosynthesis_globo_series
Oxidative_phosphorylation
Purine_metabolism
Spliceosome
One_carbon_pool_by_folate
Glycine_serine_and_threonine_metabolism
Rna_polymerase
Tryptophan_metabolism
Peroxisome
Parkinsons_disease
Homologous_recombination
Non_homologous_end_joining
Histidine_metabolism
Phenylalanine_metabolism
Protein_export
Nucleotide_excision_repair
Beta_alanine_metabolism
Limonene_and_pinene_degradation
Fatty_acid_metabolism
Alzheimers_disease
Aminoacyl_trna_biosynthesis
Snare_interactions_in_vesicular_transport
Tyrosine_metabolism
Ascorbate_and_aldarate_metabolism
Dna_replication
Valine_leucine_and_isoleucine_degradation
Pentose_and_glucuronate_interconversions
Huntingtons_disease
Ppar_signaling_pathway
Drug_metabolism_other_enzymes
Pyrimidine_metabolism
−log10(P.Value) of GSVA score

**Suppl. Figure 5. RNA-seq analysis of GLO1/AKR1A1A DKO larvae.** (A) The PCA plot indicated distinct clustering, with WT (n = 6) in black, GLO1KO (n = 6) in yellow, AKR1A1AKO (n = 5) in red, and DKO larvae (n = 6) in green at 96hpf. GSVA, Gene Set Variation Analysis; hpf, hours of post fertilization; PCA, principal component analysis. (B) Correlation analysis revealed high consistency within each group and lower correlation across different groups. (C-E) DGE analysis was performed for pairwise comparisons (DKO vs WT, DKO vs AKR1A1AKO, DKO vs GLO1KO). DGE, differential gene expression. (F) GSVA of RNA-sequences showed the significantly upregulated pathways in pairwise comparisons (DKO vs WT, DKO vs AKR1A1AKO, DKO vs GLO1KO) at 96hpf. n = 5/6 clutches with 20 larvae in each group.

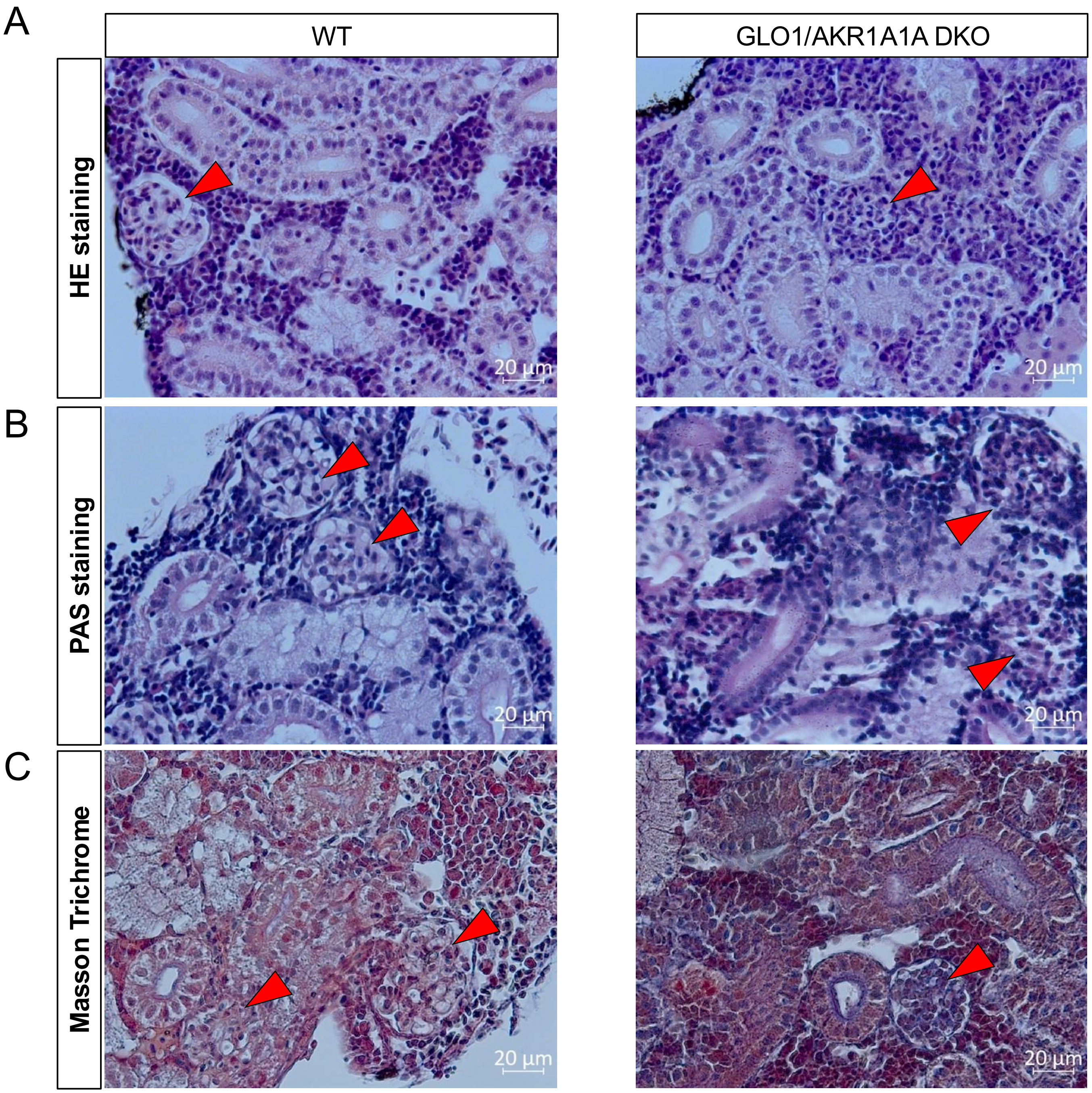


**Suppl. Figure 6. Altered glomeruli in adult DKO zebrafish kidneys.** (A) Representative HE-stained and (B) PAS-stained kidneys showed altered glomeruli with increased cell number and diffuse matrix expansion in GLO1/AKR1A1A DKO adult kidney compared to WT kidney. HE, hematoxylin and eosin; PAS, periodic acid-Schiff. (C) Representative Masson trichrome–stained images showed increased collagen deposition (blue) in the GLO1/AKR1A1A DKO adult kidney, compared to the wildtype kidney. Red arrows indicate glomeruli. n = 5 biological replicates per group. White scale bar = 20µm.

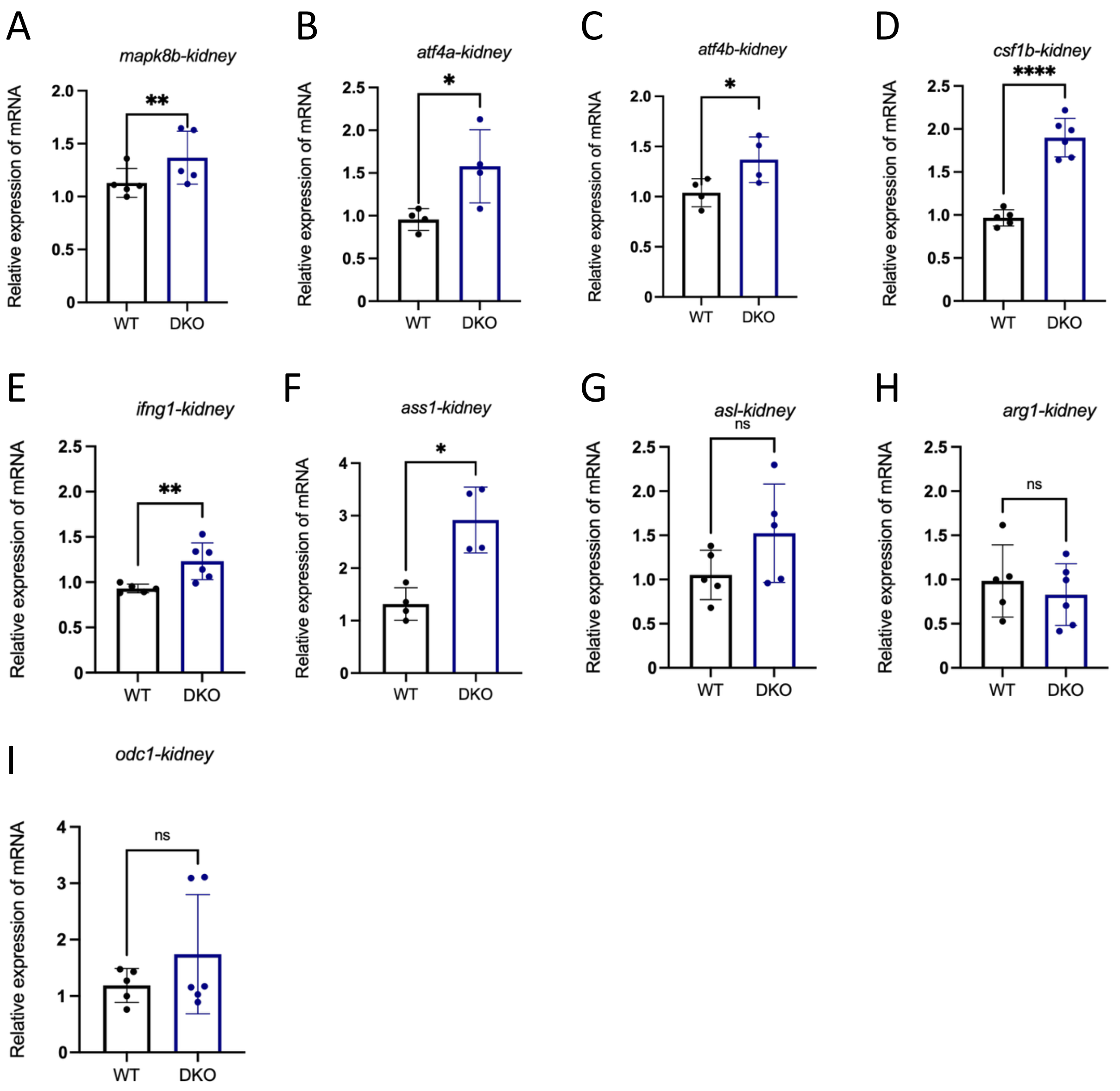


**Suppl. Figure 7. Transcript expression analysis of stress-responsive, inflammatory genes, and genes involved in arginine synthesis and catabolism in DKO zebrafish kidney.** (A-C) Relative mRNA expression levels of *mapk8b, atf4a* and *atf4b* were upregulated in DKO kidney compared to WT. n = 4/5 biological replicates per group. mapk8b, mitogen-activated protein kinase 8b; atf4a, activating transcription factor 4a; atf4b, activating transcription factor 4b. (D-E) Relative mRNA expression levels of *csf1b* and *ifng1* were upregulated in DKO kidney compared to WT. n = 5/6 biological replicates per group. csf1b, colony stimulating factor 1b; ifng1, interferon gamma 1. (F-I) Relative mRNA expression levels of *ass1* was upregulated in DKO kidney compared to WT, while *asl*, *arg1* and *odc1* were unchanged. n = 4/6 biological replicates per group. mRNA expression levels were quantified by RT-qPCR and normalized to *arnt 2*. ass1, argininosuccinate synthase 1; asl, argininosuccinate lyase; arg1, arginase 1; odc1, ornithine decarboxylase 1; arnt2, aryl hydrocarbon receptor nuclear translocator 2. The bars indicate mean±SD values. Statistical analysis was applied by Student's t-test. ns, not significant, *$p < 0.05$, **$p < 0.01$, ****$p < 0.0001$.

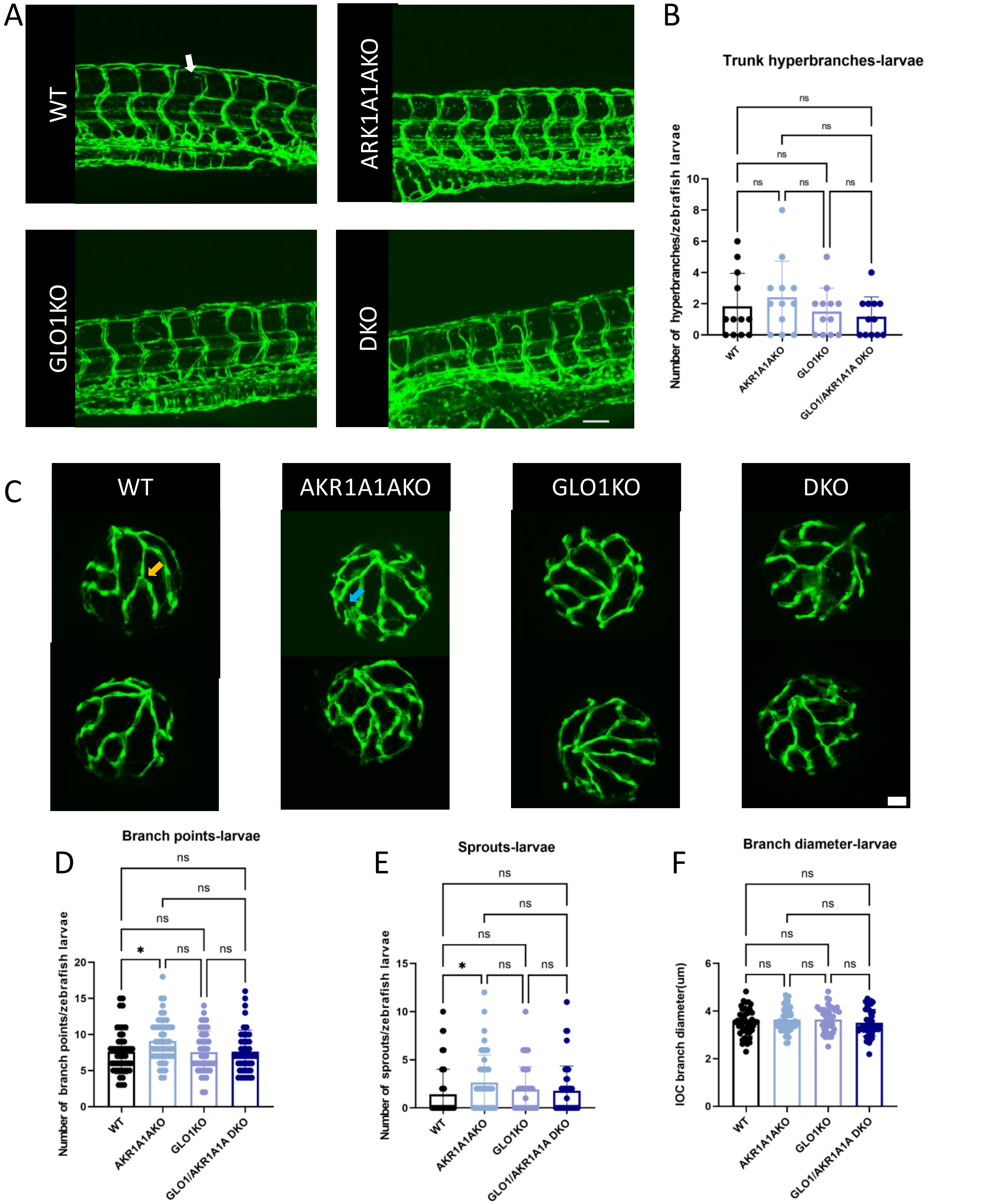


**Suppl. Figure 8. Unaltered trunk and hyaloid vasculature in GLO1/AKR1A1A DKO zebrafish larvae.** (A) Representative confocal images of trunk vasculature in larvae at 96hpf. White arrows indicated hyperbranches. White scale bar = 100µm. hpf, hours of post fertilization. (B) Quantification of hyperbranches showed unchanged in trunk vasculature among WT, GLO1KO, AKR1A1AKO and DKO larvae. n = 12 biological replicates per group. (C) Representative confocal images of the hyaloid vasculature in larvae at 120hpf. Yellow arrows indicated branch points, and blue arrows indicated sprouts. White scale bar = 20µm. (D-F) Quantification of branch points, sprout formation, and branch diameter length in the hyaloid vasculature showed no differences between DKO larvae and WT, GLO1KO and AKR1A1AKO larvae. n = 44-52 biological replicates per group. The bars indicate mean ± SD values. Statistical analysis was performed by one-way ANOVA. ns, not significant, *$p < 0.05$.

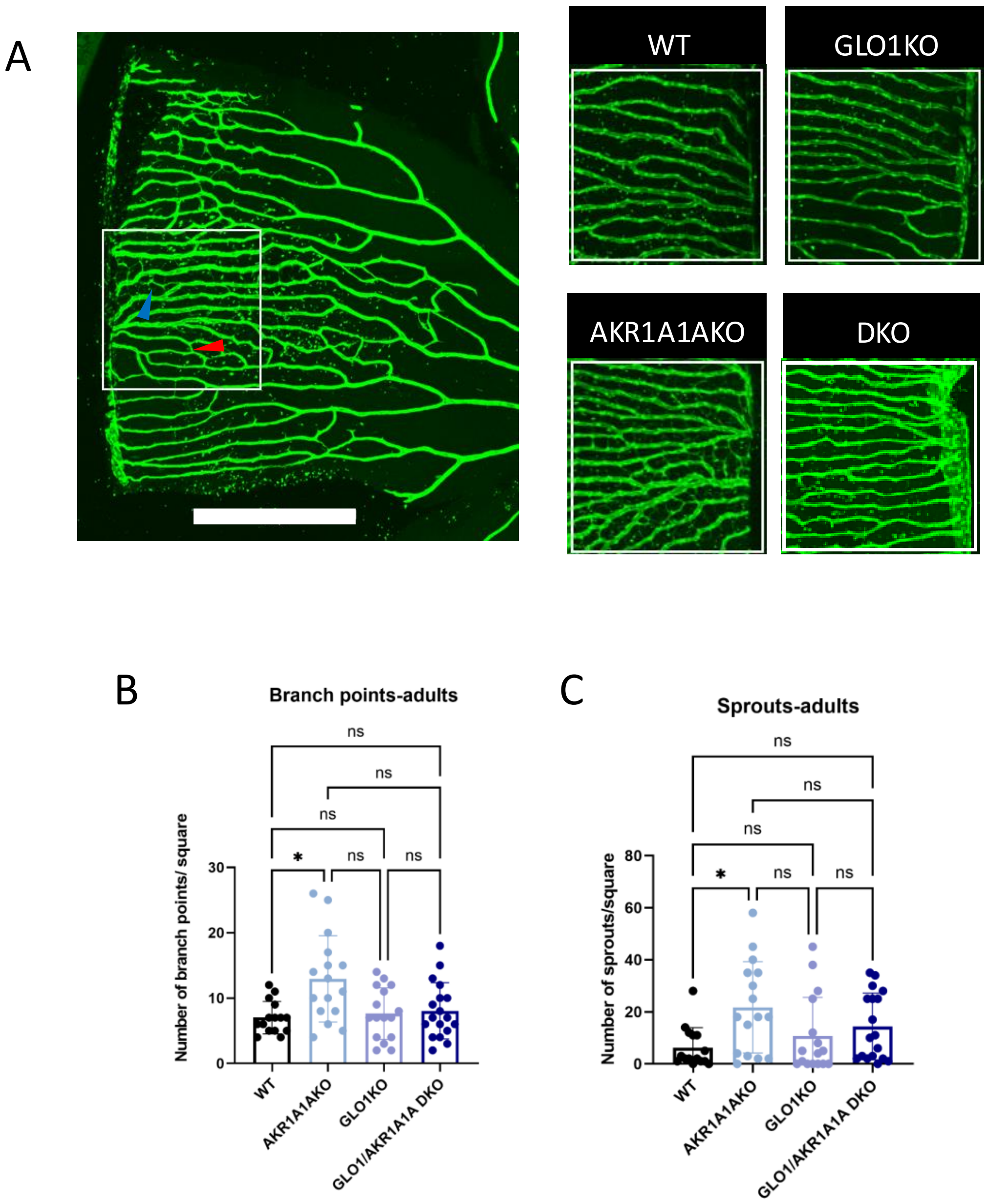


**Suppl. Figure 9. Unaltered retinal vasculature in GLO1/AKR1A1A DKO adult zebrafish.** (A) Representative confocal images of the retinal vasculature showed no alterations in DKO adult zebrafish at 12mpf compared to WT, GLO1KO and AKR1A1AKO adult zebrafish. Red delta indicated branch points, and blue delta indicated sprouts. White scale bar = 350µm. mpf, months of post fertilization. (B-C) Quantification of branch points and sprout formation in the retinal vasculature were unchanged in DKO adult zebrafish compared to WT, GLO1KO and AKR1A1AKO adult zebrafish. n = 15-18 biological replicates per group. The bars indicate mean ± SD values. Statistical analysis was performed by one-way ANOVA. ns, not significant, *$p < 0.05$.

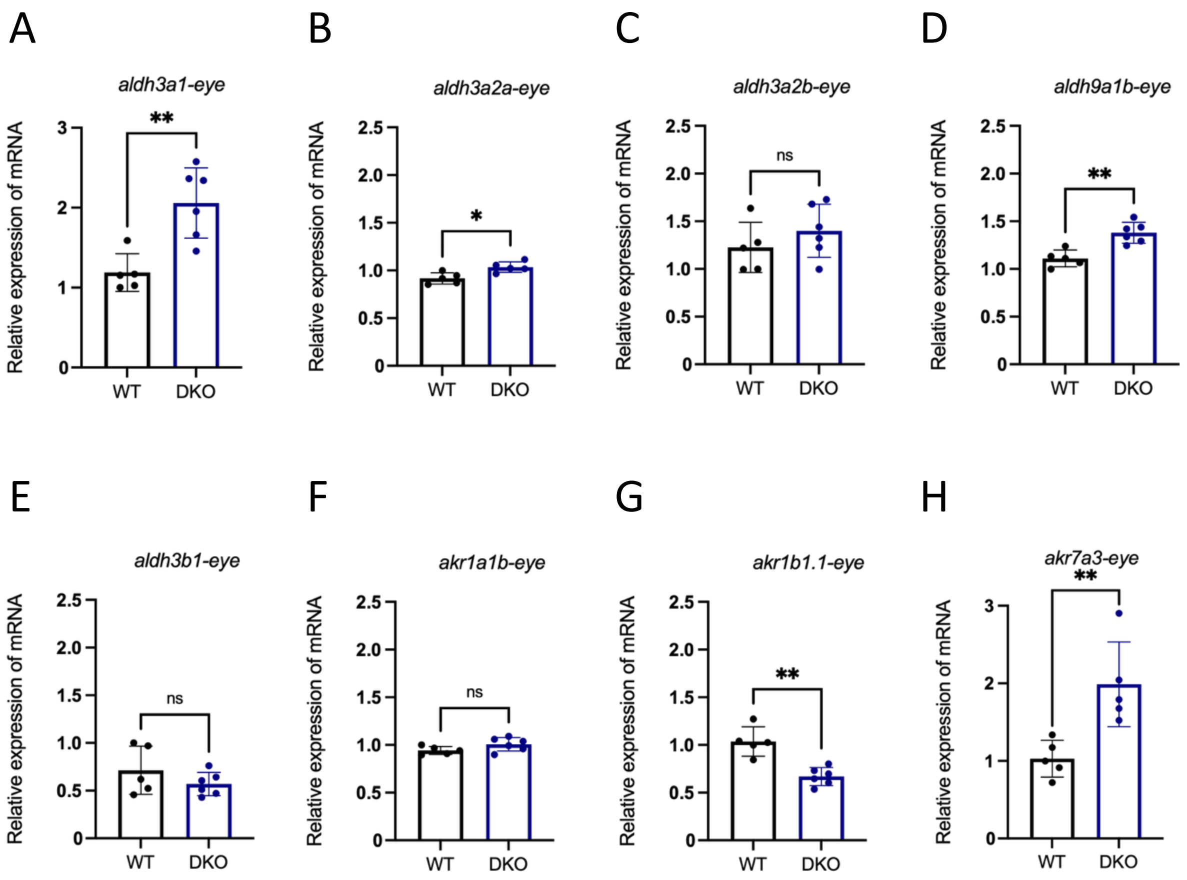


**Suppl. Figure 10. Transcript expression analysis of different *aldh* and *akr* genes in DKO zebrafish eyes.** (A-B, D, H) Relative mRNA expression levels of *aldh3a1*, *aldh3a2a*, *aldh9a1b* and *akr7a3* were upregulated, (C, E-F) *aldh3a2b*, *aldh3b1* and *akr1a1b* were unchanged, and (G) *akr1b1.1* was downregulated in DKO eyes compared to WT. n = 5/6 biological replicates per group. mRNA expression levels were quantified by RT-qPCR and normalized to *arnt 2*. aldh3a1, aldehyde dehydrogenase 3 family, member A1;aldh3a2a, aldehyde dehydrogenase 3 family, member A2a; aldh3a2b, aldehyde dehydrogenase 3 family, member A2b; aldh9a1b, aldehyde dehydrogenase 9 family, member A1b; aldh3b1, aldehyde dehydrogenase 3 family, member B1; akr1a1b, aldo-keto reductase family 1, member A1b; akr1b1.1, aldo-keto reductase family 1, member B1 (aldose reductase), tandem duplicate 1; akr7a3, aldo-keto reductase family 7, member A3; arnt2, aryl hydrocarbon receptor nuclear translocator 2. The bars indicate mean±SD values. Statistical analysis was applied by Student's t-test. ns, not significant, $*p < 0.05$, $**p < 0.01$.

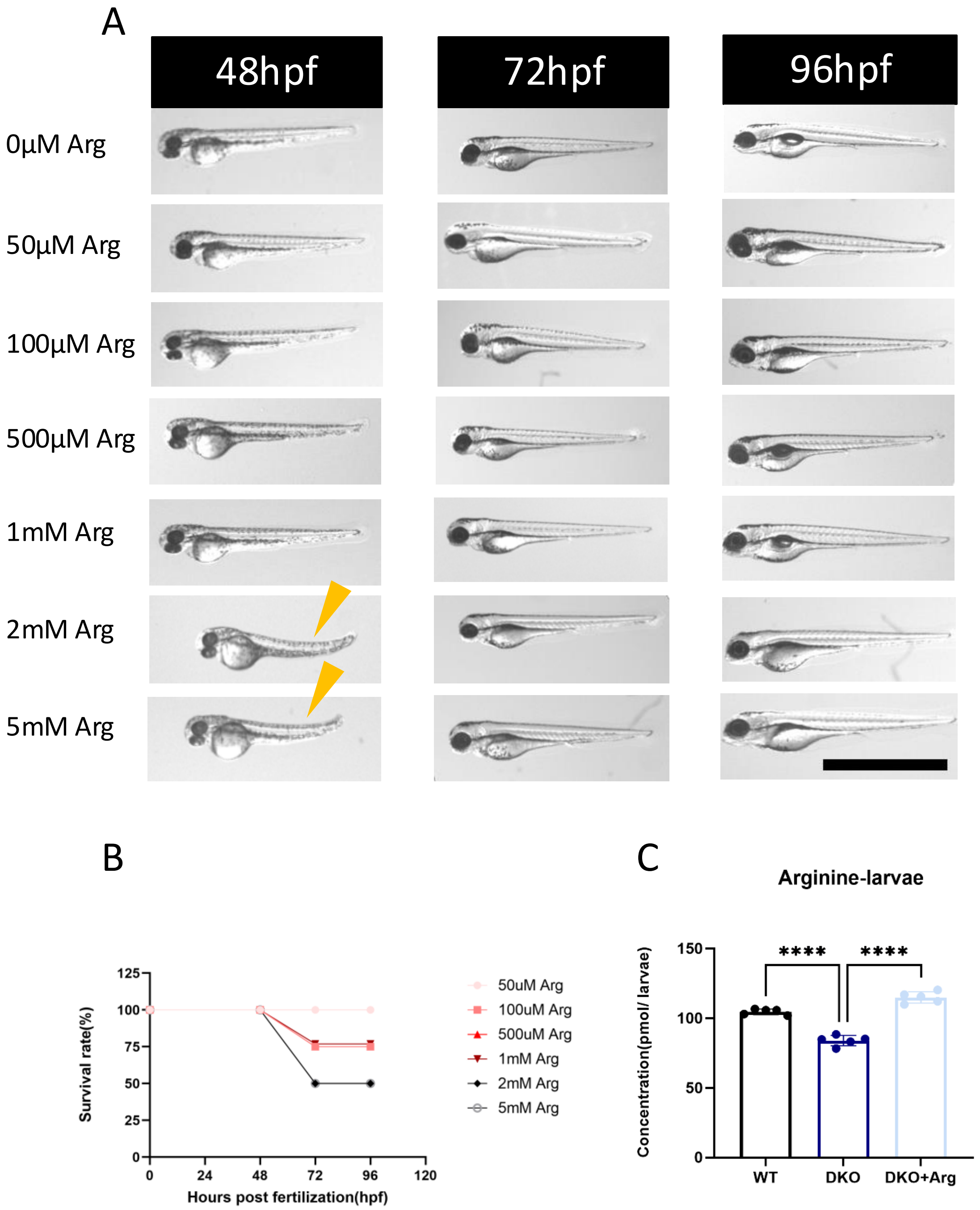


**Suppl. Figure 11. Morphological change and survival rate in larvae treated with L-Arginine.** (A) Representative microscopic images of zebrafish larvae treated with 0–5mM L-Arginine from 48hpf to 96hpf. Black scale bar = 2mm. Yellow delta indicated tail curvature. hpf, hours of post fertilization; Arg, L-Arginine. (B) Quantification of survival rates in larvae treated with 0–5mM L-Arginine during 48-96hpf. n = 13-16 biological replicates per group. (C) Arginine supplementation normalized the decreased arginine levels in DKO larvae to levels comparable to those in WT, n = 5 biological replicates per group. The bars indicate mean±SD values. Statistical analysis was applied by one-way ANOVA. **** $p < 0.0001$.

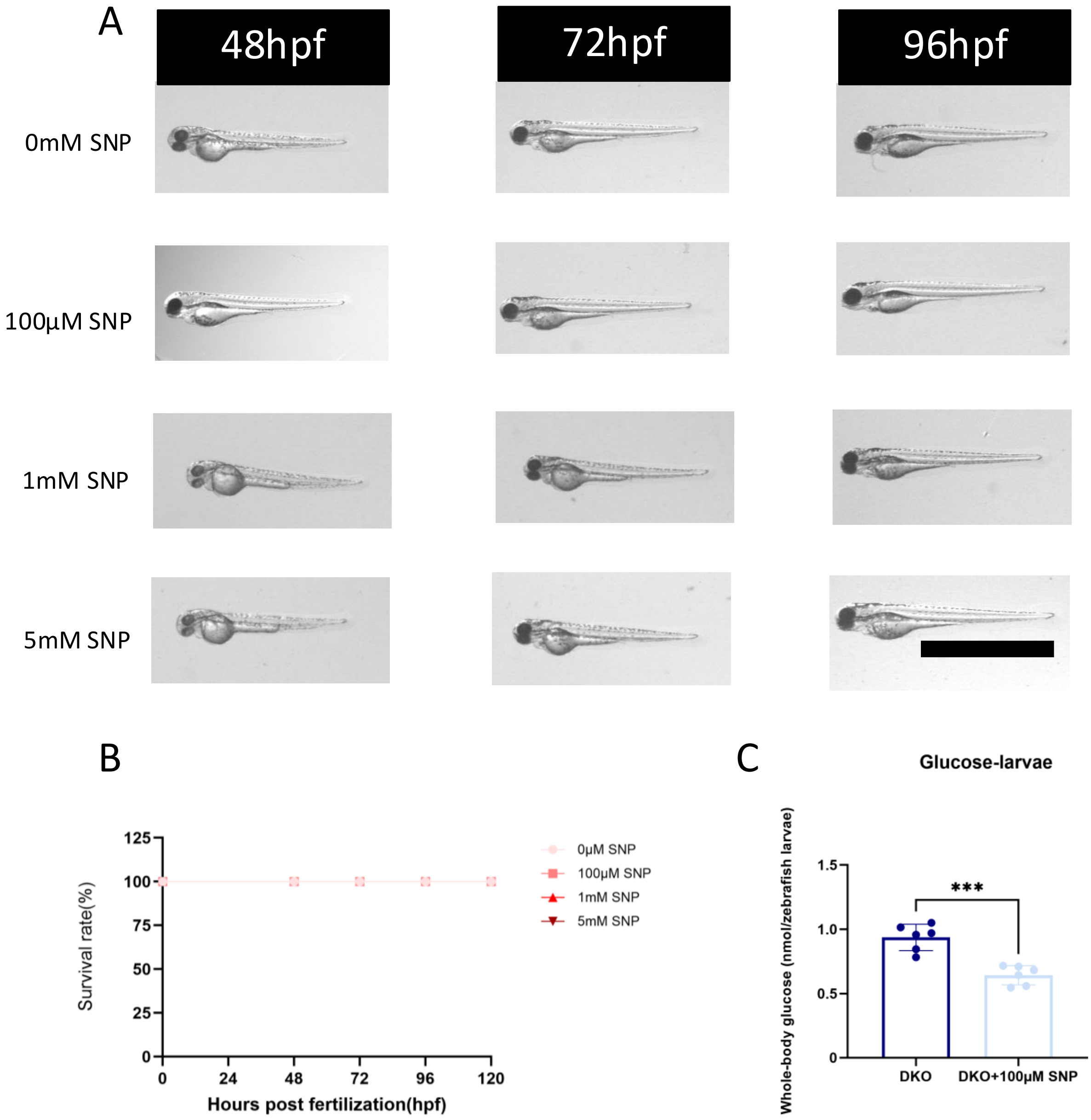


**Suppl. Figure 12. Morphological change and survival rate in larvae treated with SNP.** (A) Representative microscopic images of zebrafish larvae treated with 0–5mM SNP from 48hpf to 96hpf. Black scale bar = 2mm. hpf, hours of post fertilization; SNP, sodium nitroprusside. (B) Quantification of survival rates in larvae treated with 0–5mM SNP during 48-96hpf. n = 15 biological replicates per group. (C) Whole-body glucose levels in DKO larvae treated with 100µM SNP were reduced compared to DKO larvae, n = 6 biological replicates per group. Statistical analysis was applied by Student's t-test. ***$p < 0.001$.

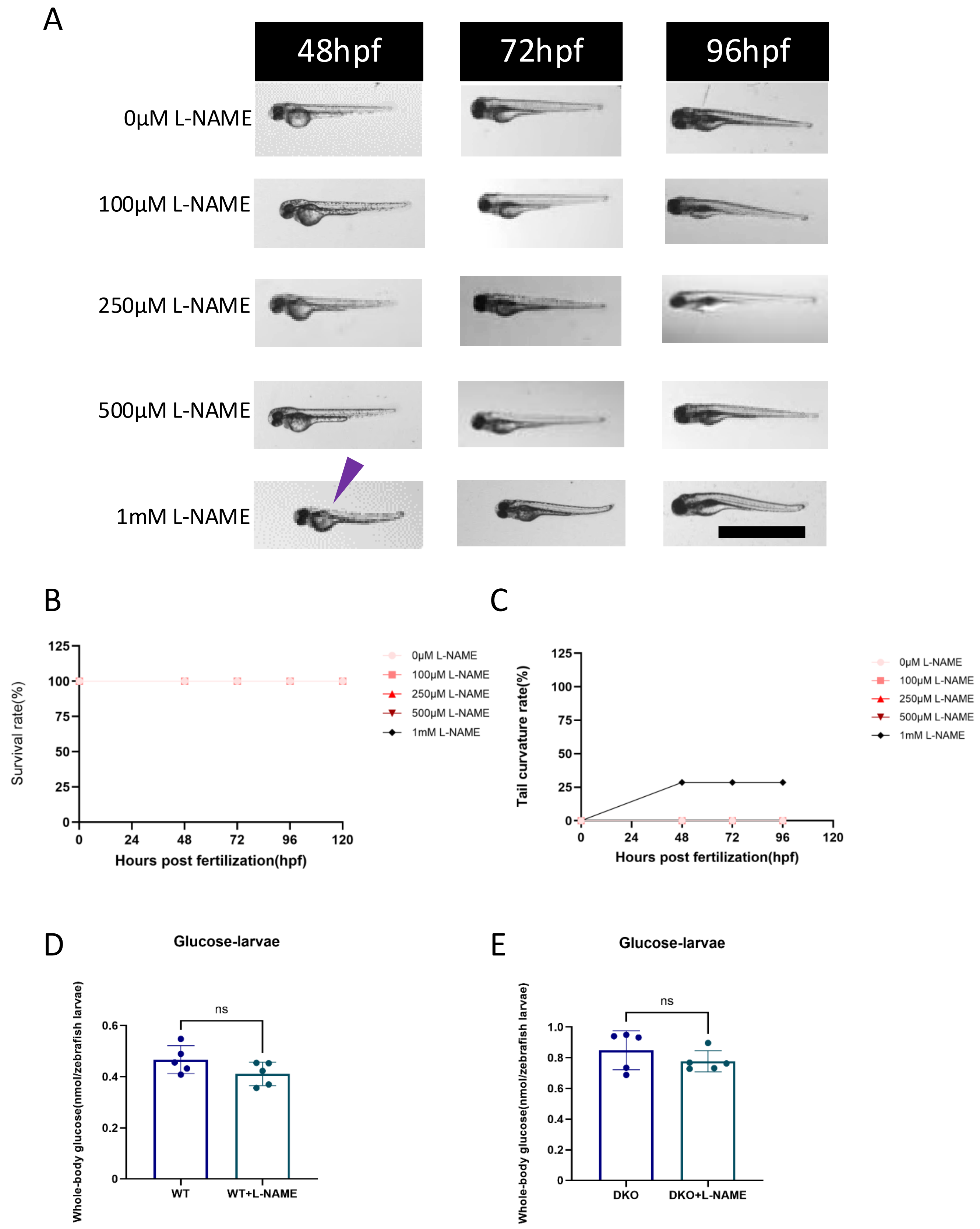


**Suppl. Figure 13. Morphological change and survival rate in larvae treated with L-NAME.** (A) Representative microscopic images of zebrafish larvae treated with 0–1mM L-NAME from 48hpf to 96hpf. Black scale bar = 2mm. Purple delta indicated tail curvature. hpf, hours of post fertilization; L-NAME, N(ω)-nitro-L-arginine methyl ester. (B) Quantification of survival rates in larvae treated with 0–1mM L-NAME during 48-96hpf. n = 14/15 biological replicates per group. (C) Quantification of tail curvature showed alterations in larvae treated with 1mM L-NAME at 48hpf. n = 14/15. (D-E) L-NAME treatment alone didn't alter the glucose levels in WT and DKO larvae, n = 5 biological replicates per group. Statistical analysis was applied by Student's t-test. ns, not significant.

# Supplementary Table 1: Primers list

| Primers Name | Primer sequence (5‘ to 3‘) |
|---|---|
| glo1-forward-genotyping | TAAACGTGCACCCTGAACTG |
| glo1-reverse-genotyping | CCCAACACTAGCACACACAC |
| akr1a1a-forward-genotyping | TCATTTGGGCAGGAAAACGT |
| akr1a1a-reverse-genotyping | GTAGCCACAGTCTAAAGCTGC |
| arnt2-forward-qPCR | AGCCAGACAGAGGTCTTCCA |
| arnt2-reverse-qPCR | CCGAGGTCAGCAAAGTCTTC |
| ins-forward-qPCR | GGTCGTGTCCAGTGTAAGCA |
| ins-reverse-qPCR | GGAAGGAAACCCAGAAGGGG |
| insra-forward-qPCR | AGAGGCCAGCGAGCTCTAC |
| insra-reverse-qPCR | CACTTGTGTGGGGGCTCT |
| insrb-forward-qPCR | GCCTCTGCGGATCACTACAT |
| insrb-reverse-qPCR | CTCCTGCGTGGTCTTGAAC |
| gck-forward-qPCR | AATCACCGCTGACCTGCTAT |
| gck-reverse-qPCR | GCCACTTCACATACGCAATG |
| pkmb-forward-qPCR | TGGGCTTATTAAGGGCAGTG |
| pkmb-reverse-qPCR | TGCACCACCTTTGTGATGTT |
| glut2-forward-qPCR | GCAGAAGAACCCTCACTC |
| glut2-reverse-qPCR | TCTCCGCCACAATAAACC |
| glut8-forward-qPCR | CATTTTGTCTGGTGTCGTCATGT |
| glut8-reverse-qPCR | CCTGCAATGAAAAAGCCCAT |
| gys1-forward-qPCR | TGTGACACCAAATGGCCTAA |
| gys1-reverse-qPCR | TGGATGCGTGCTTTACTCTG |
| ugp2a-forward-qPCR | CTGACGGGAGAGAATGAGGA |
| ugp2a-reverse-qPCR | GTCTTGGGGTTGACAATGAT |
| csf1b-forward-qPCR | CAACTAACCACCAGCACAGAG |
| csf1b-reverse-qPCR | GAGGCAGTAGGCAGTGAGAA |
| atf4a-forward-qPCR | CTCAAAGGCCGAGTGAAGAC |
| atf4a-reverse-qPCR | GCTGCGGTTTTATTCTGCTC |
| atf4b-forward-qPCR | TCTGAACCAGCCTCTCCAGT |
| atf4b-reverse-qPCR | GCAACTCTACAGGGGATGGA |
| infg1-forward-qPCR | AATGACAGCGTGGATGAAGC |
| infg1-reverse-qPCR | TGCCGTCTCTTGCGTTCT |
| mapk8b-forward-qPCR | CCGGCCTCGCTAGCACA |
| mapk8b-reverse-qPCR | ACCTCGGTGGACATGGACGA |
| arg1-forward-qPCR | CAAACAGGGCTTCTCTCTGC |
| arg1-reverse-qPCR | CAGATCCACAGCAGCGTTTA |
| odc1-forward-qPCR | GTTTGCAGGCTGAGTGTGAA |
| odc1-reverse-qPCR | ATGGCCTGGCTGTATGTTTC |
| ass1-forward-qPCR | ATGAGGACCGATACCTGCTG |
| ass1-reverse-qPCR | CAAAGCGAACCTGGTCATTT |

| Primers Name | Primer sequence (5‘ to 3‘) |
|---|---|
| asl-forward-qPCR | CCTTCAGACGGTCAGCTCTC |
| asl-reverse-qPCR | GAGCGGTGACGCTACTTTTC |
| slc7a1a-forward-qPCR | TTCGACTGCATCGCAACTAC |
| slc7a1a-reverse-qPCR | CAGATGAGGAGCGAGGAGAC |
| slc7a1b-forward-qPCR | ATGCTGAGTTTGGAGCTCGT |
| slc7a1b-reverse-qPCR | GCAACGCTGGATGTTCCTAT |
| col4a3-forward-qPCR | GGAAGTTGTTTGCCGATGTT |
| col4a3-reverse-qPCR | GTCATTTCGAGAGGCGTAGC |
| col4a4-forward-qPCR | TCTGGACCGGATTTTCATTC |
| col4a4-reverse-qPCR | GACCTTGGCACTCCACAAAT |
| col4a5-forward-qPCR | CAAAAGGTTTGGATGGTGCT |
| col4a5-reverse-qPCR | GACCTGCTGGTCCTGGAATA |
| lama1-forward-qPCR | GCTGGAGCTCATCAACAACA |
| lama1-reverse-qPCR | TGATTTTCTCCAGGGTTTCG |
| lamb1a-forward-qPCR | CAACCTGCTGGACTCACGTA |
| lamb1a-reverse-qPCR | CTCCACTGCCTCTCCAGTTC |
| aldh3a1-forward-qPCR | CACTGTTGATACTTTACCTTTTGGAG |
| aldh3a1-reverse-qPCR | CAAACGTGTGTTTCCCATGA |
| aldh3a2a-forward-qPCR | TGATGAATCTGAGTGTTACATTGC |
| aldh3a2a-reverse-qPCR | TGGCCCAAAGATCTCTTCC |
| aldh3a2b-forward-qPCR | CACTTCTCTGTCAGCTCTCTGC |
| aldh3a2b-reverse-qPCR | GATAGCGGCCCATACCACT |
| aldh3b1-forward-qPCR | CATGACTCTTCCTGGTTTACCC |
| aldh3b1-reverse-qPCR | TGATAGTTGCCCATCCCACT |
| aldh9a1b-forward-qPCR | GGAGCAAGCCAAGAACGA |
| aldh9a1b-reverse-qPCR | GGATCTGCAGGGCTGAAA |
| akr1a1b-forward-qPCR | CGTCTCTATTAAAAACTCTGAAAGACC |
| akr1a1b-reverse-qPCR | AAGGGGTATCGCCTCGTT |
| akr1b1.1-forward-qPCR | CAAACCTGCCAACAATCAGA |
| akr1b1.1-reverse-qPCR | CGTGACTGTTATGCCTTTGG |
| akr7a3-forward-qPCR | GTCACTCTGCTGGGCACCAT |
| akr7a3-reverse-qPCR | TTTGGTCGCGATTCGAACTGT |

# Supplementary Excel : Exact p values for Figure 4G-J

| Genes Name | Groups | P values |
|---|---|---|
| Figure 4G | | |
| Ins | GLO1/AKR1A1A DKO vs. WT | 0.0001 |
| insrb | GLO1/AKR1A1A DKO vs. WT | 0.0091 |
| | GLO1/AKR1A1A DKO vs. AKR1A1AKO | 0.0011 |
| | GLO1/AKR1A1A DKO vs. GLO1KO | 0.0028 |
| Figure 4H | | |
| gck | GLO1/AKR1A1A DKO vs. WT | 0.0033 |
| | GLO1/AKR1A1A DKO vs. AKR1A1AKO | 0.0022 |
| | GLO1/AKR1A1A DKO vs. GLO1KO | 0.0283 |
| pkmb | GLO1/AKR1A1A DKO vs. WT | 0.0075 |
| | GLO1/AKR1A1A DKO vs. AKR1A1AKO | 0.0040 |
| | GLO1/AKR1A1A DKO vs. GLO1KO | 0.0381 |
| glut2 | GLO1/AKR1A1A DKO vs. WT | 0.0379 |
| glut8 | GLO1/AKR1A1A DKO vs. WT | 0.0138 |
| | GLO1/AKR1A1A DKO vs. AKR1A1AKO | 0.0122 |
| | GLO1/AKR1A1A DKO vs. GLO1KO | 0.0153 |
| gys1 | GLO1/AKR1A1A DKO vs. WT | 0.0433 |
| ugp2a | GLO1/AKR1A1A DKO vs. WT | 0.0039 |
| | GLO1/AKR1A1A DKO vs. AKR1A1AKO | 0.0432 |
| | GLO1/AKR1A1A DKO vs. GLO1KO | 0.0140 |
| Figure 4I | | |
| ins | GLO1/AKR1A1A DKO vs. WT | 0.0042 |
| insra | GLO1/AKR1A1A DKO vs. WT | 0.0490 |
| insrb | GLO1/AKR1A1A DKO vs. WT | 0.0002 |
| | GLO1/AKR1A1A DKO vs. AKR1A1AKO | 0.0035 |
| | GLO1/AKR1A1A DKO vs. GLO1KO | 0.0464 |
| Figure 4J | | |
| gys1 | GLO1/AKR1A1A DKO vs. WT | 0.0498 |
