# Supplementary material for: The accumulation of methylglyoxal and acrolein impairs arginine homeostasis causing hyperglycemia and renal abnormalities in male zebrafish
Source: Nat Commun. 2026 Jul 28;17:7565. doi: 10.1038/s41467-026-76082-6 (PMC13416155; doi:10.1038/s41467-026-76082-6)

Fiugure 4K-L

2h postprandial liver1

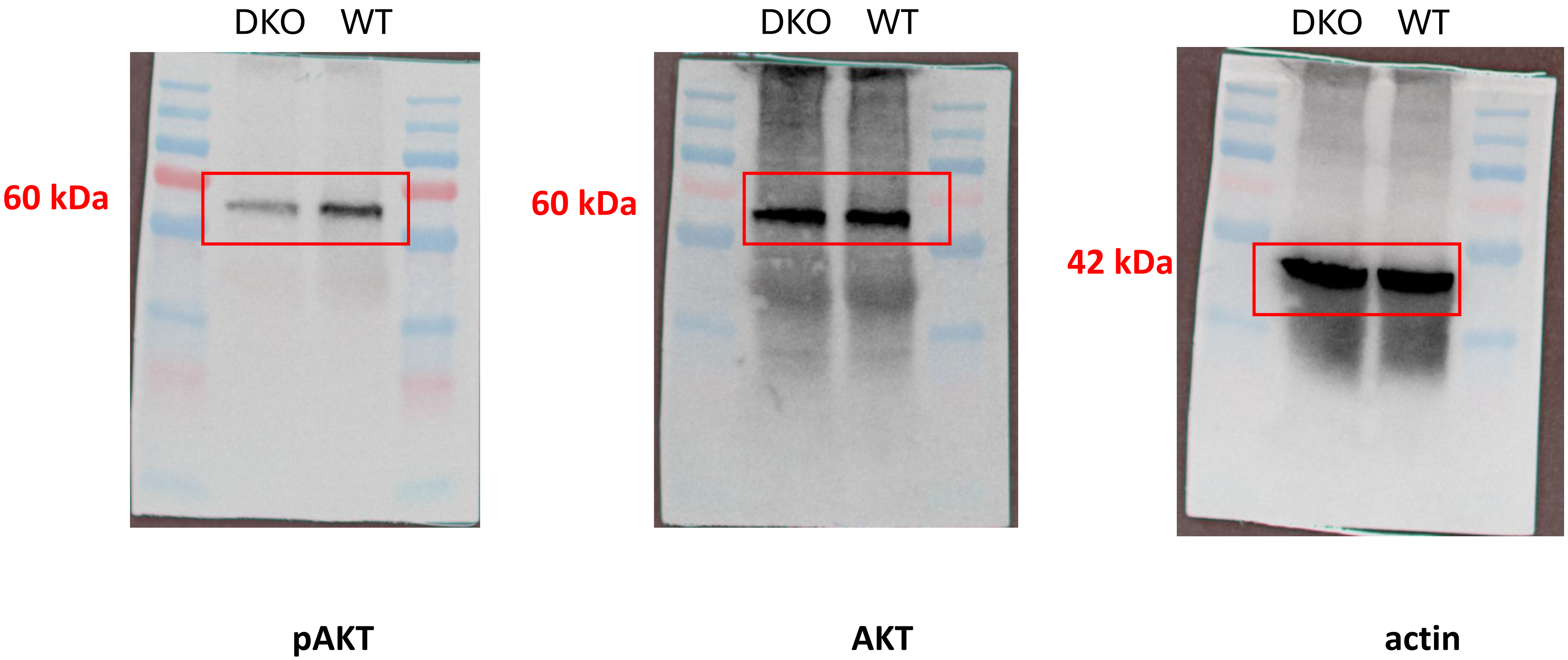

Fiugure 4K-L

2h postprandial liver2

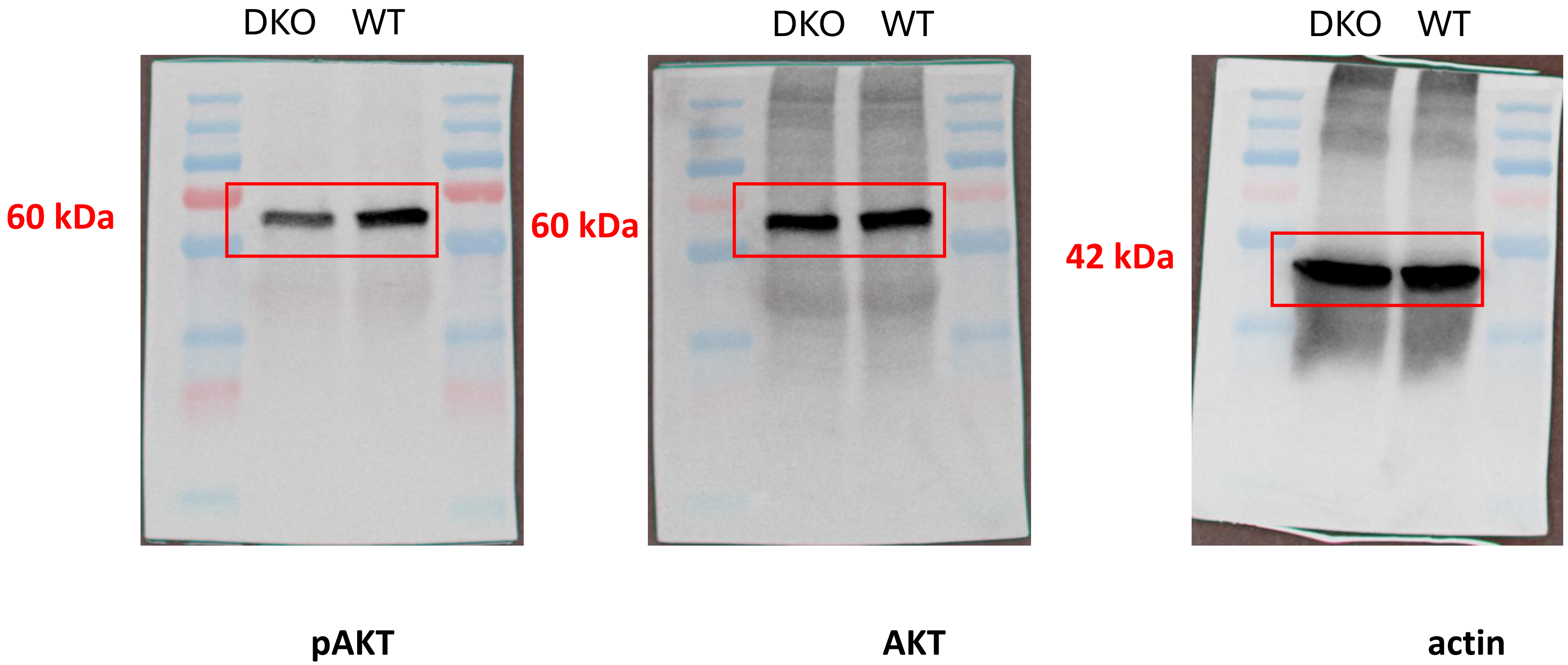

Fiugure 4K-L

2h postprandial liver3

DKO WT

60 kDa

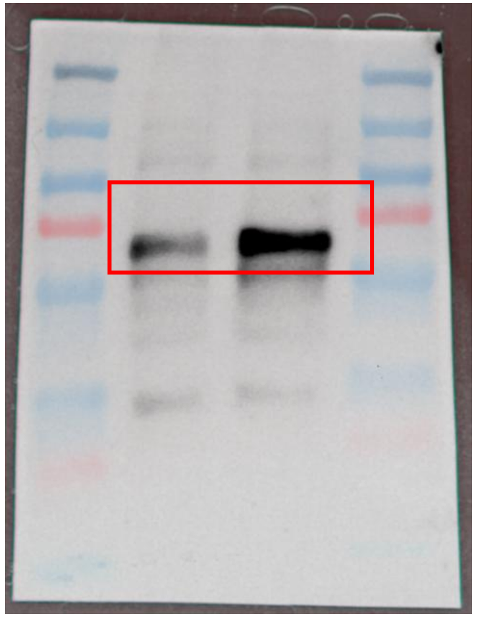

pAKT

DKO WT

60 kDa

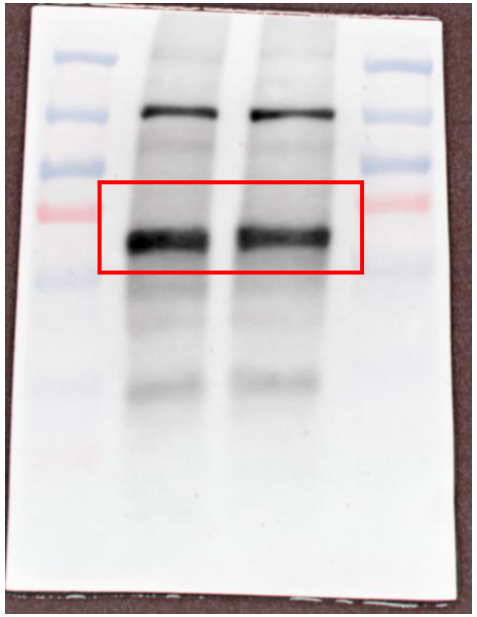

AKT

DKO WT

42 kDa

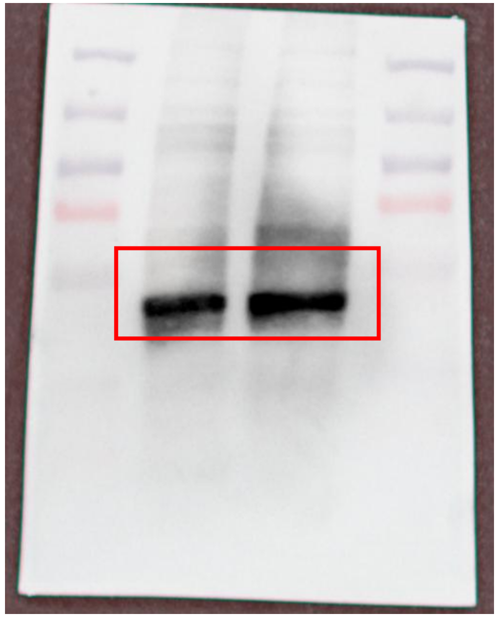

actin

Fiugure 4M-N

2h postprandial muscle1

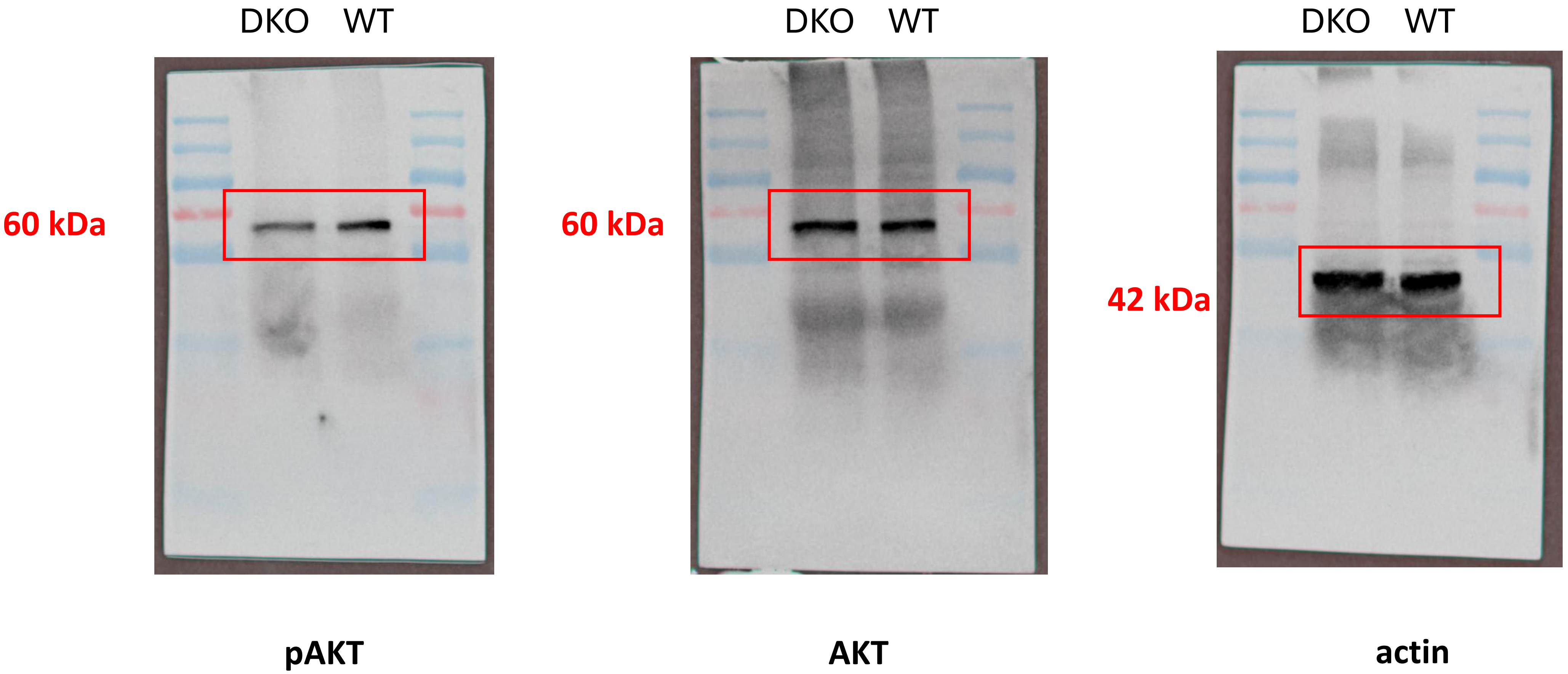

Fiugure 4M-N

2h postprandial muscle2

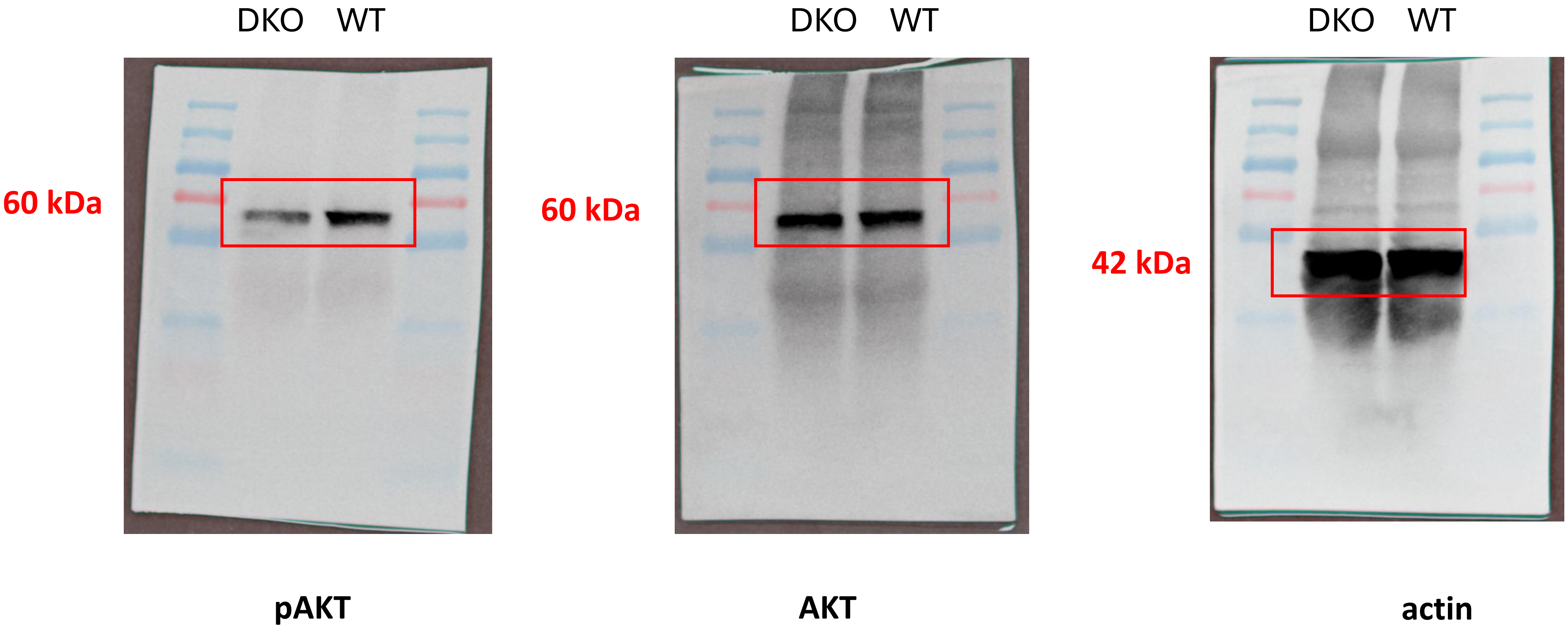

Fiugure 4M-N

2h postprandial muscle3

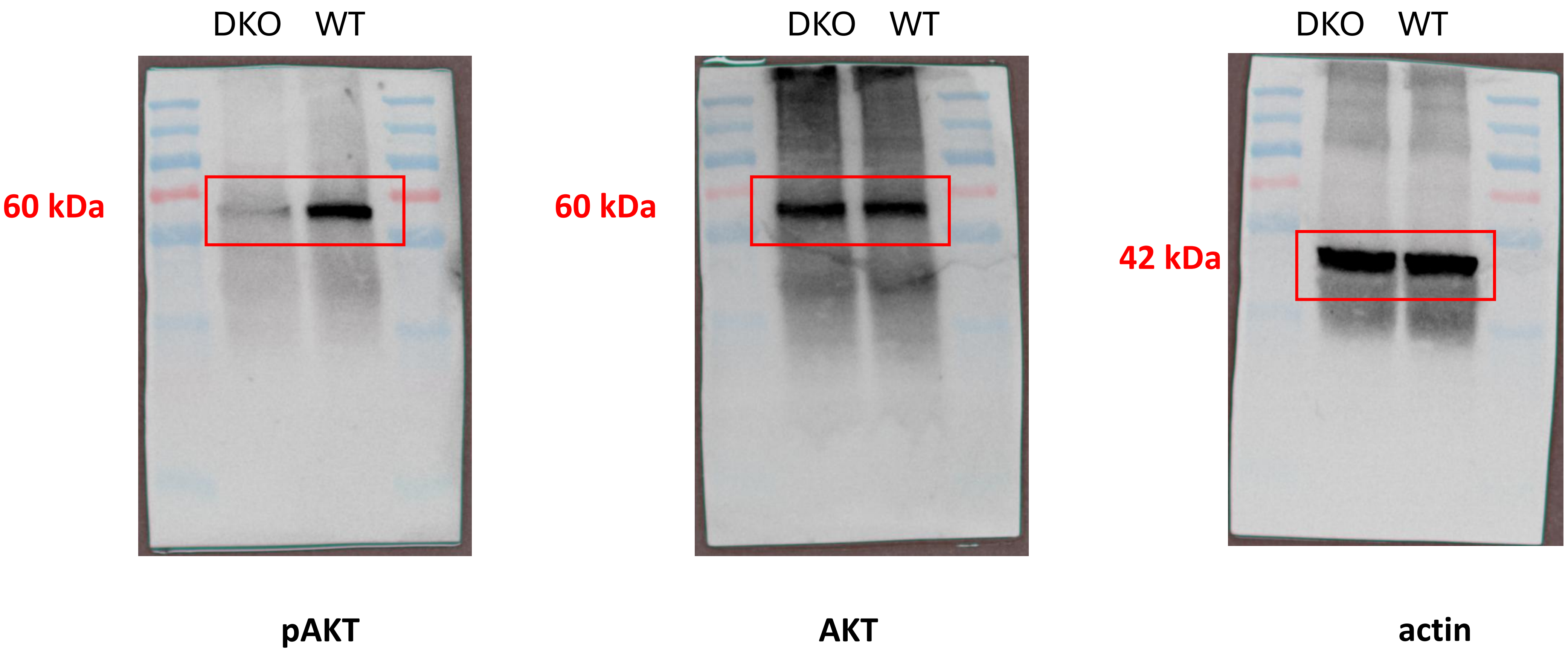

Fiugure 6C-D
larvae1

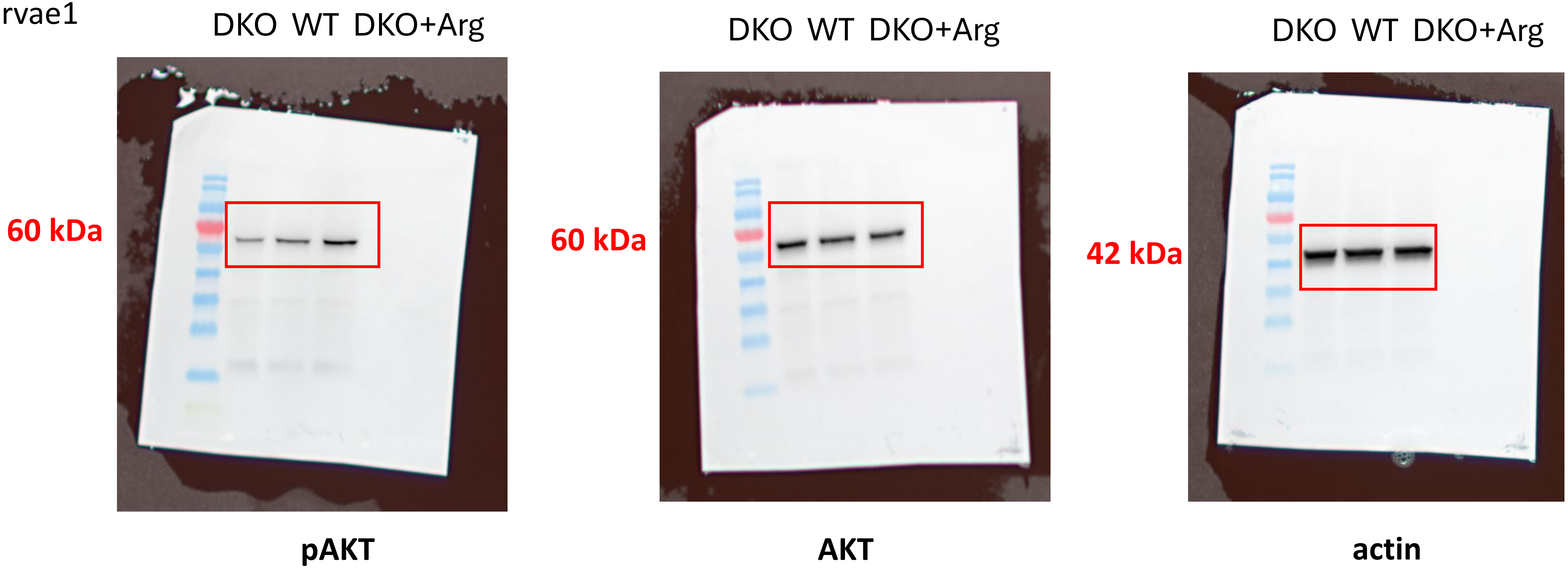

Fiugure 6C-D
larvae2

DKO WT DKO+Arg

60 kDa

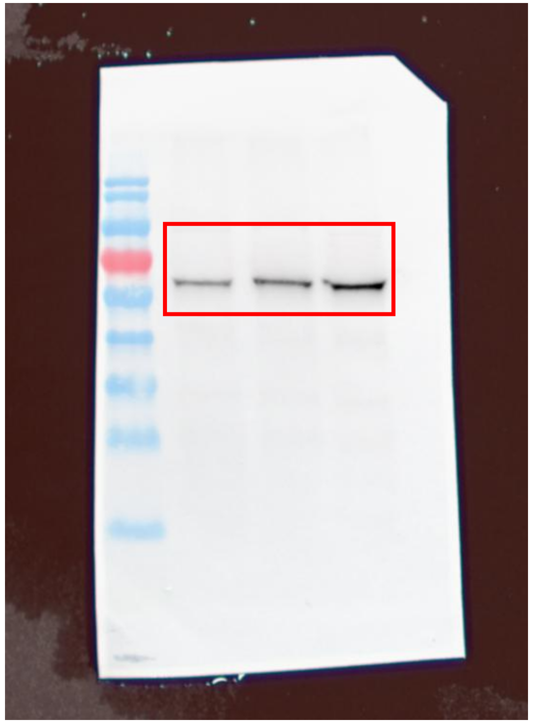

pAKT

DKO WT DKO+Arg

60 kDa

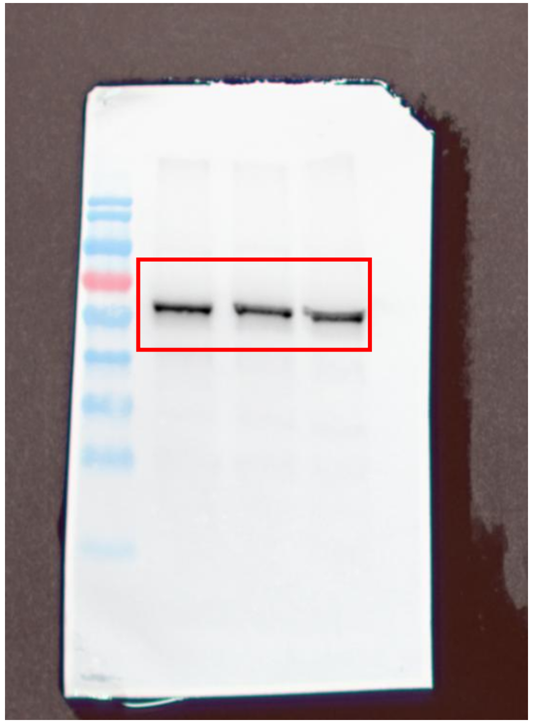

AKT

DKO WT DKO+Arg

42 kDa

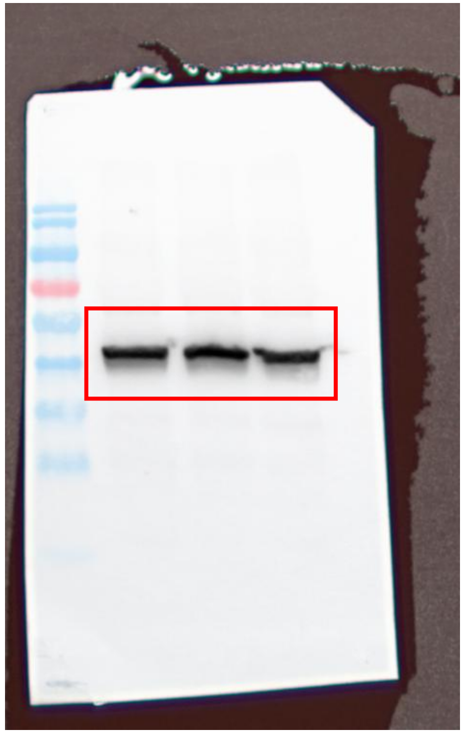

actin

Fiugure 6C-D
larvae3

DKO WT DKO+Arg

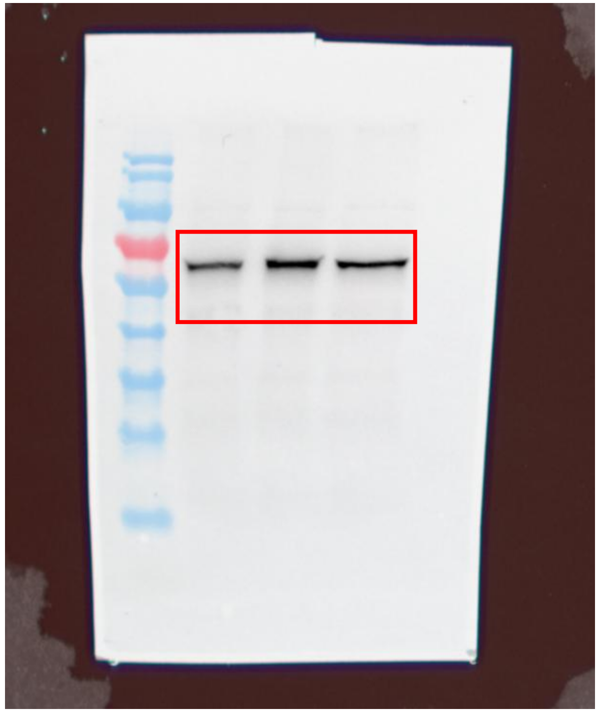

pAKT

DKO WT DKO+Arg

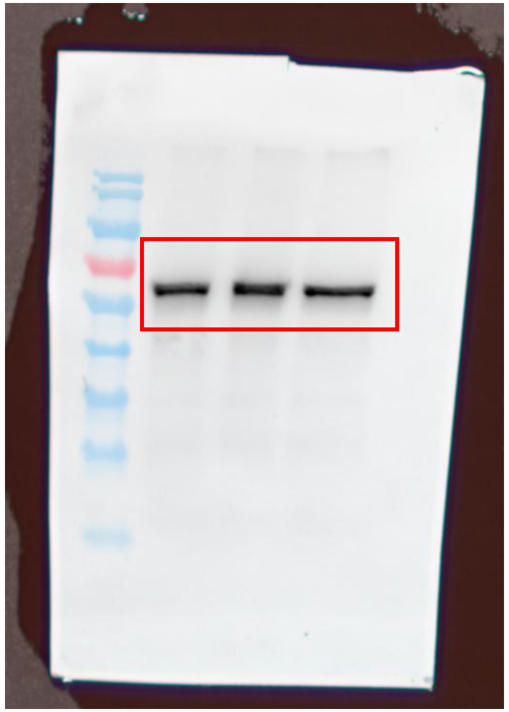

AKT

DKO WT DKO+Arg

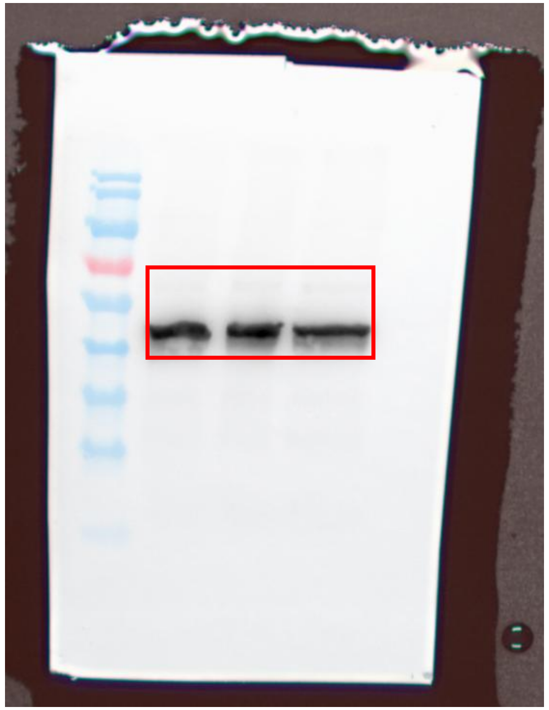

actin

Fiugure S4G-H

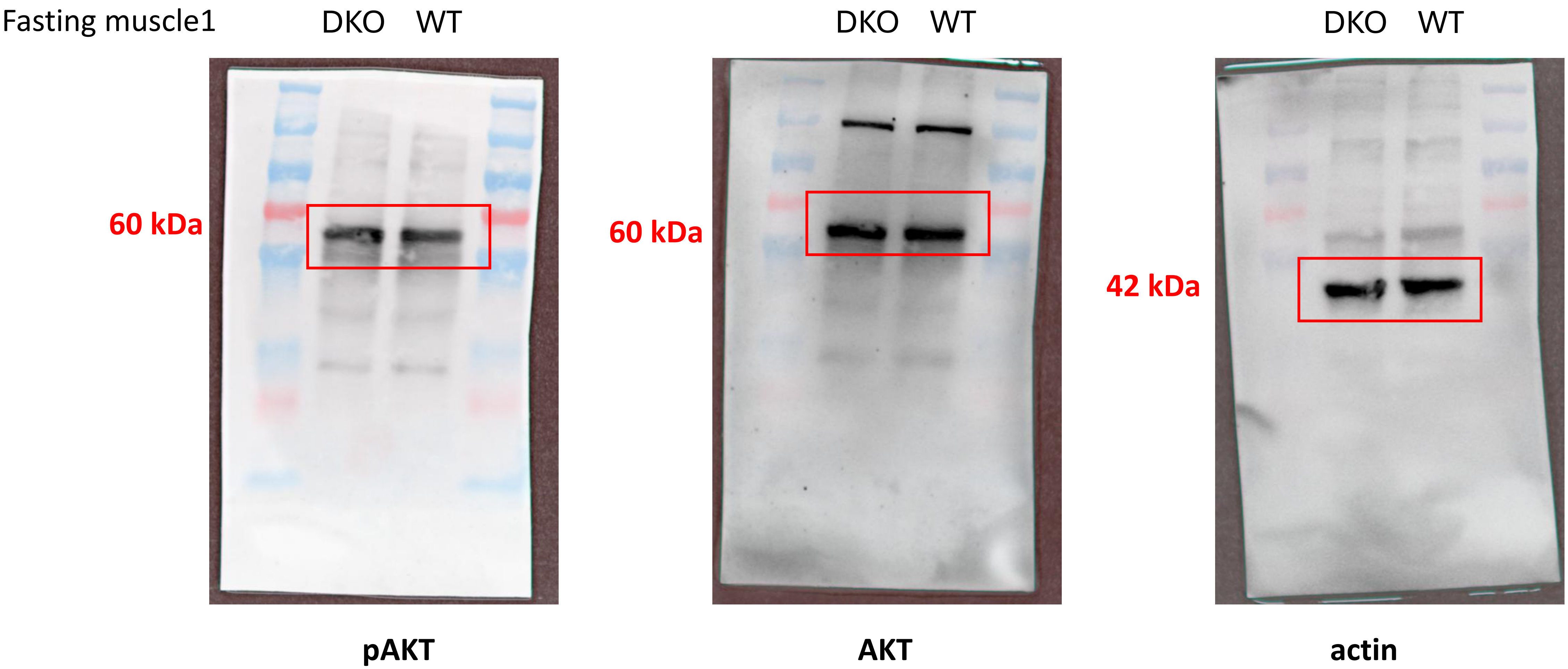

Fiugure S4G-H

Fasting muscle2

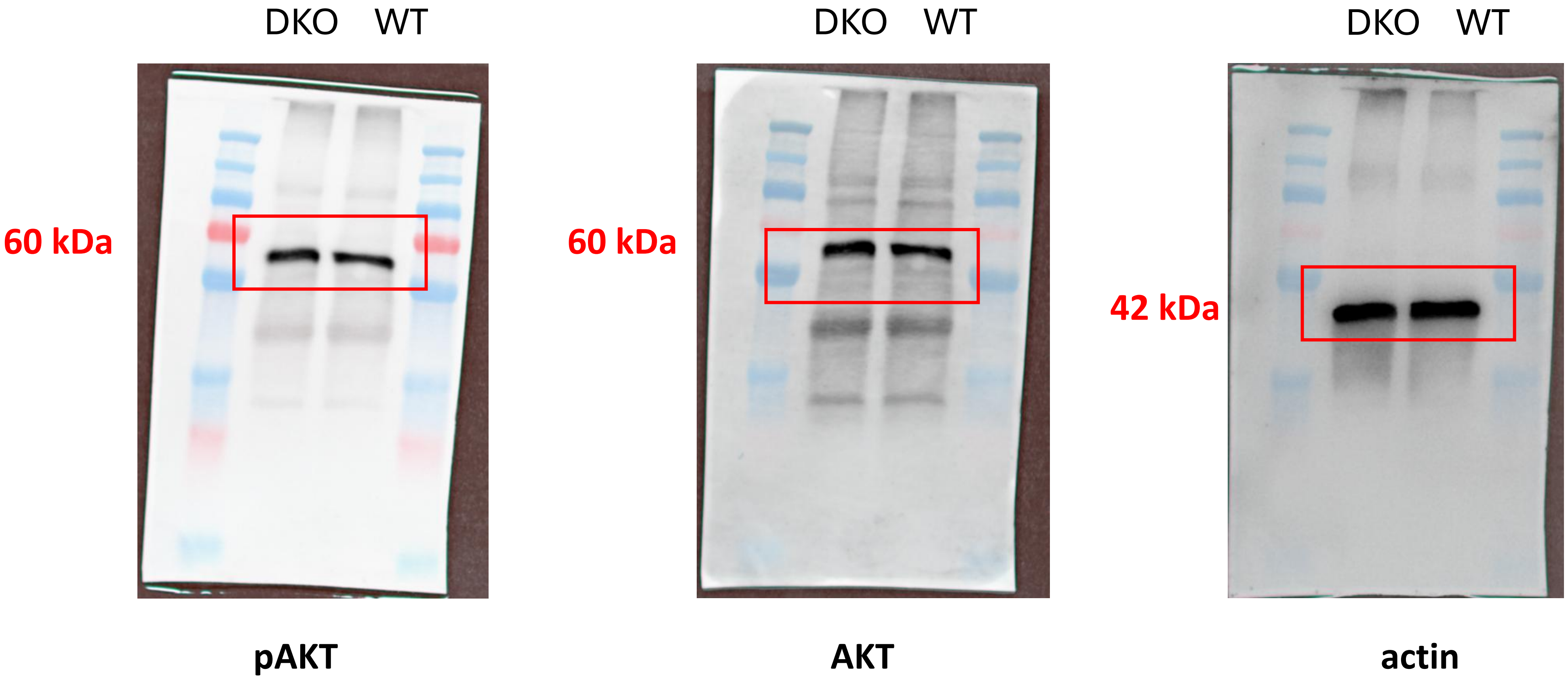

Fiugure S4G-H

Fasting muscle3

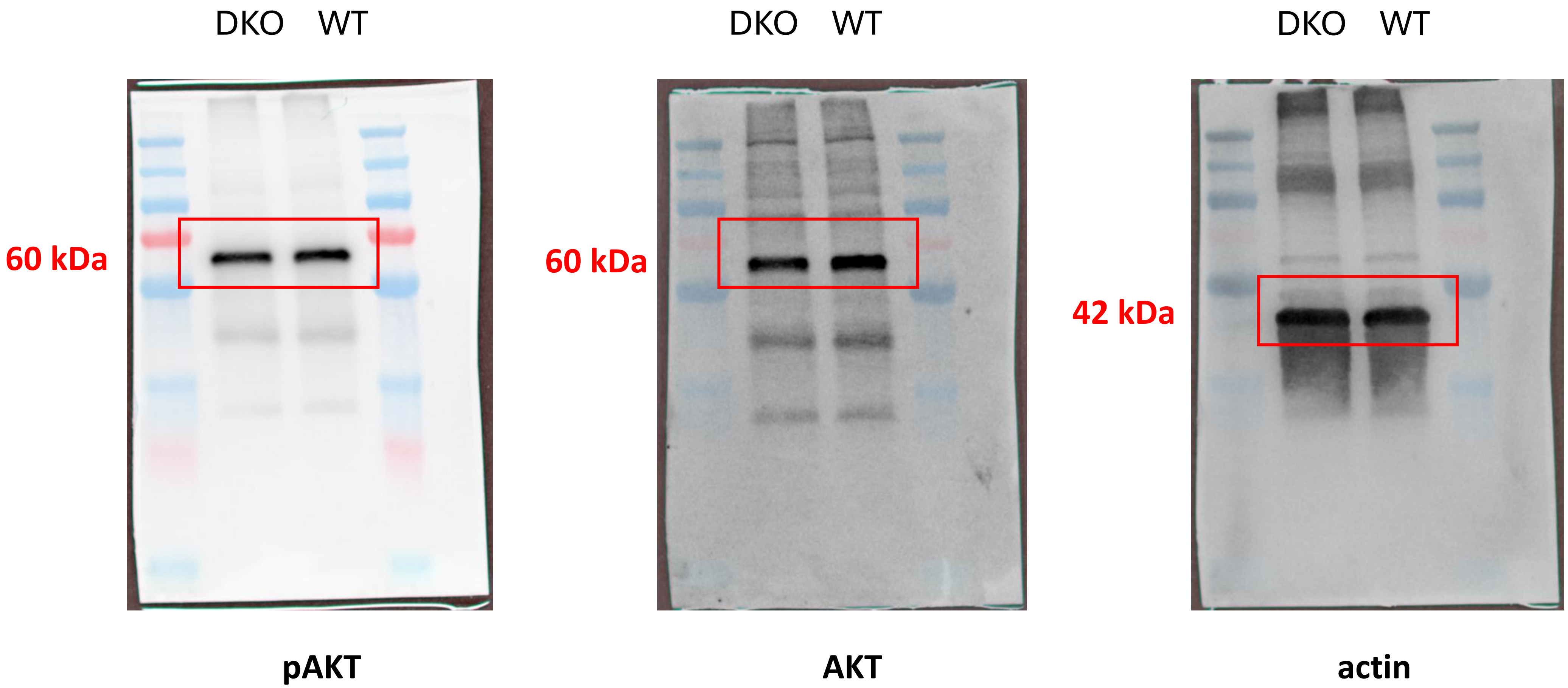

Fiugure S4I-J

Fasting liver1

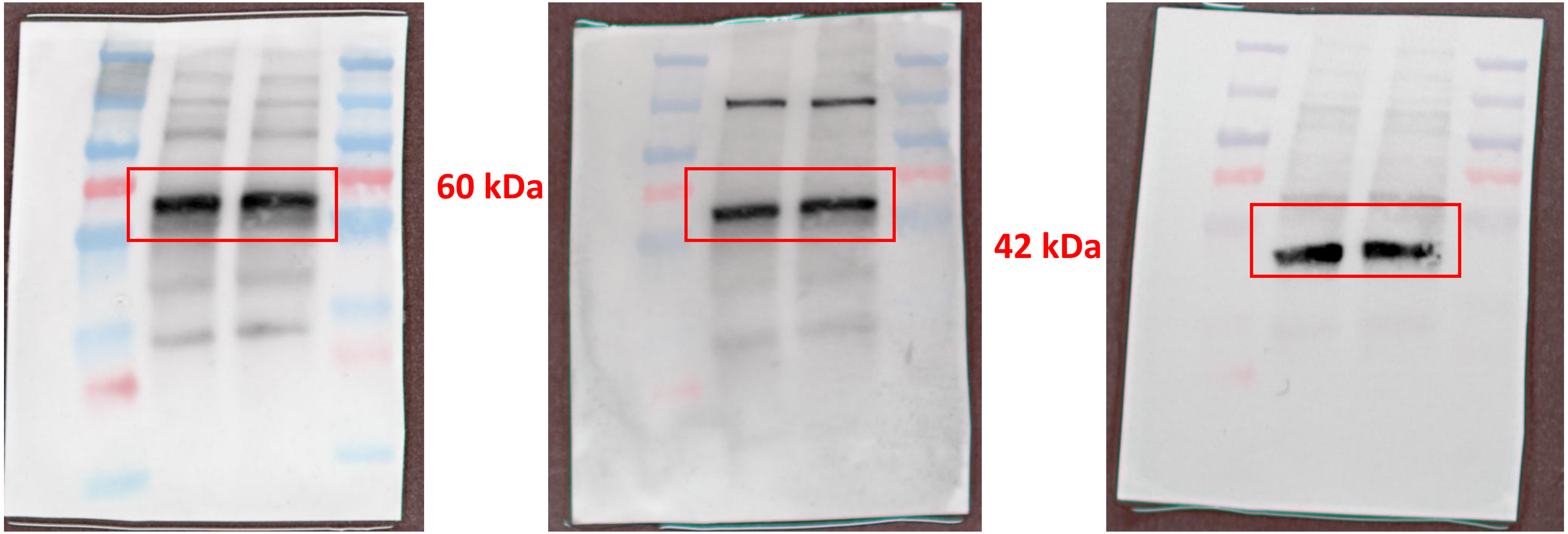

Fiugure S4I-J

Fasting liver2

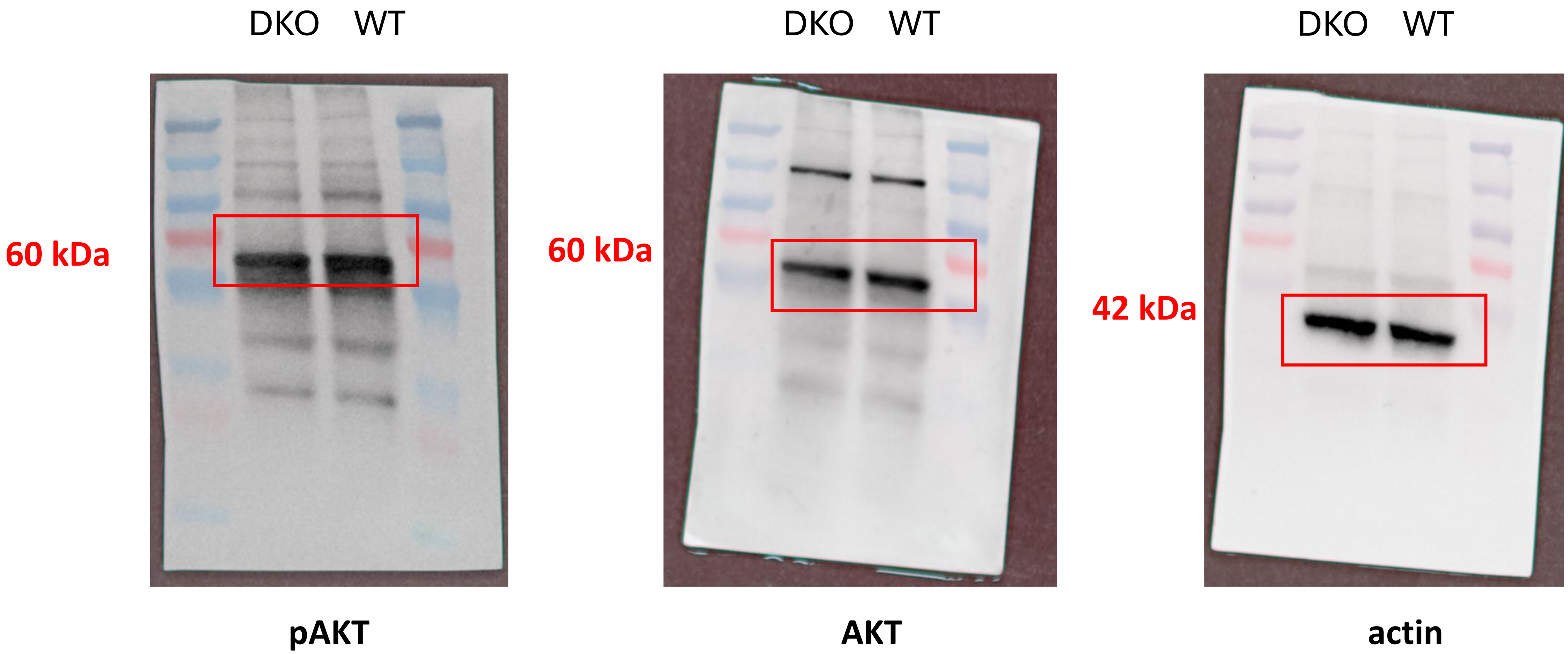

Fiugure S4I-J

Fasting liver3

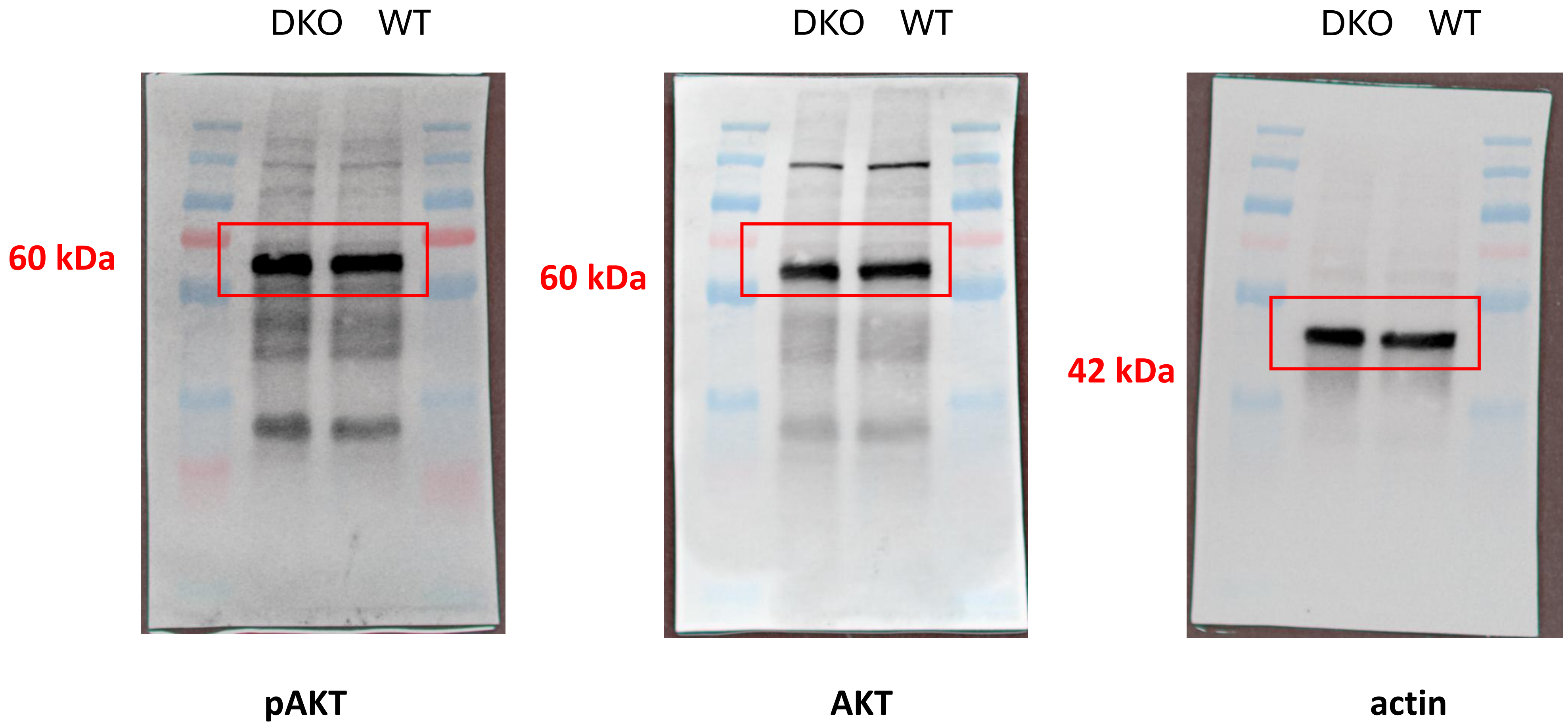

Supplement: Supplementary file 4 — Source Data [file 41467_2026_76082_MOESM4_ESM.zip › source data/western blot.pdf]
