# Supplementary material for: The accumulation of methylglyoxal and acrolein impairs arginine homeostasis causing hyperglycemia and renal abnormalities in male zebrafish
Source: Nat Commun. 2026 Jul 28;17:7565. doi: 10.1038/s41467-026-76082-6 (PMC13416155; doi:10.1038/s41467-026-76082-6)
Supplement: Supplementary file 3 — Transparent Peer Review file [file 41467_2026_76082_MOESM3_ESM.pdf]

nature portfolio

Peer Review File

The accumulation of methylglyoxal and acrolein impairs arginine homeostasis causing hyperglycemia and renal abnormalities in male zebrafish

Corresponding Author: Professor Jens Kroll

This file contains all reviewer reports in order by version, followed by all author rebuttals in order by version.

Version 0:

Reviewer comments:

Reviewer #1

(Remarks to the Author)
Review Comments
The study by Professor Kroll and collaborators is well executed, and the dataset is compelling. The authors investigate how arginine modifications caused by methylglyoxal (MGO) and acrolein affect glucose metabolism, insulin signaling, and organ pathology particularly in the kidney using glyoxalase 1 (glo1) and aldo-keto reductase 1a1a (akr1a1a) double-knockout (DKO) zebrafish. The data show that combined loss of these enzymes leads to accumulation of MGO and acrolein, suppression of arginine metabolism, downregulation of insulin signaling, elevated whole-body glucose, postprandial hyperglycemia, and glomerular basement membrane thickening. Arginine supplementation restores Akt phosphorylation, improves insulin sensitivity, and alleviates renal pathology. These findings identify Glo1 and Akr1a1a as cooperative regulators of carbonyl detoxification and metabolic homeostasis.
Several major issues require clarification or additional data:

1. Physiological relevance of the DKO model and carbonyl accumulation
The increases in MGO, MGO-derived AGEs, and acrolein in the DKO appear to be modest. This raises questions about how critical Glo1 and Akr1a1a truly are as a carbonyl-detoxifying system in vivo.
• How do the authors reconcile the relatively small changes in carbonyl stress markers with the substantial metabolic and signaling abnormalities observed?
• Do physiological or compensatory pathways buffer carbonyl accumulation in zebrafish, thereby limiting the magnitude of MGO/acrolein elevation?
Clarifying these points is essential for interpreting the broader relevance of the model.

2. Discrepancy between small increases in MGO/acrolein and large decreases in arginine
The reduction in arginine is striking compared with the mild elevation in carbonyls and carbonyl-derived AGEs. This discrepancy needs mechanistic clarification to understand causality.

• How do the authors explain this disproportionate drop in arginine?
• Could increased arginine consumption via stress-activated pathways, altered urea-cycle metabolism, or secondary effects on amino-acid transporters contribute more to the phenotype than carbonyl modifications themselves?

3. Tissue-specificity: pronounced arginine depletion in liver but not muscle
Arginine depletion is dramatic in the liver yet minimal/not in muscle. Given that approximately 80% of glucose disposal occurs in skeletal muscle:
• How do the authors interpret normal arginine levels in muscle in the context of insulin resistance and glucose intolerance?
• Would muscle RNA-seq and/or RT-qPCR of key insulin-signaling, glucose-transport, and amino-acid-metabolism genes help explain why muscle appears spared in terms of arginine depletion but not in terms of systemic glycemic control?
Such analyses are important for aligning tissue-level findings with whole-body metabolic phenotypes.

4. Potential involvement of the nitric oxide (NO) system
Given the profound reduction in arginine, a substrate for NO synthesis, there may be altered endothelial NO production and

vascular dysfunction.
• Can the authors measure vascular NO pathway components (NO levels, NOS activity/expression, nitrite/nitrate, endothelial function assays)?
• Could impaired NO-mediated vasodilation contribute to the metabolic and renal pathologies observed in the DKO?
5. Limited characterization of renal pathology
While glomerular basement membrane thickening is shown, renal pathology is not explored in depth.
• Additional histological, ultrastructural, and molecular analyses (e.g., podocyte markers, ECM remodeling, oxidative stress, or inflammatory pathways) would strengthen the link between carbonyl stress, arginine depletion, and kidney damage.

6. Interpretation of arginine supplementation effects
Although arginine is reduced in the DKO, the absolute concentration remains relatively high compared with the amounts typically needed for NO-dependent signaling. A deeper mechanistic interpretation of the rescue effect is important.
• How do the authors reconcile this with the strong phenotypic rescue by arginine supplementation?
• Do supplemented arginine levels fully normalize physiological concentrations in relevant tissues?

Overall
This work addresses an important gap in understanding carbonyl stress, arginine metabolism, and metabolic homeostasis. However, the issues above should be addressed to clarify mechanistic relationships, strengthen physiological relevance, and ensure that conclusions are well supported by tissue-specific data.

Reviewer #2

(Remarks to the Author)
This study explores the additive/synergistic roles of GLO1 and AKR1A1A in detoxifying reactive carbonyl species. There is generally not so much of a synergistic genetic interaction, but more additive effects of their two previously published mutants (reference 9 and 33) – which limits the novelty. However, to add novelty and strengthen the reliability of the conclusions, further elaboration and experimental verification are required regarding the specific mechanisms of arginine depletion, the nature of the insulin signaling impairment (secretion defect / reduced sensitivity), and the observed organ-specific phenotypes (kidney / retina).
Major Comments
Comment 1. The authors propose that the accumulation of MG and ACR leads to arginine depletion through the formation of adducts (such as MG-H1). While Figure 2 demonstrates a decrease in free arginine and an increase in MG-H1, this correlation is insufficient to prove that the depletion is solely due to scavenging or consumption by RCS. It is crucial to rule out impairments in arginine biosynthesis or transport. Although Figure 4 shows a downregulation of insulin-related genes, did the authors assess the expression levels of arginine biosynthetic enzymes (such as ass1, asl) or cationic amino acid transporters (such as slc7a family)?
Comment 2. The conclusion states that arginine supplementation improves insulin sensitivity. However, the data show a significant reduction in ins mRNA in DKO larvae, suggesting potential beta-cell dysfunction or reduced beta-cell mass, whereas reduced pAKT indicates impaired signaling. Does arginine function by promoting insulin secretion from beta-cells or by enhancing peripheral insulin sensitivity? Since the Western Blot data are derived from whole-fish lysates, distinguishing between these sources is difficult. The authors should clarify this distinction or discuss it in greater depth.
Comment 3. Figure 5 and Supplemental Figure 7 indicate that the DKO model exhibits distinct glomerular basement membrane thickening, yet the retinal vasculature remains unaffected. The authors speculate on the existence of retina-specific protective mechanisms, but empirical data are lacking. It is recommended to assess differences in MG and ACR levels between retinal and renal tissues in adult DKO zebrafish, or to compare the expression of antioxidant enzymes (such as ALDHs or other AKRs) via qPCR.
Comment 4. In Figure 6E, L-NAME is used to demonstrate that the effect of arginine is dependent on the NO pathway. However, the experiment lacks a critical control: does treatment with L-NAME alone (without arginine supplementation) induce hyperglycemia in the WT or DKO groups? If L-NAME exerts a hyperglycemic effect independent of the arginine rescue pathway, the specificity of the conclusion is compromised. It is necessary to provide data for WT + L-NAME or to discuss the basal metabolic effects of L-NAME.
Comment 5. Although arginine supplementation restored pAKT levels and lowered glucose, its impact on in vivo MG-H1 levels remains unclear. Does arginine mechanistically reduce protein modification by competitively scavenging RCS, or does it merely serve as a substrate to promote NO synthesis regardless of RCS levels? It is recommended to measure MG-H1 levels following arginine supplementation to clarify this mechanism.

Minor comments:
• There is a need for a scheme of the metabolite system for glo1 and ark1a1a with their subtract and how they interact
• In fig3-A the authors compare DKO with other and conclude that insulin signaling pathway in GLO1/AKR1A1A DKO is downregulated in comparison to WT, but it appears like it is already the case for single KO versus DKO. Similar point for fig4, most gene expression looks down in single KO as well? is it significant?
• In the abstract authors use full length enzyme name and abbreviated one at random and without defined the abbreviation in the abstract
• typo for ark1a1a in line 92 (introduction)
• In line 215 they use an undefined abbreviation (full name come later in the text)
• The authors could explain the reason they chose to use specific transgenic lines, such as Tg(fli1:EGFP)
• In fig6-C the molecular marker size should be indicated instead of the approximative size of the band of interest
• The color of the bar graph should be change. The yellow bars are barely visible with white background.

• It would be beneficial to expand the discussion on why Glo1 single-knockout zebrafish exhibit only moderate MG elevation without severe hyperglycemia, in contrast to mammalian models where Glo1 knockout is often lethal or phenotypically severe. This comparison is important for understanding the differences of the zebrafish model.

Reviewer #3

(Remarks to the Author)
The manuscript by Li et al shows that increased MG and ACR in glo1/akr1a1a double knockout (DKO) zebrafish leads to hyperglycemia and diabetic nephropathy but not retinopathy. Transcriptomic analysis revealed impaired insulin and MAPK signaling and impaired arginine metabolism in the DKO fish. The authors demonstrate that decreased free arginine mediates the glucose dysregulation, as arginine supplementation corrects the molecular changes and hyperglycemia. They further identify NO synthesis as a mediator of the arginine effects. The manuscript is clearly written. The data are of good quality and support the conclusions. Nevertheless, several issues are identified.
Major issue
1. The authors only used a non-selective NOS inhibitor. Since selective NOS inhibitors are available, it may be a good idea to use them to identify the NOS isoform(s) responsible for the arginine effect.
2. It is unclear how increased MG and ACR induce transcriptomic changes in genes related to arginine biosynthesis and metabolism. Is it possible to mine the RNA-seq data to identify candidate transcription factors that mediate the changes?
3. Instead of using the pdx1 mutant fish the lab reported previously, the authors chose pdx1 morphants from a single morpholino antisense oligo for the arginine supplementation studies. Given the non-specific activity of morpholino oligos, it is recommended to use multiple oligos targeting the same gene and validate disruption of translation or splicing. Disregard this comment if the specificity and activity of the morpholino oligo have been validated previously.

Minor issue:
It looks like the glut4 (line 205) should be glut2.

Version 1:

Reviewer comments:

Reviewer #1

(Remarks to the Author)
Although the authors did address all the points of concern, several point of concern remains.

While the study is technically well executed, the observed differences are very small and their biological relevance remains questionable since carbonyl stress and detoxification involve a large network of enzymes. In this study, the authors selected two enzymes and investigated them in combination. Although these enzymes are likely relevant, they are certainly not the only ones involved. Multiple enzymatic systems interact in the detoxification of reactive carbonyl species, and this complexity is only superficially addressed. Indeed, the authors themselves acknowledge this interaction in their response to my Question 1.

Several questions raised in the first review round are not addressed directly but rather indirectly through gene expression data. For example, the authors refer to transcriptional changes when discussing the modest increases in MGO and acrolein levels or alterations in NO concentrations. Such indirect evidence is insufficient to convincingly support the mechanistic claims being made.

I also find the authors' interpretation of a potential hormetic effect of carbonyls unclear and unconvincing. If hormesis were indeed occurring, one would expect beneficial effects associated with the small increases observed in the knockout (KO) model. The manuscript does not provide a clear rationale for the interpretation presented.

The central hypothesis of the manuscript is that arginine levels decrease due to carbonyl-mediated modifications. However, based on the response to my Question 2, it appears that the observed decrease in arginine can be explained by multiple metabolic pathways unrelated to arginine modification. It therefore seems likely that arginine modification by carbonyls contributes only minimally to the observed decline in arginine levels.

Another unresolved issue concerns skeletal muscle. It remains puzzling that little to no effect is observed in muscle tissue, despite the fact that glucose metabolism predominantly occurs there. The explanation provided in response to Question 3 is not convincing and does not adequately resolve this discrepancy.

In Question 4, I asked about the detection of nitric oxide. It remains unclear why NO cannot be measured directly using the Griess reaction.

Finally, in Question 5 I requested a more thorough characterization of renal pathology using additional histological, ultrastructural, and molecular analyses. However, the authors again present only PCR-based data. This does not adequately address the request for deeper pathological characterization.

Reviewer #2

(Remarks to the Author)
The authors have satisfactory answered to my points

Reviewer #3

(Remarks to the Author)
The authors have satisfactorily addressed my concerns.

Version 2:

Reviewer comments:

Reviewer #2

(Remarks to the Author)
I have gone through the responses to reviewer 1, and in my opinion the authors have throughly answered to all main points, which were valid and improved the manuscript. I agree the reviewer 1 (and the authors) that this study is restricted to (or focused on) certain enzymes and not the whole network of enzymatic systems, which I also eluded to earlier given that the double knockout is not generating a synergistic genetic interaction. The authors have now adjusted the text, added a series of high quality experiments, and justified their claims in a way that better reflect the compiled findings presented. Overall, this is a well preformed study that will add important information to the present literature.

Open Access This Peer Review File is licensed under a Creative Commons Attribution 4.0 International License, which permits use, sharing, adaptation, distribution and reproduction in any medium or format, as long as you give appropriate credit to the original author(s) and the source, provide a link to the Creative Commons license, and indicate if changes were made.
In cases where reviewers are anonymous, credit should be given to 'Anonymous Referee' and the source.
The images or other third party material in this Peer Review File are included in the article's Creative Commons license, unless indicated otherwise in a credit line to the material. If material is not included in the article's Creative Commons license and your intended use is not permitted by statutory regulation or exceeds the permitted use, you will need to obtain permission directly from the copyright holder.
To view a copy of this license, visit https://creativecommons.org/licenses/by/4.0/

REVIEWER COMMENTS

Reviewer #1 (Remarks to the Author):

Review Comments

The study by Professor Kroll and collaborators is well executed, and the dataset is compelling. The authors investigate how arginine modifications caused by methylglyoxal (MGO) and acrolein affect glucose metabolism, insulin signaling, and organ pathology particularly in the kidney using glyoxalase 1 (glo1) and aldo-keto reductase 1a1a (akr1a1a) double-knockout (DKO) zebrafish. The data show that combined loss of these enzymes leads to accumulation of MGO and acrolein, suppression of arginine metabolism, downregulation of insulin signaling, elevated whole-body glucose, postprandial hyperglycemia, and glomerular basement membrane thickening. Arginine supplementation restores Akt phosphorylation, improves insulin sensitivity, and alleviates renal pathology. These findings identify Glo1 and Akr1a1a as cooperative regulators of carbonyl detoxification and metabolic homeostasis.

Several major issues require clarification or additional data:

Reply:

We would like to thank the reviewer for positive and very encouraging comments on our manuscript.

1. Physiological relevance of the DKO model and carbonyl accumulation

The increases in MGO, MGO-derived AGEs, and acrolein in the DKO appear to be modest. This raises questions about how critical Glo1 and Akr1a1a truly are as a carbonyl-detoxifying system in vivo.

• How do the authors reconcile the relatively small changes in carbonyl stress markers with the substantial metabolic and signaling abnormalities observed?

• Do physiological or compensatory pathways buffer carbonyl accumulation in zebrafish, thereby limiting the magnitude of MGO/acrolein elevation?

Clarifying these points is essential for interpreting the broader relevance of the model.

Reply:

Small and additive changes in MG and ACR lead to MG-H1 accumulation, which depletes arginine levels further. Thus, the data indicate an amplified metabolic and signaling response driven by a persistent rather than transient increase in MG and ACR, as is often shown in other studies. Furthermore, the short half-life and high reactivity of MG result in the rapid and preferential modification of arginine/lysine residues to form relatively stable AGEs (commonly MG-H1). This suggests a downstream reaction rather than peak MG levels, which provides a plausible explanation for the modest elevation observed despite the pronounced functional effects. Notably, MG has been reported to exert a hormetic effect, displaying a non-linear response to increased levels of MG. Low concentrations of MG are known to initiate transcriptional responses that reduce MG production, enhance detoxification and activate repair pathways. This may also explain why modest increases in MG can result in significant functional effects (DOIs: 10.3390/antiox9100939 and 10.1152/physrev.00001.2019). Additionally, emerging evidence shows that, like MG, low-dose acrolein activates the Nrf2 pathway and other transcriptional stress-response pathways (DOIs: 10.1016/j.bbrc.2014.03.081 and 10.1016/S0378-4274(02)00055-3).

We also hypothesize that compensatory mechanisms play a major role, particularly in the single glo1 mutant. Therefore, the central focus of our laboratory and this manuscript is to identify these mechanisms. To provide additional evidence for our concepts and address the reviewer's concerns experimentally, we analyzed the expression of various aldhs and akrs by qPCR in DKO kidneys and DKO eyes (Figure 1 of this letter). As can be seen below, we found alterations in the expression of different aldh and akr genes in DKO kidneys: aldh3a1 was downregulated, aldh3a2a, aldh9a1b, akr7a3 were upregulated and aldh3a2b, aldh3b1, akr1a1b, akr1b1.1 were unaltered. In DKO eyes, the mRNA expression levels of aldh3a1, aldh3a2a, aldh9a1b and akr7a3 were upregulated, akr1b1.1 was downregulated and aldh3a2b, aldh3b1 and akr1a1b were unchanged (please refer to manuscript Figure S9A-H and in the text, page 7, 11). In summary, the data indicate that the different enzyme systems are interconnected in a complex and partially redundant manner in different organs, with their combined action limiting the extent to which MGO and acrolein levels may increase.

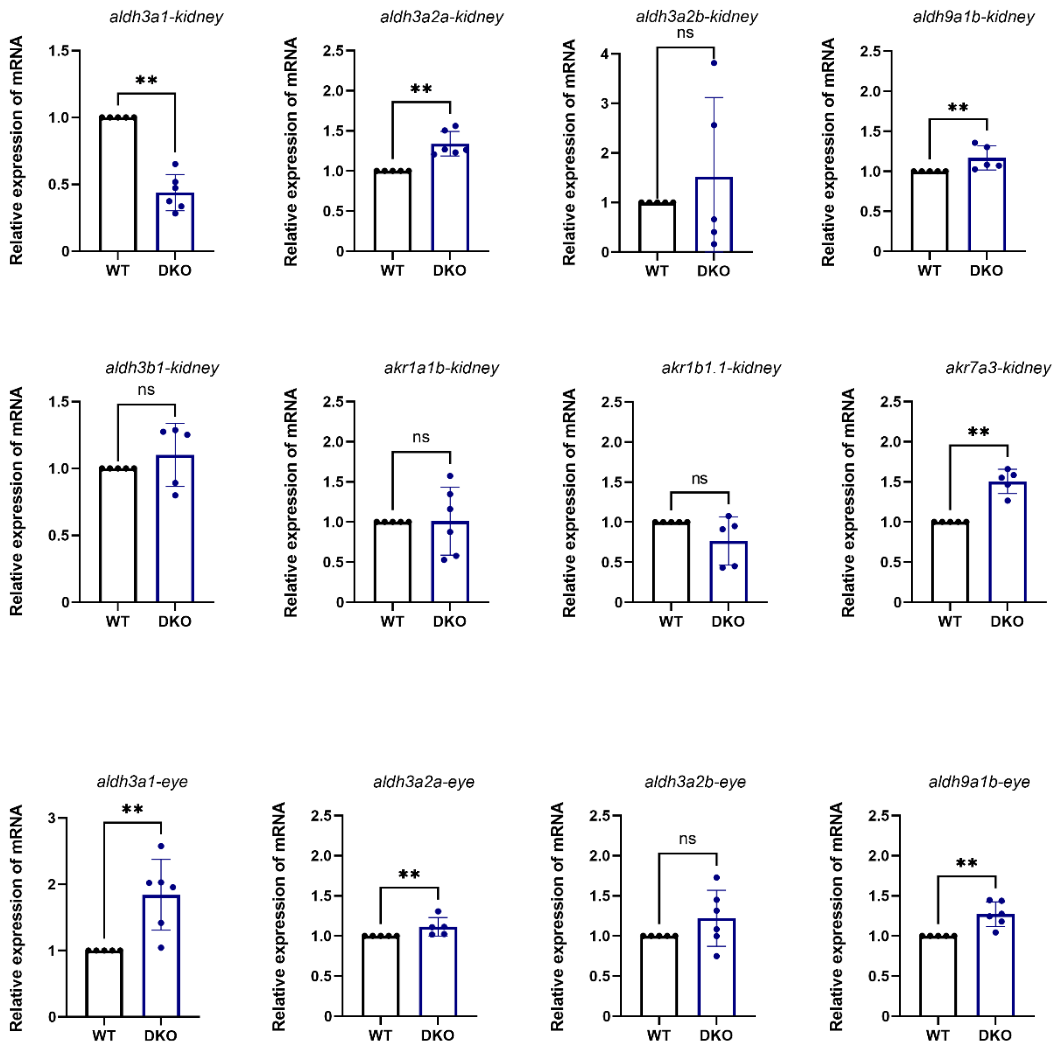

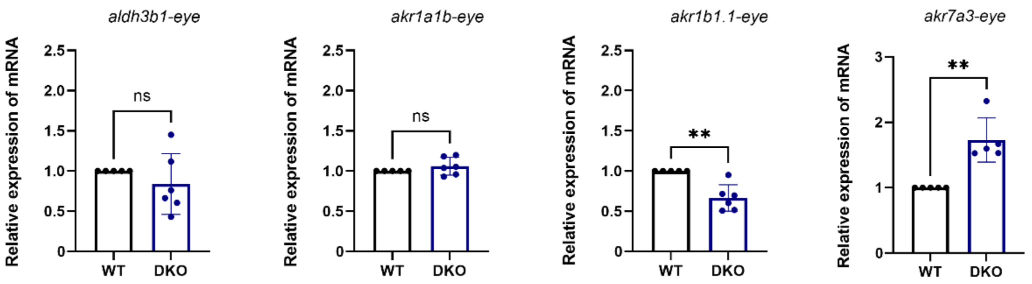

Figure 1: Expression analysis by RT-qPCR of different *aldh* and *akr* genes in DKO zebrafish kidney and eyes. mRNA expression levels were quantified by RT-qPCR and normalized to *arnt 2*. The bars indicate mean±SD values, n = 5-6. Statistical analysis was applied by Student's t-test. ns, not significant, $**p < 0.01$. arnt2, aryl hydrocarbon receptor nuclear translocator 2.

2. Discrepancy between small increases in MGO/acrolein and large decreases in arginine

The reduction in arginine is striking compared with the mild elevation in carbonyls and carbonyl-derived AGEs. This discrepancy needs mechanistic clarification to understand causality.

• How do the authors explain this disproportionate drop in arginine?

• Could increased arginine consumption via stress-activated pathways, altered urea-cycle metabolism, or secondary effects on amino-acid transporters contribute more to the phenotype than carbonyl modifications themselves?

Reply:

As discussed in the previous comment, our data indicate an amplified metabolic and signaling response, driven by a persistent increase in MG and ACR which explains the drop in arginine. Following the reviewer's suggestion, we performed several additional mRNA expression studies using qPCR (Figure 2 of this letter): 1. For stress-activated and -responsive pathways, we analyzed mapk8b (JNK), p38 MAPK, nfe2l2a, atf4a, and atf4b in adult DKO kidneys and DKO larvae, and found increased expression of mapk8b, atf4a and atf4b in both DKO larvae and DKO kidneys; 2. For urea-cycle metabolism, we analyzed the expression levels of ass1, asl, arg1 and odc1 by qPCR, and found altered levels of ass1 and asl in DKO kidneys and DKO larvae, and a decrease of odc1 only in DKO larvae (please refer to Figure S6A-I of the manuscript and in the text, page 7). For arginine metabolism and amino-acid transporters, we analyzed slc7a1a and slc7a1b, and found no alterations. The data suggest an additional contribution of the stress-activated pathways and urea-cycle metabolism to the increased arginine consumption and the data are in line with the decreased levels of arginine-related metabolites in DKO larvae (Suppl. Figure 3 of the manuscript).

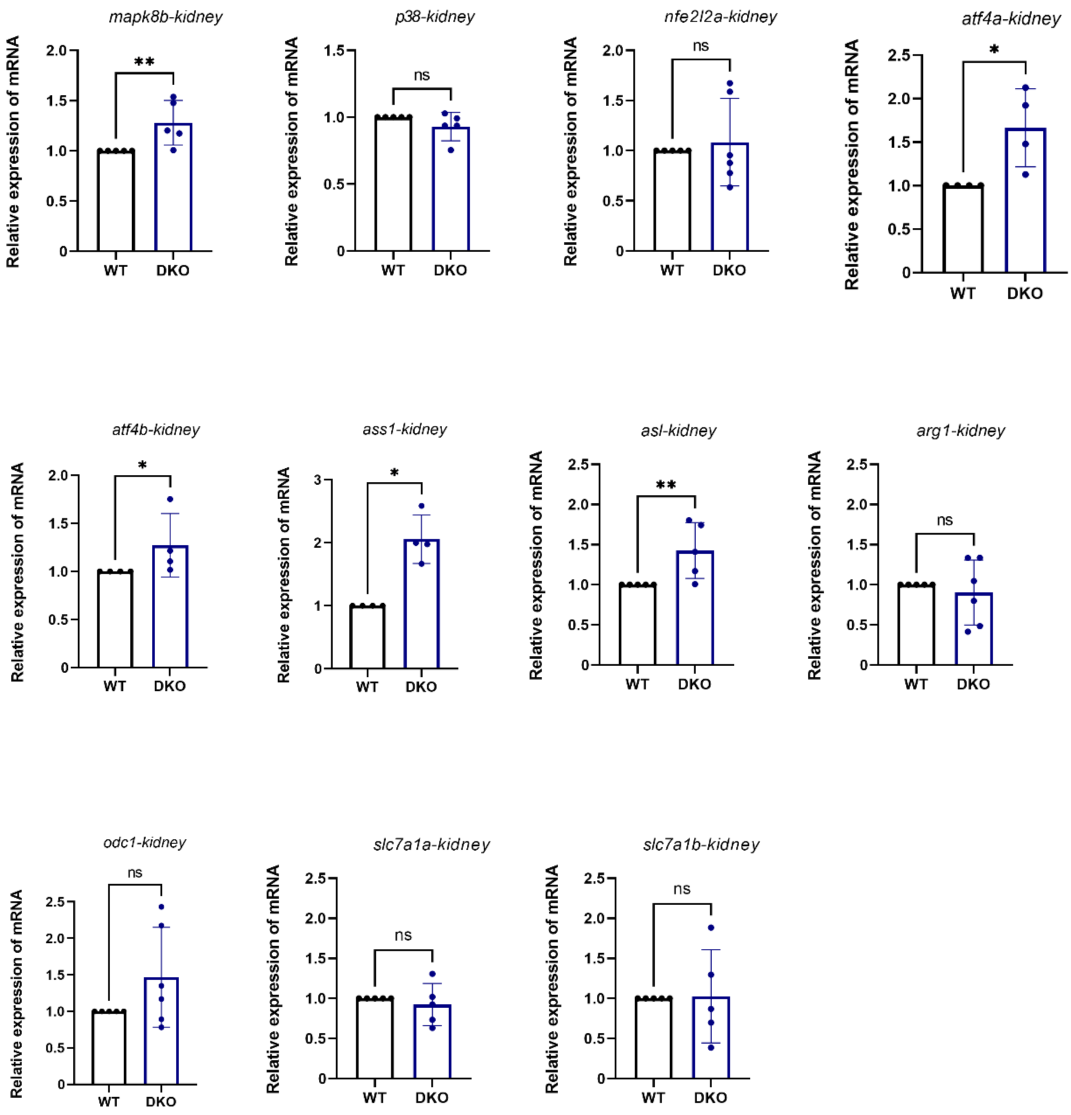
mapk8b-kidney
p38-kidney
nfe2l2a-kidney
atf4a-kidney
atf4b-kidney
ass1-kidney
asl-kidney
arg1-kidney
odc1-kidney
slc7a1a-kidney
slc7a1b-kidney
Relative expression of mRNA
WT
DKO
**
*
ns

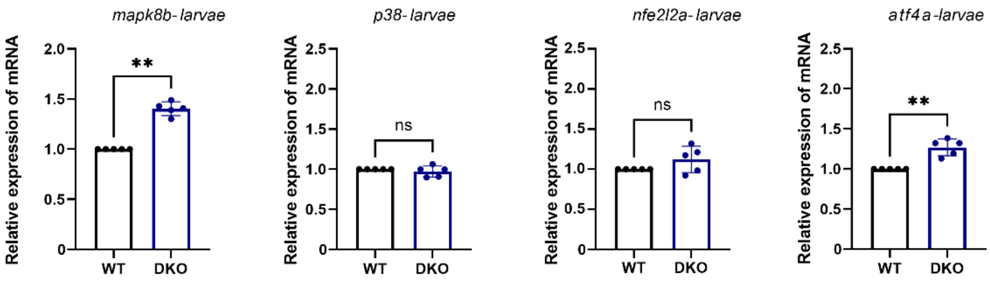
mapk8b-larvae
p38-larvae
nfe2l2a-larvae
atf4a-larvae
Relative expression of mRNA
WT
DKO
**
ns

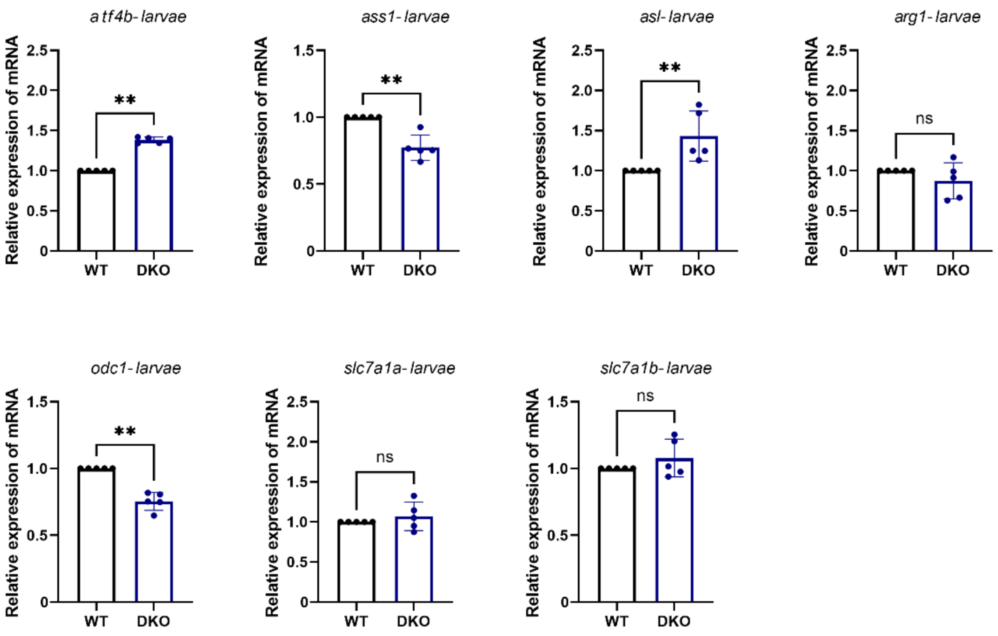

Figure 2: qPCR analysis of different genes from stress-activated pathways, urea-cycle metabolism and arginine metabolism in DKO zebrafish larvae and DKO kidneys. mRNA expression levels were quantified by RT-qPCR and normalized to *arnt 2*. The bars indicate mean±SD values, n = 4-6. Statistical analysis was applied by Student's t-test. ns, not significant, $*p < 0.05$, $**p < 0.01$. arnt2, aryl hydrocarbon receptor nuclear translocator 2.

3. Tissue-specificity: pronounced arginine depletion in liver but not muscle

Arginine depletion is dramatic in the liver yet minimal/not in muscle. Given that approximately 80% of glucose disposal occurs in skeletal muscle:

• How do the authors interpret normal arginine levels in muscle in the context of insulin resistance and glucose intolerance?

• Would muscle RNA-seq and/or RT-qPCR of key insulin-signaling, glucose-transport, and amino-acid-metabolism genes help explain why muscle appears spared in terms of arginine depletion but not in terms of systemic glycemic control? Such analyses are important for aligning tissue-level findings with whole-body metabolic phenotypes.

Reply:

Glo1 and, in particular, Akr1a1a exhibit high expression levels in the liver. In fact, we recently demonstrated that akr1a1a expression is over 200 times higher in the liver than in the other analyzed organs, including muscle (see Figures 1C and 1D in DOI: 10.1002/advs.202101281). Therefore, we expected and found the strongest phenotype in the liver of the DKO, but not in muscle. To provide more experimental data about the DKO muscle, we have performed additional qPCRs (Figure 3 of this letter) on insulin signaling (ins=insulin, insra=insulin receptor a, insrb=insulin receptor b), glucose transporters (glut2, glut8) and urea cycle/amino acid metabolism (ass1, asl, arg1, odc1). As can be seen below, the data suggest minor changes in glucose metabolism of DKO muscles (reduced insulin receptor a and glut8 expression), while the amino-acid-metabolism in DKO muscle appears unaltered. While, in the

liver, the expression levels of amino-acid-metabolism markers (ass1, asl) are downregulated, the arginine transporters are complementary upregulated (please refer to manuscript Figure4A-F, Figure S3C-F and in the text, page 5-6).

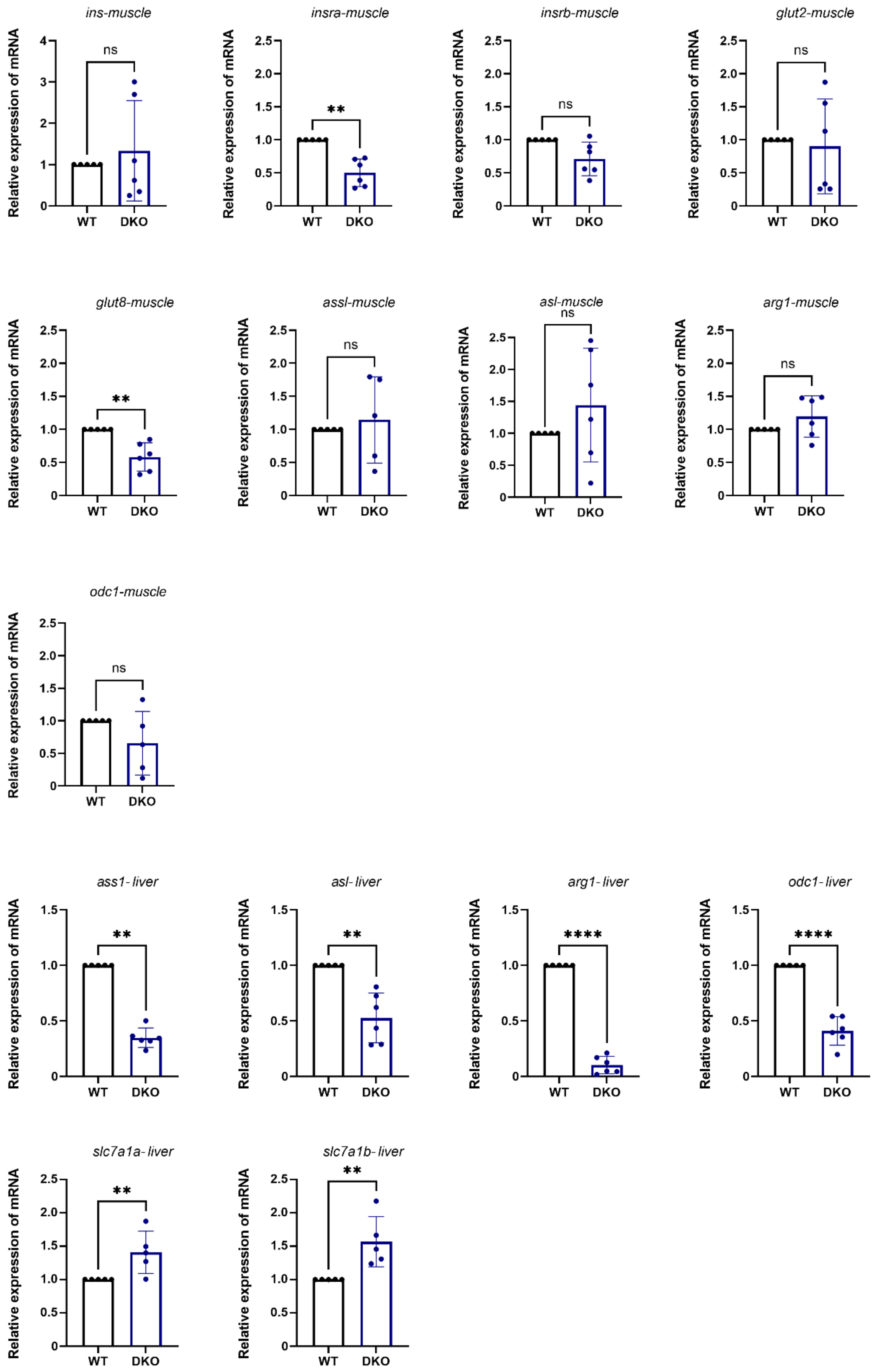

Figure 3: qPCR analysis of different genes from insulin metabolism and amino acids metabolism in DKO zebrafish muscle and liver tissues. mRNA expression levels were quantified by RT-qPCR and normalized to *arnt 2*. The bars indicate mean±SD values, n = 5-6. Statistical analysis was applied by Student's t-test. ns, not significant, **$p < 0.01$. arnt2, aryl hydrocarbon receptor nuclear translocator 2.

4. Potential involvement of the nitric oxide (NO) system

Given the profound reduction in arginine, a substrate for NO synthesis, there may be altered endothelial NO production and vascular dysfunction.

• Can the authors measure vascular NO pathway components (NO levels, NOS activity/expression, nitrite/nitrate, endothelial function assays)?

• Could impaired NO-mediated vasodilation contribute to the metabolic and renal pathologies observed in the DKO?

Reply:

Measuring nitrite levels using commercial kits and analyzing NO by DAF staining in zebrafish is very challenging and can lead to misleading interpretations (DOI: 10.1089/zeb.2012.0746; doi:10.2754/avb201180030309 and doi.org/10.3390/biom13121780). Yet, to address the reviewer's questions, we analyzed nitric oxides enzymes expression. Notably, the identity and function of zebrafish NOS enzymes are only partially characterized, and they are not identical to human eNOS (NOS3), iNOS (NOS2) and nNOS (NOS1) enzymes. Specifically, the function of zebrafish nos1 is unknown, zebrafish nos2a is likely homologous to human iNOS, and zebrafish nos2b is probably homologous to human eNOS. A distinct nos3 gene has not yet been found in the zebrafish genome (DOI:10.2174/1570161116666181031101413).

To experimentally address the reviewer's concerns, we first analyzed the expression of nos2a and nos2b by qPCR in 4 dpf DKO and wild-type larvae and found that the mRNA expression levels of nos2a was upregulated, while nos2b was downregulated (Figure 4 of this letter). As an additional approach, we applied the NO donor sodium nitroprusside (SNP) to DKO zebrafish larvae and measured subsequently glucose at 4 dpf (Figure 5 of this letter), and found that SNP could decrease the glucose levels in DKO larvae (please refer in the manuscript to Figure S11A-C and the text, page 8). Lastly, we used Tg(fli:wt1b) larvae microinjected with the pdx1 morpholino (hyperglycemia model) and combined SNP incubation and analyzed pronephros at 2 dpf. As can be seen in Figure 6 of this letter, the glomerular length in pdx1-MO larvae with SNP treatment was decreased, and the pronephric neck length in pdx1-MO larvae with SNP treatment was increased compared to pdx1-MO larvae, which demonstrates the role of NO in renal alterations. We have added the data in Figure 7D-F of the manuscript and on page 8-9 accordingly.

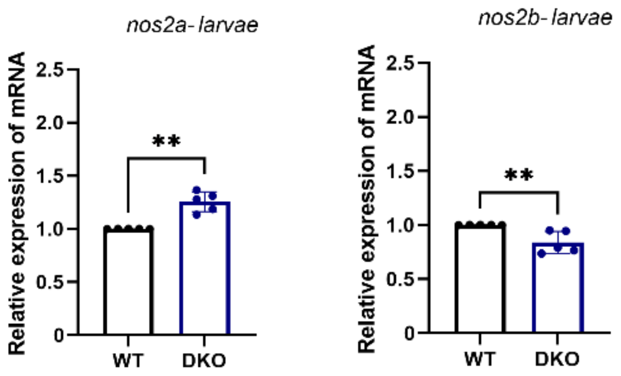

Figure 4: mRNA expression levels were quantified by RT-qPCR and normalized to *arnt 2*. The bars indicate mean ± SD values, n = 5. Statistical analysis was performed by Student's t-test. **p < 0.01. arnt2, aryl hydrocarbon receptor nuclear translocator 2.

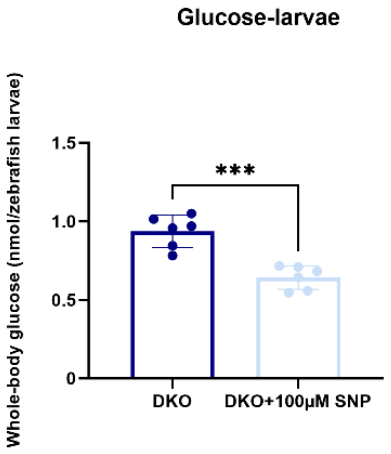

Figure 5: Sodium nitroprusside (SNP) lowers glucose in DKO larvae. The bars indicate mean ± SD values, n = 6. Statistical analysis was performed by Student's t-test. ***p < 0.001. SNP, sodium nitroprusside.

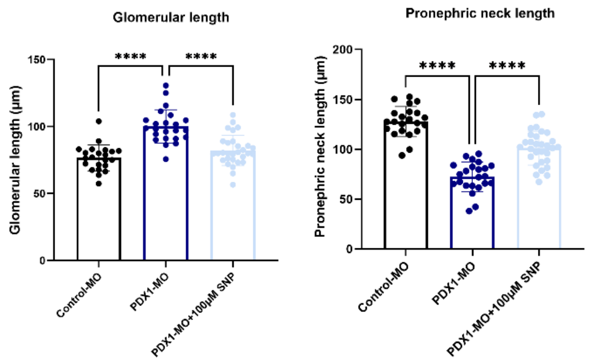

Figure 6: Sodium nitroprusside (SNP) rescued pronephros alterations in hyperglycemic zebrafish (pdx1 knock down) embryos. The bars indicate mean ± SD values, n = 22-30. Statistical analysis was performed by one-way ANOVA. ****p < 0.0001. SNP, sodium nitroprusside.

5. Limited characterization of renal pathology

While glomerular basement membrane thickening is shown, renal pathology is not explored in depth.

• Additional histological, ultrastructural, and molecular analyses (e.g., podocyte markers, ECM remodeling, oxidative stress, or inflammatory pathways) would strengthen the link between carbonyl stress, arginine depletion, and kidney damage.

Reply:

To follow the reviewer's suggestion, we performed qPCRs of ECM remodeling, podocyte markers, and inflammatory factors in adult DKO and WT kidneys. As can be seen in Figure 7 of this letter, the expression levels of col4a3 (collagen, type IV, alpha 3), col4a4 (collagen, type IV, alpha 4), col4a5 (collagen, type IV, alpha 5), lama1 (laminin subunit alpha-1), lamb1a (laminin subunit beta-1a), csf1b (colony stimulating factor 1b) and ifng1 (interferon gamma 1) were significantly upregulated, while nephrin, podocin, IL-1β, IL-6 were unchanged, which suggest that the carbonyl stress in DKO kidney is associated with extracellular matrix remodeling and the activation of inflammatory response. The data align with observations of human kidneys with diabetic nephropathy, in which glomerular basement membrane thickening is primarily driven by increased collagen IV and laminin deposition (doi.org/10.1093/ndt/gfad052 and 10.1038/modpathol.2011.216). We have added some results in Figure 5F-J, Figure S6A-I (see your comment 2) of the manuscript and added an explanation on page 7.

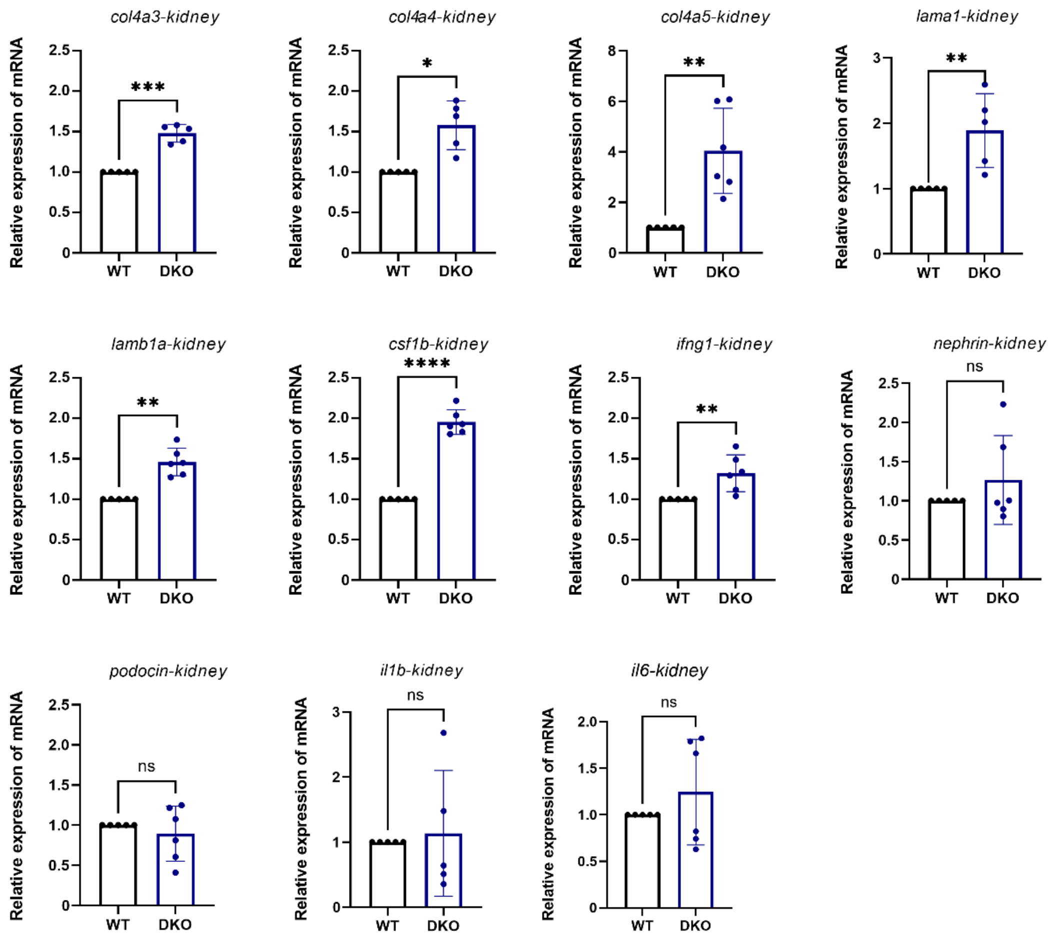

Figure 7: mRNA expression levels were quantified by RT-qPCR and normalized to *arnt 2*. The bars indicate mean±SD values, n = 5-6. Statistical analysis was applied by Student's t-test. ns, not significant, *p < 0.05, **p < 0.01, ****p < 0.0001. arnt2, aryl hydrocarbon receptor nuclear translocator 2.

6. Interpretation of arginine supplementation effects

Although arginine is reduced in the DKO, the absolute concentration remains relatively high compared with the amounts typically needed for NO-dependent signaling. A deeper mechanistic interpretation of the rescue effect is important.

• How do the authors reconcile this with the strong phenotypic rescue by arginine supplementation?

• Do supplemented arginine levels fully normalize physiological concentrations in relevant tissues?

Reply:

The absolute concentration of arginine does not necessarily correlate with NOS activity. Some time ago, the 'L-arginine paradox' was observed, stating that the intracellular L-arginine concentration does not determine NO production in endothelial cells (doi.org/10.1016/j.bbrc.2011.09.112). The authors of the study concluded that 'when ARG is delivered inside the cell without transporter involvement, little eNOS activity can be detected. Thus, the intracellular concentration of ARG may be irrelevant to the cellular production of NO through eNOS'. Therefore, in addition to the absolute concentration of arginine, NOS activity may depend on local arginine pools within cells, competition between NOS and arginase II, the activity of arginine transporters, and the presence of endogenous NOS inhibitors such as ADMA (asymmetric dimethyl arginine). To experimentally address the reviewer's concern, we measured internal arginine levels in zebrafish after external arginine incubation in DKO larvae treated with arginine (Figure 8 of this letter). As can be seen below the arginine incubation in DKO larvae fully normalized physiological arginine concentrations to wildtype levels. We have added these data in Figure S10C and modified manuscript on page 8 accordingly.

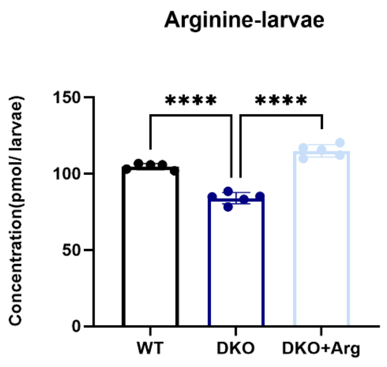

Figure 8: Arginine supplementation normalizes arginine concentration in DKO zebrafish larvae. The bars indicate mean ± SD values, n = 5. Statistical analysis was performed by one-way ANOVA. ****p < 0.0001. Arg, L-arginine.

Overall

This work addresses an important gap in understanding carbonyl stress, arginine metabolism, and metabolic homeostasis. However, the issues above should be addressed to clarify mechanistic relationships, strengthen physiological relevance, and ensure that conclusions are well supported by tissue-specific data.

Reply:

We would like to thank the reviewer for positive and very encouraging comments on our manuscript.

Reviewer #2 (Remarks to the Author):

This study explores the additive/synergistic roles of GLO1 and AKR1A1A in detoxifying reactive carbonyl species. There is generally not so much of a synergistic genetic interaction, but more additive effects of their two previously published mutants (reference 9 and 33) – which limits the novelty. However, to add novelty and strengthen the reliability of the conclusions, further elaboration and experimental verification are required regarding the specific mechanisms of arginine depletion, the nature of the insulin signaling impairment (secretion defect / reduced sensitivity), and the observed organ-specific phenotypes (kidney / retina).

Reply:

We would like to thank the reviewer for positive comments on our manuscript.

Major Comments

Comment 1. The authors propose that the accumulation of MG and ACR leads to arginine depletion through the formation of adducts (such as MG-H1). While Figure 2 demonstrates a decrease in free arginine and an increase in MG-H1, this correlation is insufficient to prove that the depletion is solely due to scavenging or consumption by RCS. It is crucial to rule out impairments in arginine biosynthesis or transport. Although Figure 4 shows a downregulation of insulin-related genes, did the authors assess the expression levels of arginine biosynthetic enzymes (such as ass1, asl) or cationic amino acid transporters (such as slc7a family)?

Reply:

Following the reviewer's suggestion, we have analyzed expression levels of arginine biosynthetic enzymes and cationic amino acid transporters by qPCR in DKO larvae. As can be seen in below (Figure 9 of this letter) for urea-cycle metabolism, we found altered levels of ass1, asl, odc1 in DKO larvae, but arg1 and amino-acid transporters were unchanged.

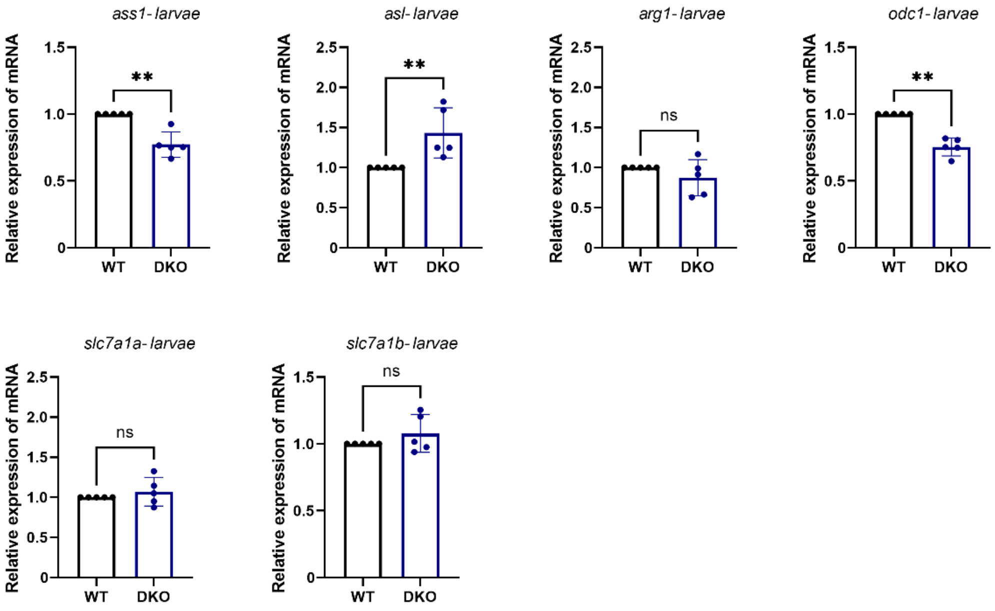

Figure 9: Expression levels (qPCR) of arginine biosynthetic enzymes and cationic amino acid transporters in DKO larvae. mRNA expression levels were quantified by RT-qPCR and normalized to *arnt 2*. The bars indicate mean±SD values, n = 5. Statistical analysis was applied by Student's t-test. ns, not significant, **p < 0.01. arnt2, aryl hydrocarbon receptor nuclear translocator 2.

Comment 2. The conclusion states that arginine supplementation improves insulin sensitivity. However, the data show a significant reduction in ins mRNA in DKO larvae, suggesting potential beta-cell dysfunction or reduced beta-cell mass, whereas reduced pAKT indicates impaired signaling. Does arginine function by promoting insulin secretion from beta-cells or by enhancing peripheral insulin sensitivity? Since the Western Blot data are derived from whole-fish lysates, distinguishing between these sources is difficult. The authors should clarify this distinction or discuss it in greater depth.

Reply:

We observed reduced levels of insulin, as well as reduced expression of insulin receptor a and b, and reduced pAKT activity in DKO mutants. These findings are consistent with the pathogenic mechanisms of the formation of type 2 diabetes, whereby reduced peripheral insulin sensitivity (or insulin resistance) in the early disease stage can be compensated for by increased insulin expression and secretion. However, at later stages, the pancreas fails to meet the high demand for insulin, ultimately resulting in beta cell exhaustion. Although, reports have shown that arginine can improve peripheral insulin sensitivity and enhance insulin secretion (for example, DOI: 10.1186/s12933-023-01827-2 and DOI: 10.1530/eje.0.1400087), we cannot definitively distinguish whether these changes reflect improved β-cell function, enhanced insulin secretion, or increased peripheral insulin sensitivity in this case. We have therefore revised the manuscript to clarify this limitation and discuss both possibilities in greater depth, please refer to page 10.

Comment 3. Figure 5 and Supplemental Figure 7 indicate that the DKO model exhibits distinct glomerular basement membrane thickening, yet the retinal vasculature remains unaffected. The authors speculate on the existence of retina-specific protective mechanisms, but empirical data are lacking. It is recommended to assess differences in MG and ACR levels between retinal and renal tissues in adult DKO zebrafish, or to compare the expression of antioxidant enzymes (such as ALDHs or other AKRs) via qPCR.

Reply:

To address the reviewer's concerns, we analyzed the expression of various aldhs and akrs by qPCR in kidneys and eyes. As can be seen below (Figure 10 of this letter), we found in DKO kidneys aldh and akr genes which were downregulated (aldh3a1), upregulated (aldh3a2a, aldh9a1b, akr7a3) and unaltered (aldh3a2b, aldh3b1, akr1a1b, akr1b1.1). In DKO eyes, aldh3a1, aldh3a2a, aldh9a1b and akr7a3 were upregulated, akr1b1.1 was downregulated and aldh3a2b, aldh3b1 and akr1a1b were unchanged. In summary, the data show that the different enzyme systems are interconnected, with their combined action limiting the extent to which MGO and acrolein levels may increase. They also demonstrate tissue-specific alterations that may explain why certain organs are more susceptible to damage, while others are protected. We have added the data for eyes in Figure S9A-H and additional description in manuscript on page 7, 11.

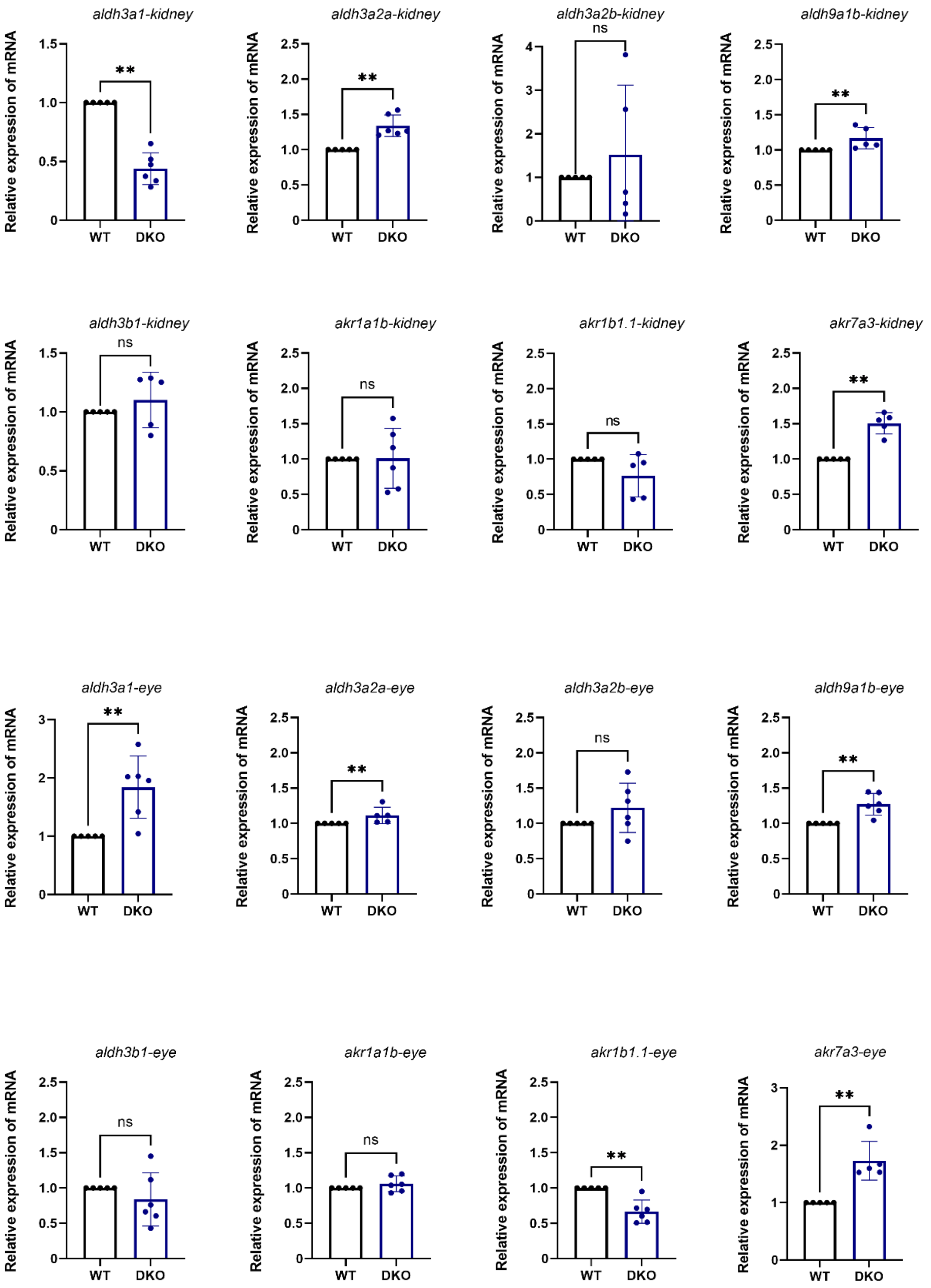

Figure 10: Expression analysis by qPCR of different *aldh* and *akr* genes in DKO zebrafish kidney and eyes. mRNA expression levels were quantified by RT-qPCR and normalized to *arnt 2*. The bars indicate mean±SD values, n = 5-6. Statistical analysis was applied by Student's t-test. ns, not significant, **p < 0.01. arnt2, aryl hydrocarbon receptor nuclear translocator 2.

Comment 4. In Figure 6E, L-NAME is used to demonstrate that the effect of arginine is dependent on the NO pathway. However, the experiment lacks a critical control: does treatment with L-NAME alone (without arginine supplementation) induce hyperglycemia in the WT or DKO groups? If L-NAME exerts a hyperglycemic effect independent of the arginine rescue pathway, the specificity of the conclusion is compromised. It is necessary to provide data for WT + L-NAME or to discuss the basal metabolic effects of L-NAME.

Reply:

As recommended by the reviewer, we measured glucose levels in the following experimental groups: wildtype larvae, wildtype larvae + L-NAME, DKO larvae and DKO larvae + L-NAME. As shown in the Figure 11 of this letter, L-NAME treatment alone does not change glucose concentrations. We have added the data in Figure S12D-E and additional explanation in manuscript on page 8.

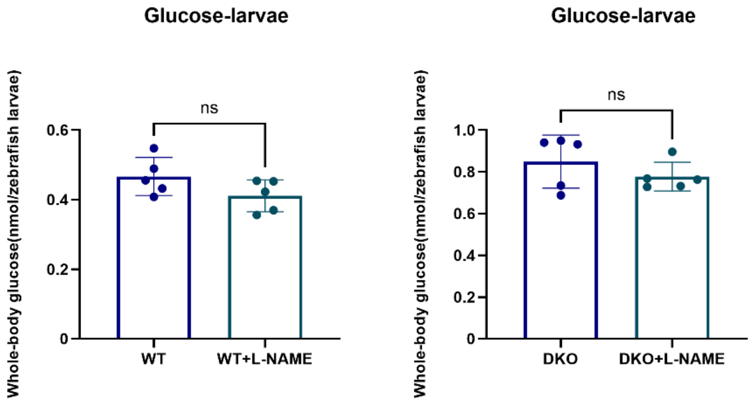

Figure 11: Glucose concentrations are not altered upon L-NAME incubation. The bars indicate mean ± SD values, n = 5. Statistical analysis was performed by Student's t-test. ns, not significant. L-NAME, N(ω)-nitro-L-arginine methyl ester.

Comment 5. Although arginine supplementation restored pAKT levels and lowered glucose, its impact on in vivo MG-H1 levels remains unclear. Does arginine mechanistically reduce protein modification by competitively scavenging RCS, or does it merely serve as a substrate to promote NO synthesis regardless of RCS levels? It is recommended to measure MG-H1 levels following arginine supplementation to clarify this mechanism.

Reply:

Following the reviewer's recommendation, we measured MG-H1 levels in DKO larvae treated with arginine supplementation. The new data show (Figure 12 of this letter) that MG-H1 levels are not altered in DKO group treated with arginine, suggesting that arginine does not mechanistically reduce protein modification, but rather serve as a substrate to promote NO synthesis regardless of RCS levels.

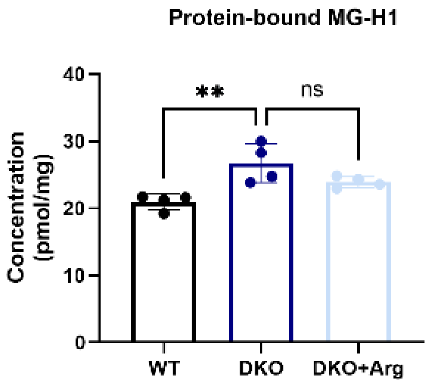

Figure 12: MG-H1 levels are not altered after arginine incubation in DKO groups. The bars indicate mean ± SD values, n = 4. Statistical analysis was performed by one-way ANOVA. ns, not significant, **p < 0.01.

Minor comments:

• There is a need for a scheme of the metabolite system for glo1 and ark1a1a with their subtract and how they interact

Reply:

We have now added a graphical abstract. Please refer to the graphical abstract.

• In fig3-A the authors compare DKO with other and conclude that insulin signaling pathway in GLO1/AKR1A1A DKO is downregulated in comparison to WT, but it appears like it is already the case for single KO versus DKO. Similar point for fig4, most gene expression looks down in single KO as well? is it significant?

Reply:

We agree that, based on the heatmap visualization, insulin signaling pathway appears reduced not only in the DKO group but also in the single KO groups compared to WT. To clarify this point, we extended the analysis of Fig. 3A by performing pairwise GSVA analysis for the insulin signaling pathway across all groups. The results are provided in the table below (Table 1 of this letter).

Although both single KO groups (AKR1A1A KO and GLO1 KO) show a trend toward reduced insulin signaling compared to WT, these differences do not reach statistical significance after multiple testing correction (adjusted p values = 0.0788 and 0.2080, respectively). In contrast, the DKO group exhibits a pronounced and statistically significant downregulation of insulin signaling compared to WT (logFC = −0.4223, adjusted p value = 0.0003). Consistent with the GSVA results, qPCR validation of selected genes in this pathway (Figure 4G and 4I) also shows the lowest mRNA expression levels in the DKO group. Taken together, these results indicate that while partial suppression of insulin signaling may already be present in single KO conditions, the combined loss of GLO1 and AKR1A1A leads to the most robust and statistically significant impairment of insulin signaling.

	GSVA analysis: insulin signaling pathway					
	logFC	***AveExpr***	***t***	***P.Value***	***adj.P.Val***	***B***
DKO vs WT	*-0.4223*	*-0.0421*	*-5.8258*	*0.0001*	**0.0003**	*1.1264*
AKR1A1A.KO vs WT	*-0.2843*	*0.0398*	*-2.5627*	*0.0220*	*0.0788*	*-3.4701*
GLO1.KO vs WT	*-0.1612*	*0.0884*	*-1.5200*	*0.1498*	*0.2080*	*-5.8631*
DKO vs GLO1.KO	*-0.2611*	*-0.1227*	*-3.0006*	*0.0095*	*0.0239*	*-3.2148*
DKO vs AKR1A1A.KO	*-0.1380*	*-0.1905*	*-1.6965*	*0.1142*	*0.1863*	*-5.8676*

Table 1: Statistical analysis of RNAseq. data.

• In the abstract authors use full length enzyme name and abbreviated one at random and without defined the abbreviation in the abstract

Reply:

We have removed all abbreviation from the abstract.

• typo for ark1a1a in line 92 (introduction)

Reply:

This has been modified accordingly

• In line 215 they use an undefined abbreviation (full name come later in the text)

Reply:

We have modified it to glomerular basement membrane.

• The authors could explain the reason they chose to use specific transgenic lines, such as Tg(fli1:EGFP)

Reply:

The Tg(fli1:EGFP) reporter line is widely used in the zebrafish community for visualizing the vasculature. We have added a short comment to the manuscript under "Material and methods - Zebrafish husbandry".

• In fig6-C the molecular marker size should be indicated instead of the approximative size of the band of interest

Reply:

This has been modified accordingly.

• The color of the bar graph should be change. The yellow bars are barely visible with white background.

Reply:

We have replaced the color of all figures.

• It would be beneficial to expand the discussion on why Glo1 single-knockout zebrafish exhibit only moderate MG elevation without severe hyperglycemia, in contrast to mammalian models where Glo1 knockout is often lethal or phenotypically severe. This comparison is important for understanding the differences of the zebrafish model.

Reply:

We are aware that the community believes that mammalian glo1 knockout animals are often lethal or phenotypically severe. However, this information is incorrect. Two different published glo1 KO mouse models have shown that the animals are viable and metabolically healthy (DOIs:10.1016/j.bbrc.2017.06.063 and 10.1016/j.molmet.2018.09.005). The data in mice are comparable to those for the zebrafish glo1 knockout. The Shafie et al. (2016) paper (DOI: 10.1042/BCJ20160691), which is often cited as proof of the embryonic lethality of glo1 deletion in mouse, does not demonstrate this. In fact, it shows that the IMKC/Lexicon 'glo1 knockout' mice were not truly glo1-deficient due to the presence of a duplicated wild-type glo1 allele in the embryonic stem cells used for targeting. Based on this finding and human genetic data, the authors inferred that complete loss of glo1 would be subject to strong negative selection, likely due to excessive methylglyoxal accumulation. Therefore, the statement about lethality is an interpretation rather than a direct experimental finding. Subsequent studies, including genuine glo1 knockout mouse models, have since demonstrated that complete loss of glo1 is compatible with viability. In this sense, the earlier interpretation has persisted in the literature to some extent, even though the experimental evidence has evolved. We are aware of this misinterpretation within the field and have recently summarized this issue, alongside other limitations and perspectives, in a review article in Cardiovascular Diabetology (Li S, Nawroth PP and Kroll J. Reactive carbonyl species in health and chronic disease – from methylglyoxal to an integrative network of metabolic regulation. Cardiovasc Diabetol. 2026; in press). This manuscript has now been accepted and added to the references list of the manuscript (page 4).

Reviewer #3 (Remarks to the Author):

The manuscript by Li et al shows that increased MG and ACR in glo1/akr1a1a double knockout (DKO) zebrafish leads to hyperglycemia and diabetic nephropathy but not retinopathy. Transcriptomic analysis revealed impaired insulin and MAPK signaling and impaired arginine metabolism in the DKO fish. The authors demonstrate that decreased free arginine mediates the glucose dysregulation, as arginine supplementation corrects the molecular changes and hyperglycemia. They further identify NO synthesis as a mediator of the arginine effects. The manuscript is clearly written. The data are of good quality and support the conclusions. Nevertheless, several issues are identified.

Reply:

We would like to thank the reviewer for positive and very encouraging comments on our manuscript.

Major issue

1. The authors only used a non-selective NOS inhibitor. Since selective NOS inhibitors are available, it may be a good idea to use them to identify the NOS isoform(s) responsible for the arginine effect.

Reply:

Following the reviewer's suggestion, we repeated the experiments using the selective iNOS inhibitor 1400W dihydrochloride (a specific eNOS or nNOS inhibitor to the best of our knowledge does not exist), measuring glucose concentrations in DKO larvae that had been treated with arginine. As can be found in new figure below (Figure 13 of this letter) we found decreased glucose levels in 1400W treated larvae. The data suggest that inhibition of iNOS by 1400W increases eNOS activity due to higher arginine availability, which subsequently increases NO production and decreases glucose levels.

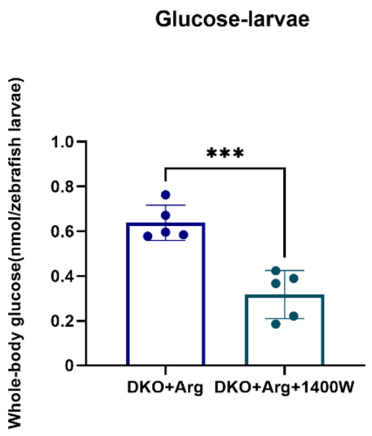

Figure 13: iNOS inhibitor 1400W reduces glucose levels in DKO larvae treated with arginine. The bars indicate mean ± SD values, n = 5. Statistical analysis was performed by Student’s t-test. ***$p < 0.001$. 1400W, iNOS inhibitor.

2. It is unclear how increased MG and ACR induce transcriptomic changes in genes related to arginine biosynthesis and metabolism. Is it possible to mine the RNA-seq data to identify candidate transcription factors that mediate the changes?

Reply:

Following the reviewers' suggestions, we mined the RNA-seq dataset and identified multiple transcription factors with established links directly and indirectly to amino acid biosynthesis and metabolism that are significantly altered see the below table (Table 2 of this letter). Notably, key metabolic and inflammatory regulators, including STAT3, NF-κB components (rela, nfkb1), PPARγ (pparg), and FOXO family members (foxo1b, foxo3b), are significantly downregulated. From qPCR analysis, the atf4a and atf4b are significantly upregulated. These coordinated changes are consistent with a transcriptional reprogramming that could underlie the observed suppression of the arginine and proline metabolism pathway.

Transcription factors (DKO vs WT)

Symbol	***logFC***	***adj.P.Val***	***regulated***	***Function***
nfe2l2a	*-0.5259453*	*0.00211551*	*normal*	*Anti-oxidative and detoxification regulation.*
stat1a	*0.28860169*	*0.00022588*	*normal*	*Anti-virus and Inflammation regulation.*
stat1b	***0.63321886***	***0.02460471***	***up***	*Anti-virus and Inflammation regulation.*
stat3	***-1.1809052***	***1.65E-06***	***down***	*Inflammation and proliferation regulation.*
rela	***-1.3979019***	***2.51E-06***	***down***	*Inflammation and immune response regulation.*
nfkb1	***-1.2623097***	***3.49E-05***	***down***	*Inflammation and immune response regulation.*
pparaa	*0.17958603*	*0.33889441*	*normal*	*Fatty acid and energy metabolism regulation.*
pparab	*0.07902975*	*0.66979681*	*normal*	*Lipid and energy metabolism regulation.*
pparg	***-2.2788667***	***0.00111851***	***down***	*Lipogenesis and insulin sensitivity regulation.*
hif1aa	*0.49169746*	*0.00467833*	*normal*	*Hypoxia sensing.*
hif1ab	*-0.1248084*	*0.00805457*	*normal*	*Hypoxia sensing.*
foxa2	*-0.3382841*	*0.01640003*	*normal*	*Development and metabolism regulation.*
cebpa	*-0.5625684*	*0.00067863*	*normal*	*Cell differentiation and metabolism regulation.*
cebpb	*-0.2792612*	*0.24847517*	*normal*	*Inflammation, metabolism regulation.*
myca	*0.19583118*	*0.00758266*	*normal*	*Cell proliferation, differentiation regulation.*
mycb	*0.133151*	*0.1296185*	*normal*	*Cell proliferation and metabolism regulation.*
klf2a	*-0.5159616*	*0.00150714*	*normal*	*Vascular development, endothelial homeostasis.*
klf2b	*-0.0714819*	*0.67063564*	*normal*	*Vascular development, endothelial homeostasis.*
klf4	***-0.704601***	***0.02168088***	***down***	*Cell differentiation, homeostasis regulation.*

foxo1a	*-0.4361323*	*0.00065491*	*normal*	*Insulin signaling, oxidative stress regulation.*
foxo1b	***-1.312031***	***0.0003803***	***down***	*Metabolism and oxidative stress regulation.*
foxo3b	***-0.689851***	***2.84E-07***	***down***	*Oxidative stress regulation.*
atf4a	***From qPCR***		***up***	*Amino acid metabolism and stress regulation.*
atf4b			***up***	*Amino acid metabolism and stress regulation.*

Table 2: Several transcription factors related to arginine metabolism are altered in DKO zebrafish.

3. Instead of using the pdx1 mutant fish the lab reported previously, the authors chose pdx1 morphants from a single morpholino antisense oligo for the arginine supplementation studies. Given the non-specific activity of morpholino oligos, it is recommended to use multiple oligos targeting the same gene and validate disruption of translation or splicing. Disregard this comment if the specificity and activity of the morpholino oligo have been validated previously.

Reply:

The specificity and activity of the morpholino oligo has been previously validated by us and by others (DOI:10.2337/db21-0645, DOI:10.2337/db19-0873 and DOI:10.1016/j.ygcen.2010.10.010). Importantly, the pdx1 morpholino generates the same results as the pdx1 mutant, e.g. hyperglycemia, increased angiogenesis in the eye vasculature, blood vessel vasodilation and glomerular hypertrophy in the pronephros. Consequently, the morpholino fully fulfils the criteria published by the consensus statement for the use of morpholinos (DOI: 10.1371/journal.pgen.1007000).

Minor issue:

It looks like the glut4 (line 205) should be glut2.

Reply:

This has been modified accordingly

REVIEWER COMMENTS

Reviewer #1 (Remarks to the Author):
Although the authors did address all the points of concern, several points of concern remain.

While the study is technically well executed, the observed differences are very small and their biological relevance remains questionable since carbonyl stress and detoxification involve a large network of enzymes. In this study, the authors selected two enzymes and investigated them in combination. Although these enzymes are likely relevant, they are certainly not the only ones involved. Multiple enzymatic systems interact in the detoxification of reactive carbonyl species, and this complexity is only superficially addressed. Indeed, the authors themselves acknowledge this interaction in their response to my Question 1.
Reply:
We thank the reviewer for raising this important point regarding the complexity of reactive carbonyl species detoxification networks. We fully agree that multiple enzymatic systems contribute to carbonyl stress defense and that this represents a highly interconnected and complex biological process. However, we would like to emphasize that the goal of the present study was not to provide a comprehensive systems-level analysis of all detoxification pathways, but rather to dissect the specific and combinatorial contribution of two key enzymes within this network. This reductionist approach allows us to establish causal relationships that would be difficult to resolve in more complex multi-enzyme settings. Importantly, the two enzymes investigated here were selected based on their well-established roles in reactive carbonyl detoxification and their predicted functional interplay (DOI: 10.1172/jci.insight.126154; 10.1002/advs.202101281). By analyzing their combined perturbation, we provide experimentally tractable insight into how specific nodes within the network contribute to carbonyl stress handling. We agree that additional pathways and enzymes are likely to modulate these effects. However, systematically addressing the full network complexity—e.g., through the generation and characterization of higher-order mutants—represents a substantial long-term effort that goes beyond the scope of a single study.

Several questions raised in the first review round are not addressed directly but rather indirectly through gene expression data. For example, the authors refer to transcriptional changes when discussing the modest increases in MGO and acrolein levels or alterations in NO concentrations. Such indirect evidence is insufficient to convincingly support the mechanistic claims being made.
Reply:
We thank the reviewer for this important comment. We agree that transcriptional changes alone are insufficient to establish mechanistic causality. However, we would like to clarify that our conclusions are based on a combination of direct biochemical measurements and functional perturbation approaches, rather than solely on gene expression data. Firstly, we directly quantified reactive carbonyl species and their downstream modifications, including both protein-bound and protein-free MG-H1 (Figs. 1A-B, 2F-G, S3B-C). This provides biochemical evidence for increased carbonyl stress. It is notable that the observed increases in MGO and acrolein are modest and fall within the physiological range reported in vivo and in patients. This is in contrast to the supraphysiological concentrations that are frequently used in vitro. Other reactive carbonyls remained normal (Fig. 1C-G). Thus, our data suggest that a modest but chronic increase in MGO and acrolein drives pathological alterations. Secondly, we employed multiple functional and rescue strategies to establish causality. These include carbonyl quenching with carnosine which restored arginine levels (<u>new data in Fig. 1 of this letter, please see below</u> and new Fig. S3D), as well as arginine supplementation, which attenuated the increase in glucose levels induced by MG and ACR co-treatment (Figs. 6G), demonstrating that arginine depletion functionally contributes to carbonyl stress-induced hyperglycemia. Third, transcriptional changes (RNA-seq and qPCR) are presented as supportive evidence, consistent with the biochemical and functional data, rather than as the primary basis for mechanistic conclusions. As mentioned above, addressing the full complexity

of the network in different tissues and organs systematically, including identifying several reactive carbonyls and the activities of detoxification enzymes, is a substantial long-term effort that goes beyond the scope of a single study. In this respect, it should also be noted that not all reactive carbonyls have probably been identified. Furthermore, one detoxifying enzyme can detoxify several carbonyls, which would make analyzing the complexity of carbonyls even more challenging.
To address NO-related mechanisms more directly, we complemented gene expression analyses with (i) measurements of nitrate/nitrite levels (<u>new data in Fig. 4 of this letter, please see below</u> and new Fig. S3A), (ii) pharmacological inhibition of NO synthesis using L-NAME (Figs. 6F, S13), (iii) NO donation using SNP (Figs. 7D-F, S12), and (iv) quantification of arginine levels across tissues (Figs. 2D,E,H, S3D, S4A, S11), in addition to rescue experiments via arginine supplementation (Figs. 6, 7A-C, S11C).
Together, these complementary approaches provide converging evidence for a biologically meaningful role of carbonyl stress and NO-related pathways in the DKO mutants. While further studies will be valuable to dissect the full complexity of the underlying network in different tissues and organs, the data presented here support our mechanistic conclusions beyond correlative transcriptional changes in a physiological setting of carbonyl stress.

I also find the authors' interpretation of a potential hormetic effect of carbonyls unclear and unconvincing. If hormesis were indeed occurring, one would expect beneficial effects associated with the small increases observed in the knockout (KO) model. The manuscript does not provide a clear rationale for the interpretation presented.
<u>Reply:</u>
We thank the reviewer for this comment and agree that our previous discussion of hormesis was unclear and potentially misleading. We do not propose hormesis as the underlying mechanism for the observed phenotype. Instead, our data support a model of chronic, sustained elevation of reactive carbonyl species (MGO and acrolein), resulting in cumulative metabolic and signaling adaptations rather than a transient beneficial response. Notably, we do not observe evidence of improved function or protective effects in the DKO model that would be expected under a hormetic framework. The previous reference to hormesis was intended to acknowledge literature describing potential beneficial effects of low concentrations of reactive carbonyl species, primarily in in vitro settings; however, we agree that this concept is not supported by our data.

The central hypothesis of the manuscript is that arginine levels decrease due to carbonyl-mediated modifications. However, based on the response to my Question 2, it appears that the observed decrease in arginine can be explained by multiple metabolic pathways unrelated to arginine modification. It therefore seems likely that arginine modification by carbonyls contributes only minimally to the observed decline in arginine levels.
<u>Reply:</u>
We thank the reviewer for this important comment. To directly assess the contribution of reactive carbonyl species to the arginine decrease, we treated wildtype and DKO animals with carnosine, a scavenger of methylglyoxal and acrolein, and subsequently measured arginine levels. Carnosine treatment substantially reversed the decrease in arginine levels in DKO mutants (<u>see Fig. 1 of this letter</u> and new Suppl. Fig 3D), indicating that carbonyl stress is the major contributing factor of arginine modification. However, the levels of arginine in the carnosine-treated DKO mutants remained lower than in the wildtype controls. This supports the reviewer's point and our previously added data to the manuscript, which showed that additional mechanisms, such as altered arginine metabolism (key enzymes, including ass1, asl, arg1 and odc1, were all reduced in DKO liver, Fig. 4A–D), also contribute to the phenotype. Using a complementary approach, we had previously demonstrated that the combined treatment of MGO and acrolein reduced arginine levels of wildtype animals (Fig. 2H) to a similar extent to that observed in DKO larvae (Fig. 2D), and the arginine supplementation

attenuated the increase in glucose levels induced by MG and ACR co-treatment (Figure 6G). In conclusion, our data support a model in which increased levels of reactive carbonyl species contribute to altered arginine homeostasis through the direct modification of arginine, as evidenced by increased MG-H1 levels and decreased free arginine concentrations, as well as altered arginine related pathways (Fig. 2D-H). Consistent with the functional relevance of arginine depletion, arginine supplementation rescued key metabolic and biochemical phenotypes in DKO mutants, including glucose levels, AKT phosphorylation, the expression of genes involved in glucose metabolism and kidney alteration (Fig. 6-7). We have revised the title and text to clarify that arginine modification represents an important, but not exclusive, mediator of the observed effects (manuscript page 1, 6 and 10).

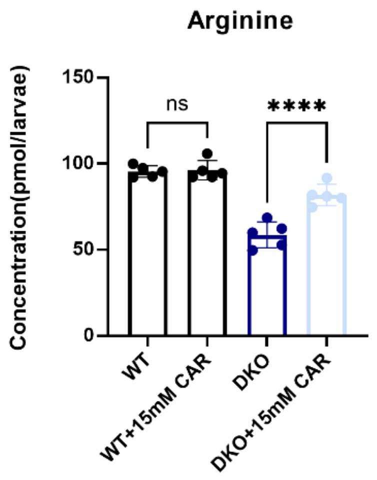

*Fig. 1: Carnosine (CAR) treatment prevented the decrease in arginine levels in DKO mutants. The bars indicate mean±SD values, n = 5. Statistical analysis was applied by one-way ANOVA. ns, not significant, ****p < 0.0001. CAR, carnosine.*

Another unresolved issue concerns skeletal muscle. It remains puzzling that little to no effect is observed in muscle tissue, despite the fact that glucose metabolism predominantly occurs there. The explanation provided in response to Question 3 is not convincing and does not adequately resolve this discrepancy.

Reply:

We agree that skeletal muscle is responsible for the majority of postprandial glucose disposal. At first glance, the lack of detectable arginine depletion in muscle appears to be inconsistent with the postprandial hyperglycemia observed in DKO animals. However, our data suggest that an increase in methylglyoxal and acrolein leads to arginine depletion and inhibits arginine biosynthetic pathways in the livers of DKO animals. These two mechanisms reduce systemic arginine levels, which subsequently lowers insulin. Specifically:

1. *We present new evidence that insulin signaling is impaired, as demonstrated by reduced AKT phosphorylation in the livers and muscles of DKO zebrafish under postprandial conditions (see Fig. 2 of this letter and new Figs. 4K-N). The new data are consistent with the data previously presented for DKO larvae (Fig. 6C-D). This suggests that compromised muscle glucose uptake is not due to local arginine deficiency, as arginine levels remained unchanged in muscle tissue (Fig. S4A), but rather due to reduced systemic insulin signaling.*
2. *To prove reduced systemic insulin signaling, we performed an additional experiment to determine insulin levels in DKO larvae using an ELISA. Consistent with our previous qPCR data (Fig. 4G), we observed a significant reduction in whole-body insulin levels in DKO larvae, which could be rescued by arginine supplementation (see Fig. 3 of this*

letter and new Fig. 6E). As insulin is a circulating hormone, a decrease in its level would be expected to impair insulin signaling and glucose disposal in peripheral tissues, including skeletal muscle. Previous data has already shown that, as a rescue strategy, arginine supplementation increases AKT activation (Fig. 6C–D) and decreases glucose levels (Fig. 6A) in DKO larvae.

3. *Unlike in skeletal muscle, our data revealed a decrease in arginine levels (see Fig. 2E) in the liver, which is particular important for arginine metabolism. Key enzymes of the arginine biosynthetic enzymes, including ass1, asl, arg1 and odc1, were all reduced in DKO liver but not in DKO muscle (Fig. 4A–D and Suppl. Fig. 4C-F), indicating tissue-specific arginine metabolism reprogramming and explaining the reduced arginine concentrations in the liver but not in muscle. Consistent with this observation, akr1a1a expression is ~200-fold higher in the liver than in skeletal muscle (DOI: 10.1002/advs.202101281). This suggests that perturbations in this pathway would have a substantially greater metabolic impact on arginine metabolism in hepatic tissue. Consequently, a reduction in hepatic arginine most likely reflects a subsequent reduction in systemic arginine availability. This contributes to impaired insulin signaling, which affects muscle as well.*

Taken together, our data suggest that the observed postprandial hyperglycemia arises from arginine modification (MG-H1) and arginine reduction in the liver. These changes are consistent with reduced insulin levels, which in turn contribute to impaired insulin signaling and glucose uptake in skeletal muscle. We have revised the discussion section to clarify this point and to acknowledge that the precise mechanisms linking tissue-specific arginine metabolism reprograming to systemic insulin regulation require further investigation (manuscript page 10-11).

A.

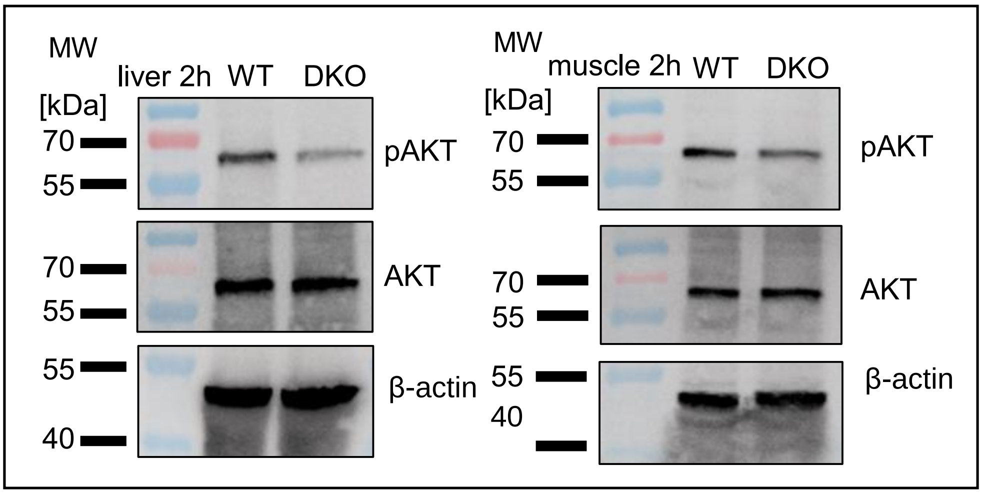

B.

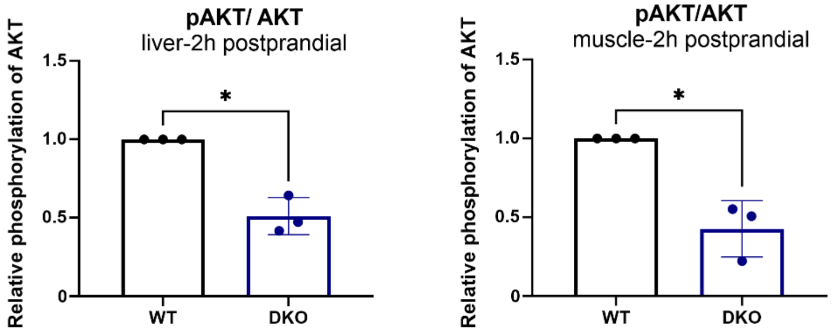

C.

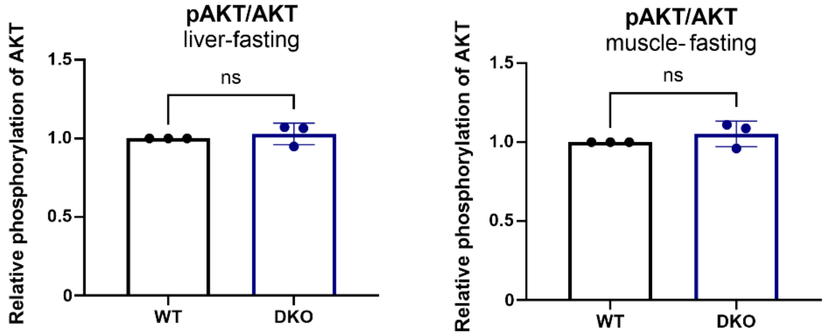

*Fig. 2: (A-B) Reduced postprandial AKT phosphorylation and (C) unchanged fasting AKT phosphorylation in DKO liver and DKO muscle. Upper bands are Phospho-AKT, middle total-AKT and lower β-actin. The bars indicate mean±SD values, n = 3. Statistical analysis was applied by Student's t-test. ns, not significant, *p < 0.05.*

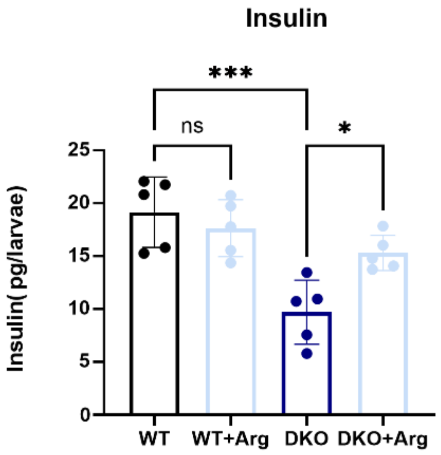

*Fig. 3: Decreased insulin levels in DKO larvae was restored by arginine supplementation. The samples were analyzed by an ELISA. The bars indicate mean±SD values, n = 5. Statistical analysis was applied by one-way ANOVA. ns, not significant, *p < 0.05, ***p < 0.001. Arg, arginine.*

In Question 4, I asked about the detection of nitric oxide. It remains unclear why NO cannot be measured directly using the Griess reaction.

Reply:
We measured nitric oxide using the Griess reaction and found reduced levels of NO_3^- + NO_2^- in DKO mutants (Fig. 4 of this letter and new Supp. Fig. 3A). This further supports our observations that decreased arginine levels lead to reduced nitric oxide.

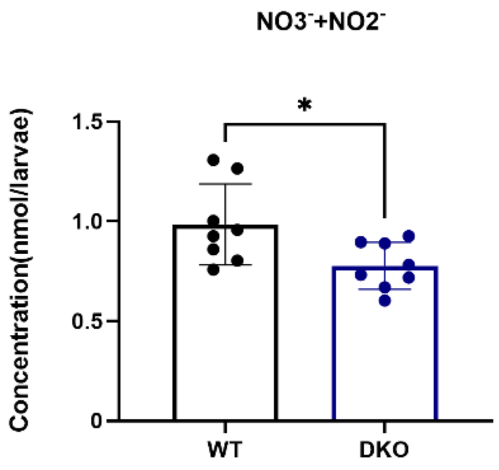

*Fig. 4: Reduced Nitrate + Nitrite concentrations in DKO zebrafish. The bars indicate mean±SD values, n = 8. Statistical analysis was applied by Student's t-test. *$p < 0.05$.*

Finally, in Question 5 I requested a more thorough characterization of renal pathology using additional histological, ultrastructural, and molecular analyses. However, the authors again present only PCR-based data. This does not adequately address the request for deeper pathological characterization.
Reply:
To support our data, we reanalyzed electron microscopy sections for podocyte effacement, a hallmark of diabetic nephropathy, and found increased effacement in the kidneys of DKO zebrafish. Please refer to Fig. 5 of this letter and to new Fig. 5F.

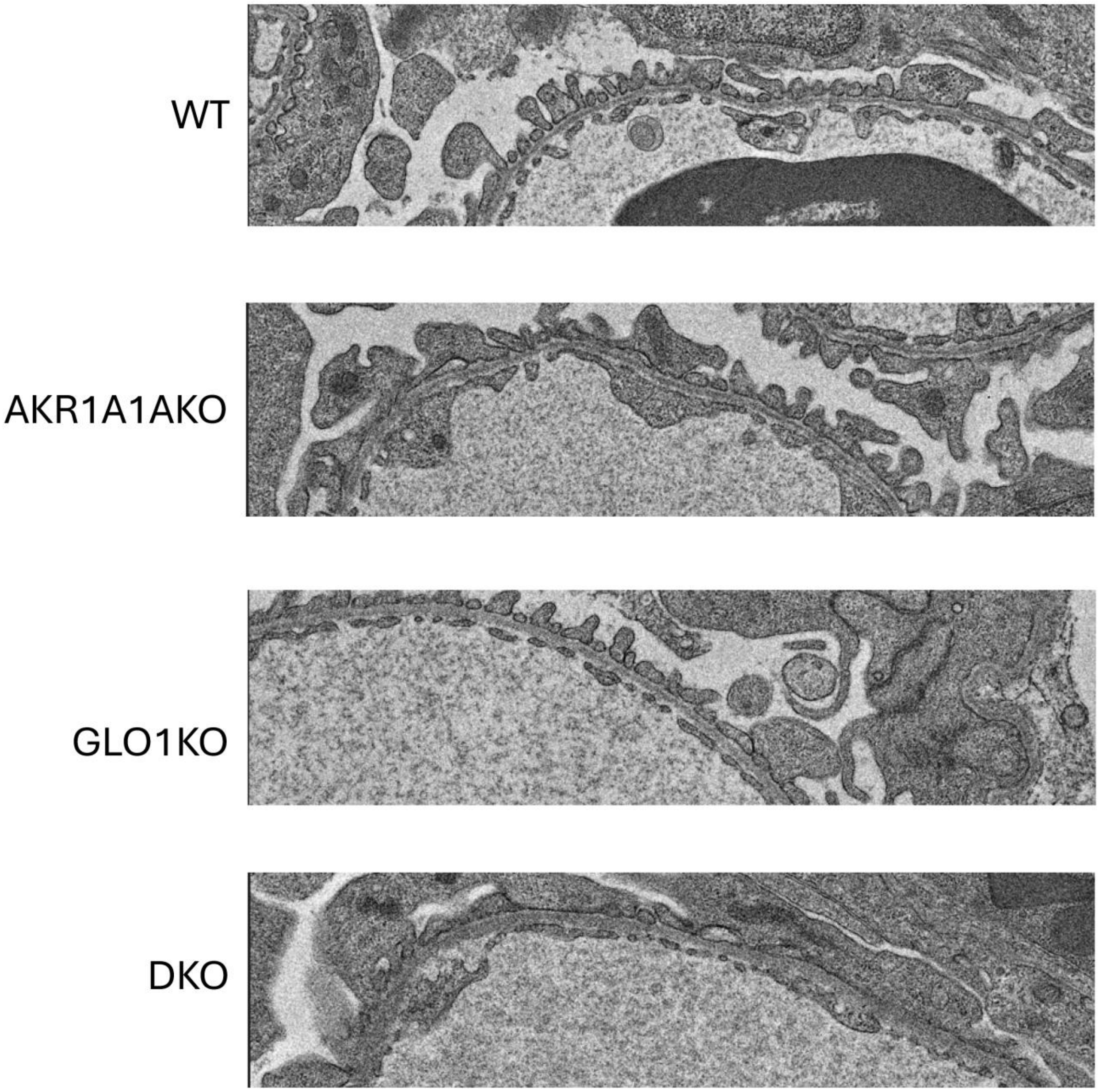
WT
AKR1A1AKO
GLO1KO
DKO

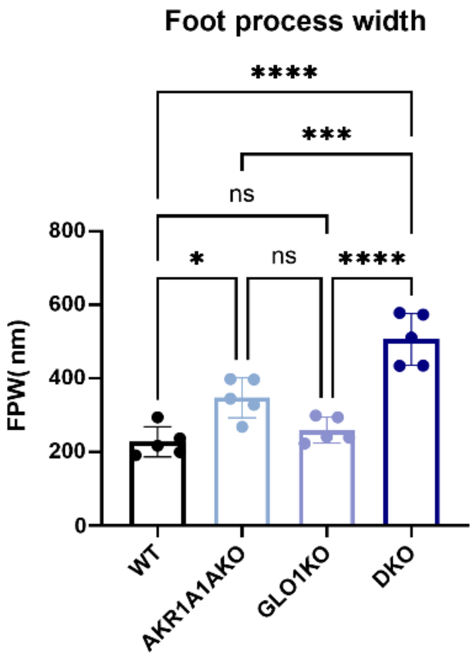

*Fig. 5: Increased podocyte effacement in DKO zebrafish kidney. The bars indicate mean±SD values, n = 5. Statistical analysis was applied by one-way ANOVA. ns, not significant, *p < 0.05, ***p < 0.001, ****p < 0.0001. FPW, foot process width.*

In addition, we performed histological analyses of zebrafish kidneys using hematoxylin and eosin (HE) staining, periodic acid-Schiff (PAS) staining, and Masson trichrome staining. These analyses revealed altered glomeruli, including diffuse matrix expansion and increased cell number, as well as increased collagen deposition, in GLO1/AKR1A1A DKO adult kidneys compared to wildtype (WT) kidneys (Fig. 6 of this letter and new Figure S7A–C). Lastly, we performed an additional antibody staining for type IV collagen. Although the antibody produced a good result on mouse kidney sections (Fig. 7 of this letter), unfortunately it did not generate a specific signal on zebrafish kidneys.

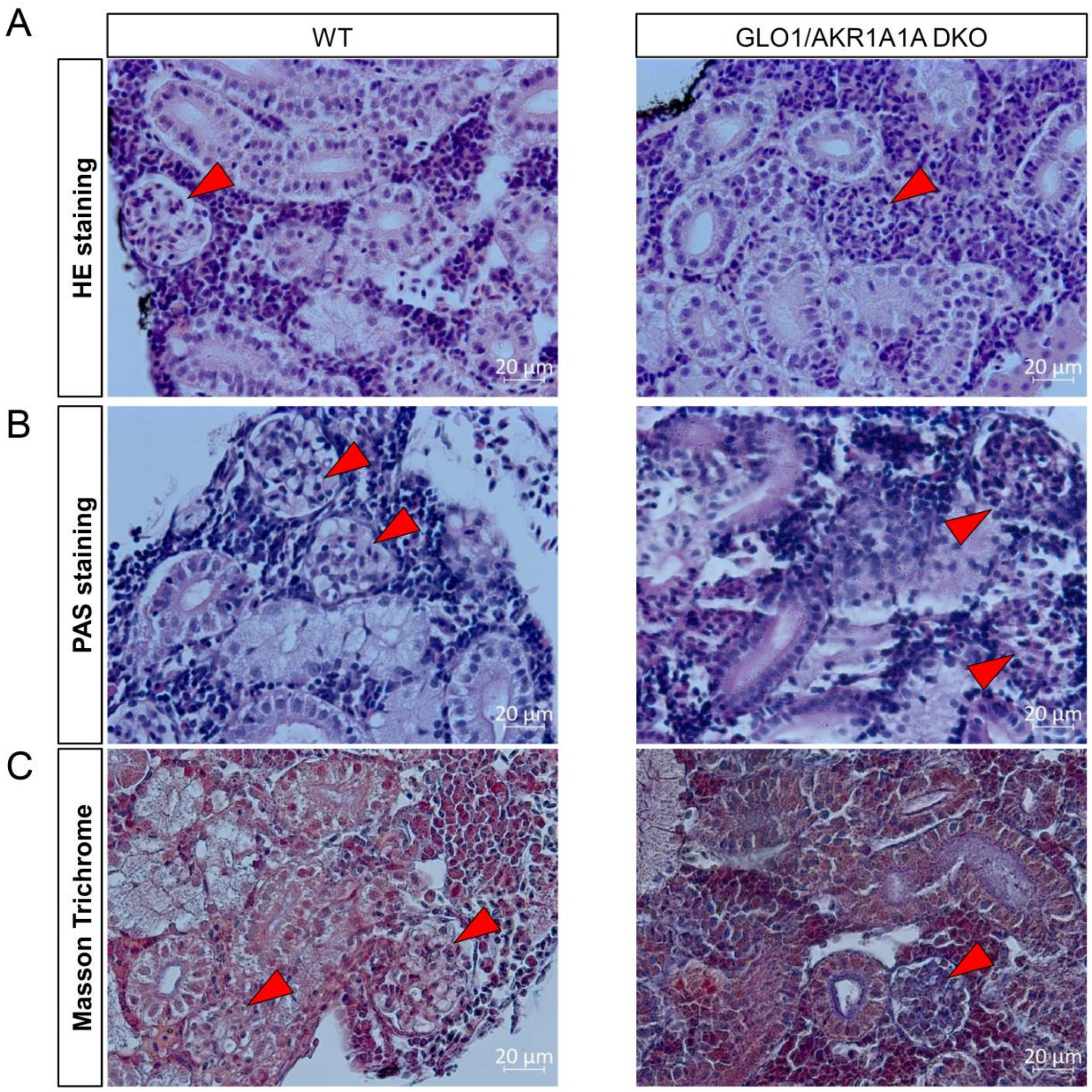

Fig. 6: Altered glomeruli in adult DKO zebrafish kidneys. (A) Representative HE-stained and (B) PAS-stained images showed altered glomeruli with increased cell number and diffuse matrix expansion in GLO1/AKR1A1A DKO adult kidney compared to WT kidney. (C) Representative Masson trichrome–stained images showed increased collagen deposition (blue) in the GLO1/AKR1A1A DKO adult kidney, compared to the wildtype kidney. Red arrows indicate glomeruli. White scale bar = 20µm. HE, hematoxylin and eosin; PAS, periodic acid-Schiff.

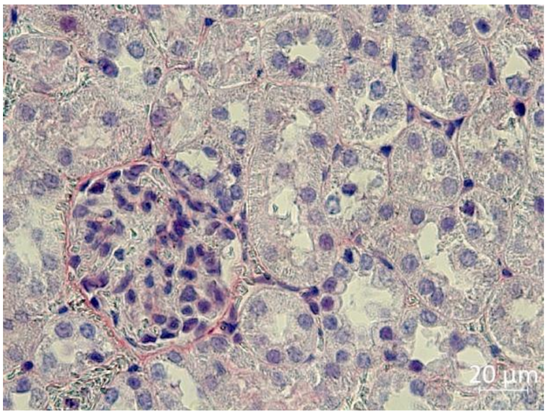

Fig. 7: The representative type IV collagen-stained image shows specific signals of the mouse kidneys. White scale bar = 20μm.

Reviewer #2 (Remarks to the Author):

The authors have satisfactory answered to my points
Reply:
We would like to thank the reviewer for the supportive comments.

Reviewer #3 (Remarks to the Author):

The authors have satisfactorily addressed my concerns.
Reply:
We would like to thank the reviewer for the supportive comments.

REVIEWER COMMENTS

Reviewer #2 (Remarks to the Author):

Review Comments

I have gone through the responses to reviewer 1, and in my opinion the authors have throughly answered to all main points, which were valid and improved the manuscript. I agree the reviewer 1 (and the authors) that this study is restricted to (or focused on) certain enzymes and not the whole network of enzymatic systems, which I also eluded to earlier given that the double knockout is not generating a synergistic genetic interaction. The authors have now adjusted the text, added a series of high quality experiments, and justified their claims in a way that better reflect the compiled findings presented. Overall, this is a well preformed study that will add important information to the present literature.

Reply:

We are grateful to the comments of reviewer 2 and have no further comments to add.
